# Immunoglobulins or Antibodies: IMGT^®^ Bridging Genes, Structures and Functions

**DOI:** 10.3390/biomedicines8090319

**Published:** 2020-08-31

**Authors:** Marie-Paule Lefranc, Gérard Lefranc

**Affiliations:** IMGT^®^, The International ImMunoGeneTics Information System^®^, Laboratoire d’ImmunoGénétique Moléculaire LIGM, Institut de Génétique Humaine IGH, Université de Montpellier UM, Centre National de la Recherche Scientifique CNRS, UMR 9002 CNRS-UM, 141 Rue de la Cardonille, CEDEX 5, 34396 Montpellier, France

**Keywords:** IMGT, immunogenetics, immunoinformatics, immunoglobulin, antibody, IG and TR antigen receptors, IMGT-ONTOLOGY, IMGT Collier de Perles, IMGT unique numbering, adaptive immune repertoire

## Abstract

IMGT^®^, the international ImMunoGeneTics^®^ information system founded in 1989 by Marie-Paule Lefranc (Université de Montpellier and CNRS), marked the advent of immunoinformatics, a new science at the interface between immunogenetics and bioinformatics. For the first time, the immunoglobulin (IG) or antibody and T cell receptor (TR) genes were officially recognized as ‘genes’ as well as were conventional genes. This major breakthrough has allowed the entry, in genomic databases, of the IG and TR variable (V), diversity (D) and joining (J) genes and alleles of *Homo sapiens* and of other jawed vertebrate species, based on the CLASSIFICATION axiom. The second major breakthrough has been the IMGT unique numbering and the IMGT Collier de Perles for the V and constant (C) domains of the IG and TR and other proteins of the IG superfamily (IgSF), based on the NUMEROTATION axiom. IMGT-ONTOLOGY axioms and concepts bridge genes, sequences, structures and functions, between biological and computational spheres in the IMGT^®^ system (Web resources, databases and tools). They provide the IMGT Scientific chart rules to identify, to describe and to analyse the IG complex molecular data, the huge diversity of repertoires, the genetic (alleles, allotypes, CNV) polymorphisms, the IG dual function (paratope/epitope, effector properties), the antibody humanization and engineering.

**Table of content:**

**1. Introduction**
**2. Immunoglobulin (IG) or Antibody Molecular Genetics**
*2.1. Human Immunoglobulin (IG) or Antibody Structure and Dual Function*
2.1.1. Immunoglobulin Fab and Fc Basic Structure
2.1.2. B Cell Differentiation
2.1.3. Membrane Immunoglobulins and B Cell Receptor
2.1.4. Secreted IG
*2.2. Human IG Classes and Subclasses: Heavy and Light Chain Types*
2.2.1. IG Heavy Chain Types
2.2.2. IG Light Chain Types
*2.3. IG Chain Variable and Constant Domains*
2.3.1. IG Variable Domains
2.3.2. IG Constant Domains
*2.4. Synthesis and Expression of the Immunoglobulins (IG) or Antibodies*
2.4.1. IG Molecular Synthesis Characteristics
2.4.2. Synthesis of the H-mu Chains: D-J and V-D-J Rearrangements in the IGH Locus
2.4.3. Synthesis of the L-kappa and L-lambda Chains: V-J Rearrangements in the IGK and IGL Loci
*2.5. Origin of the Variable Domain Diversity of the Immunoglobulins*
2.5.1. Overview
2.5.2. Combinatorial Diversity
2.5.3. Junctional Diversity
2.5.4. Somatic Hypermutations
*2.6. Expression of the Heavy Chains and IG Classes*
2.6.1. Coexpression of the Membrane H-mu and H-delta Chains
2.6.2. Expression of H-gamma, H-epsilon and H-alpha chains: Class Switch Recombination
2.6.3. Expression of H-delta Chains from IgM^−^ IgD^+^ Cells
2.6.4. Expression of Membrane and Secreted Immunoglobulins
*2.7. Regulation of the Rearrangements and Chain Expression*
2.7.1. Allelic and Isotypic Exclusion. Rearrangement Chronology
2.7.2. Regulation of the IG Gene Expression: Enhancers
*2.8. Structural and Biological Properties of the Secreted Immunoglobulins*
2.8.1. IgM
2.8.2. IgD
2.8.3. IgG
2.8.4. IgA
IgA1 and IgA2
Secretory IgA
IgA Effector Function
2.8.5. IgE
**3. Immunoglobulin Genes: IMGT^®^ Gene and Allele Nomenclature**
*3.1. IMGT^®^ Standardized Genes and Alleles (CLASSIFICATION)*
3.1.1. IG and TR Genes and Concepts of Classification: Birth of IMGT^®^ and Immunoinformatics
3.1.2. *Homo sapiens* IG Genes and Concepts of Identification and Description
*3.2. Homo sapiens IGH Locus and Genes*
3.2.1. Organization of the *Homo sapiens* IGH Locus
3.2.2. IGHC Multigene Deletions and Gene Order, IGHC and IGHV Copy Number Variation (CNV) Haplotypes
3.2.3. IGH Orphons
3.2.4. Potential *Homo sapiens* IGH Genomic Repertoire
*3.3. Homo sapiens IGK Locus and Genes*
3.3.1. Organization of the *Homo sapiens* IGK Locus
3.3.2. IGK Orphons
3.3.3. Potential *Homo sapiens* IGK Genomic Repertoire
3.3.4. *Homo sapiens* IGKC Allotypes (Km Alleles)
*3.4. Homo sapiens IGL Locus and Genes*
3.4.1. Organization of the *Homo sapiens* IGL Locus
3.4.2. IGL Orphons
3.4.3. Potential *Homo sapiens* IGL Genomic Repertoire
3.4.4. *Homo sapiens* IGL Isotypes
**4. Immunoglobulin Structures: IMGT Unique Numbering and IMGT^®^ Collier de Perles**
*4.1. IMGT Unique Numbering and IMGT Colliers de Perles for V-DOMAIN (NUMEROTATION)*
4.1.1. V Domain Definition and Main Characteristics
4.1.2. V-DOMAIN IMGT Colliers de Perles
4.1.3. V-DOMAIN Strands and Loops (FR-IMGT and CDR-IMGT)
4.1.4. V-DOMAIN Conserved Amino Acids
4.1.5. V-DOMAIN Delimitation
4.1.6. Protein Displays for the V-REGION and J-REGION
*4.2. IMGT Unique Numbering and IMGT Colliers de Perles for C Domain (NUMEROTATION)*
4.2.1. C Domain Definition and Main Characteristics
4.2.2. C Domain IMGT Colliers de Perles
4.2.3. C Domain Strands and loops
4.2.4. C Domain Conserved Amino Acids
4.2.5. C Domain Genomic Delimitation
4.2.6. C-REGION Protein Displays
**5. IMGT^®^ Databases and Tools for IG Sequences and Structures**
*5.1. IMGT^®^, the International ImMunoGeneTics Information System^®^*
*5.2. IMGT^®^ Nucleotide Sequence and Repertoire Analysis*
5.2.1. IMGT/V-QUEST for Nucleotide Sequence Analysis
5.2.2. IMGT/HighV-QUEST
*5.3. IMGT^®^ Amino Acid Sequence Analysis and Representation*
5.3.1. IMGT/DomainGapAlign
5.3.2. IMGT/Collier-de-Perles Tool
*5.4. IMGT^®^ Structure and Amino Acid Databases*
5.4.1. IMGT/3Dstructure-DB
5.4.2. IMGT/2Dstructure-DB
5.4.3. IMGT/mAb-DB
**6. Using the IMGT Numbering for V and C-Domain for Antibody Description and Engineering**
*6.1. Antibody V-DOMAIN Humanization by IMGT-CDR Grafting*
6.1.1. CDR-IMGT Delimitation for Grafting
6.1.2. Amino acid Interactions between FR-IMGT and CDR-IMGT
*6.2. Only-Heavy-Chain Antibodies*
6.2.1. Dromedary IgG2 and IgG3
6.2.2. Human Heavy Chain Diseases (HCD)
6.2.3. Nurse Shark IgN
*6.3. Contact Analysis of TR-Mimic Antibodies and TR*
*6.4. Antibody C-Domain Post-Translational Modifications, Engineering and Allotypes*
6.4.1. N-linked Glycosylation Sites CH2 N84.4
6.4.2. Knobs-into-Holes
6.4.3. Interface Ball-and-Socket-Like Joints
6.4.4. IGHG Alleles and Gm Allotypes
6.4.5. IGHG Engineered Variants and Effector Properties
**7. Conclusions**

## 1. Introduction

The adaptive immune response is found in all extant vertebrate species from fishes to humans and was acquired by jawed vertebrates or *gnathostomata* more than 450 million years ago. It is characterized by a remarkable immune specificity and memory which are the properties of the B and T cells, and by an extreme diversity of their specific antigen receptors [1]. These antigen receptors of the adaptive immune response comprise the immunoglobulins (IG) or antibodies of the B cells and plasmocytes [2], and the T cell receptors (TR) of the T cells [3]. The IG recognize antigens in their native (unprocessed) form, whereas the TR recognize processed antigens, which are presented as peptides by the highly polymorphic major histocompatibility (MH, in humans HLA for human leucocyte antigens) proteins. Immunoglobulins or antibodies serve a dual role in immunity: they both recognize antigens on the surface of foreign bodies such as bacteria and viruses, and trigger elimination mechanisms such as cell lysis and phagocytosis, to rid the body of these invading cells and particles [2]. The potential antigen receptor repertoire of each individual is estimated to comprise about 2 × 10^12^ different IG and TR, and the limiting factor is only the number of B and T cells that an organism is genetically programmed to produce [1] This huge diversity results from the complex molecular synthesis of the IG and TR chains and more particularly of their variable domains which, at their N-terminal end, recognize and bind the antigens [2,3].

IMGT^®^, the international ImMunoGeneTics information system^®^ (http://www.imgt.org) [1], was created in 1989 by Marie-Paule Lefranc at Montpellier, France, Laboratoire d’ImmunoGénétique Moléculaire (LIGM) des Prof G. and M-P. Lefranc (Université de Montpellier and CNRS) to manage the huge diversity of the IG and TR repertoires. For the first time, immunoglobulin (IG) or antibody and T cell receptor (TR) variable (V), diversity (D), joining (J) and constant (C) genes were officially recognized as ‘genes’ as well as were conventional genes [2,3,4,5]. By its creation, IMGT^®^ marks the advent of immunoinformatics, which emerged at the interface between immunogenetics and bioinformatics [1]. The IMGT^®^ information system has been online since 1995 (first Internet connexion of IMGT/LIGM-DB occurred at the 9th International Congress of Immunology (ICI) in San Francisco, CA (USA), 23–29 July 1995), marking the 7-year anniversary of the first Internet France-USA connexion on July 28, 1988). IMGT^®^ [6,7,8,9,10,11,12,13,14,15,16,17,18] is an integrated system for genes, sequences and structures of the IG or antibodies, TR and MH of the adaptive immune responses of the jawed vertebrates, as well as of other proteins of the IG superfamily (IgSF) [19] and MH superfamily (MhSF) of vertebrates and invertebrates [20]. Immunoinformatics [1] builds and organizes molecular immunogenetics knowledge to be managed and shared in IMGT^®^. The accuracy and consistency of IMGT^®^ data and the coherence between the different IMGT^®^ components (databases, tools and Web resources), are based on the IMGT Scientific chart rules, generated from the seven axioms and concepts of IMGT-ONTOLOGY, the first ontology for immunogenetics and immunoinformatics [21,22,23,24,25,26,27,28,29]. As examples, IMGT Scientific chart rules comprise the IMGT^®^ standardized keywords (IDENTIFICATION axiom [30,31] and concepts of identification), the IMGT^®^ standardized labels [32,33,34] (DESCRIPTION axiom [34,35] and concepts of description), the IMGT^®^ gene and allele nomenclature [1,2,3,4,5,36] (CLASSIFICATION axiom [37,38] and concepts of classification), the IMGT unique numbering [39,40,41,42,43,44,45,46] and IMGT Colliers de Perles [47,48,49,50,51,52] (NUMEROTATION axiom [53] and concepts of numerotation). 

The IMGT**^®^** information system comprises seven databases [54,55,56,57,58,59,60], 17 tools [61,62,63,64,65,66,67,68,69,70,71,72,73,74,75,76,77,78,79,80] and more than 25,000 pages of Web resources. IMGT**^®^** dababases are specialized in sequences (i.e, IMGT/LIGM-DB [54,55]), genes and alleles (IMGT/GENE-DB [56]), two-dimensional (2D) (IMGT/2Dstructure-DB) and three-dimensional (3D) structures (IMGT/3Dstructure-DB) [57,58,59]. IMGT/mAb-DB [60] allows querying therapeutic monoclonal antibodies (IG, mAb), fusion proteins for immunological applications (FPIA), composite proteins for clinical applications (CPCA) and related proteins (RPI) of therapeutic interest (with links to amino acid sequences in IMGT/2Dstructure-DB and, if available, to 3D structures in IMGT/3Dstructure-DB). IMGT**^®^** tools include (i) for nucleotide sequence analysis, IMGT/V-QUEST [61,62,63,64,65,66] and the integrated IMGT/JunctionAnalysis [67,68] and IMGT/Automat [69,70] tools, and for next generation sequencing, the high-throughput version IMGT/HighV-QUEST [66,71,72,73,74,75] and the downloadable IMGT/StatClonotype [76,77] package (statistical pairwise analysis of the diversity and expression of the IMGT clonotypes (AA) [73] and repertoire comparison in adaptive immune responses), (ii) for genomic analysis, IMGT/LIGMotif [78] (identification and description of new genes in genomic sequences), (iii) for amino acid sequence analysis per domain, IMGT/DomainGapAlign [58,79,80], and (iv) for graphical representations of domains, the IMGT/Collier-de-Perles tool [51] (e.g., IMGT Colliers de Perles of the variable (V), constant (C), groove (G) domains) [39,40,41,42,43,44,45,46,47,48,49,50,51,52]). IMGT^®^ Web resources (‘the IMGT Marie-Paule page’) comprise the IMGT Repertoire (IG and TR, MH and RPI), IMGT Scientific chart, IMGT Education (IMGT Lexique, Aide-mémoire (amino acid physicochemical properties [81], splicing sites), tutorials, etc.). Since 2003, IMGT^®^ has been widely used in the analysis of therapeutic antibodies for humanization and/or engineering [6,9,82,83,84,85,86,87,88,89,90,91,92,93,94,95,96,97,98,99,100]. The IMGT nomenclature has been endorsed by the International Union of Immunological Societies (IUIS) [84,85] and used, since 2006, in the description of the therapeutic antibodies published by the World Health Organisation (WHO) International Nonproprietary Names (INN) programme [86,87]. 

We first present the immunoglobulin (IG) or antibody structure and dual function based on molecular genetics in humans. We then highlight the two founding breakthroughs of immunoinformatics, the IMGT^®^ IG and TR gene and allele nomenclature (illustrated here, by the description of the *Homo sapiens* IGH, IGK and IGL loci), and the IMGT unique numbering and IMGT^®^ Collier de Perles (illustrated here, by the IG V and C domains). We then show how IMGT^®^ databases and IMGT^®^ tools intrinsically bridge IG and TR genes, sequences and structures, according to three axes, the genomic locus description from fish to humans, the analysis of the huge diversity of the adaptive immune repertoires, the IG and TR engineering for therapeutical and clinical applications. Using IMGT state of the art, we illustrate with immunoglobulin (IG) or antibody examples. 

## 2. Immunoglobulin (IG) or Antibody Molecular Genetics

### 2.1. Human Immunoglobulin (IG) or Antibody Structure and Dual Function

#### 2.1.1. Immunoglobulin Fab and Fc Basic Structure 

**Figure 1:**
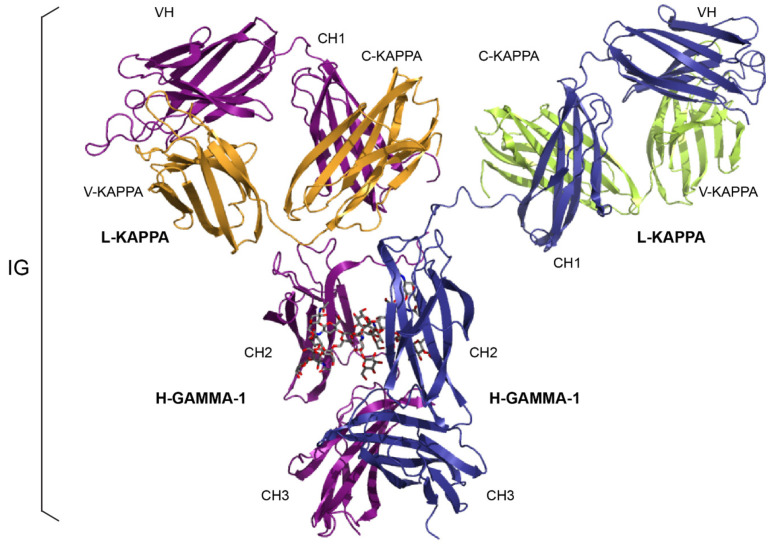
An immunoglobulin (IG) or antibody. The structure is that of the antibody b12, an IgG1-kappa, and so far the only complete human IG crystallized (IMGT^®^ http://www.imgt.org, IMGT/3Dstructure-DB > Entry code (PDB): 1hzh). H-GAMMA-1 and L-KAPPA (for the chains), VH, CH1, CH2, CH3, V-KAPPA and C-KAPPA (for the domains) written in capital letters are IMGT standardized labels (DESCRIPTION) [1,34,35]. H-gamma1 and L-kappa, written in small letters in the text, corresponf to the IMGT standardized keywords (IDENTIFICATION) [1,30,31]. The two light chains are identical and the two heavy chains are identical (the different colours are only used for a better visualization). (With permission from M-P. Lefranc and G. Lefranc, LIGM, Founders and Authors of IMGT^®^, the international ImMunoGeneTics information system^®^, http://www.imgt.org).

The discovery of the action of proteolytic enzymes on IgG1 by Porter in 1959 [101] provided the first great insight into antibody structure. The general Y shaped configuration of an immunoglobulin corresponds to two Fab (fragment antigen binding) arms and an Fc (fragment crystallizable) region (Figure 1). These fragments are obtained by papain-digestion of IgG1 or by recombinant engineering. The immunoglobulins (IG) or antibodies share a basic four-chain structure composed of two identical heavy chains of 50–70 kDa and two identical light chains of 25 kDa. An IG comprises 12 domains (for example, IgG1, Figure 1) or 14 domains (IgM or IgE). The heavy chain (i.e., H-gamma1) is made of a variable heavy (VH) domain at the N-terminal end and of three constant heavy (CH) domains with a hinge between CH1 and CH2 (or four CH domains, for the heavy chains H-mu and H-epsilon) (Table 1). The light chain (L-kappa or L-lambda) is made of a variable light (VL) domain (V-kappa or V-lambda) at the N-terminal end and of a constant domain (C-kappa or C-lambda) at the C-terminal end.

**Figure 2:**
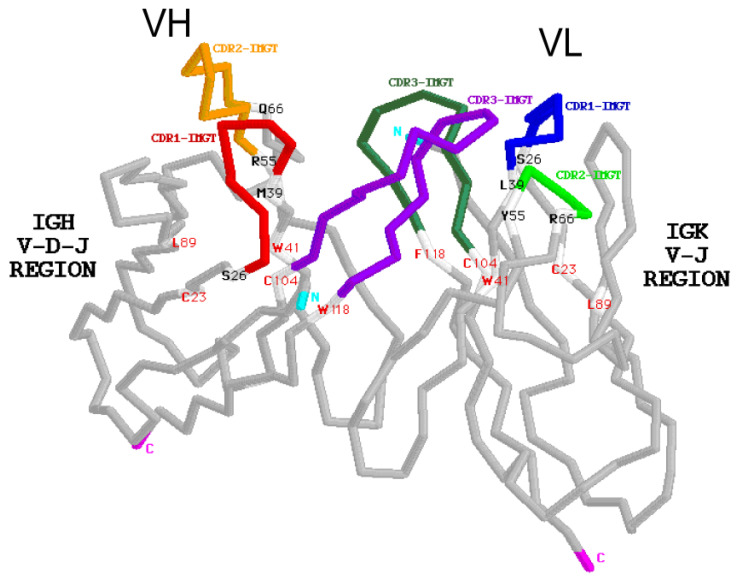
Paired VH and VL domains of an immunoglobulin (IG) or antibody. The VH and VL domains are encoded by a V-D-J-REGION and a V-J-REGION, respectively. The frameworks are colored in grey. The hypervariable loops or complementarity determining regions CDR1-IMGT, CDR2-IMGT and CDR3-IMGT are colored in red, orange and purple for VH, and in blue, green and green-blue for VL, respectively. The six loops of VH and VL constitute the antigen recognition and binding site. The highly conserved amino acids are indicated in red with their position according to the IMGT unique numbering: C23 (1st-CYS), W41 (CONSERVED-TRP), L89 (conserved hydrophobic amino acid), C104 (2nd-CYS 104), W118 (J-TRP) for VH, and F118 (J-PHE) for VL [1,40,41]. 104 and 118 are anchor positions for the CDR3-IMGT (written in red). Anchor positions are, for the CDR1-IMGT, 26 and 39 and, for the CDR2-IMGT, 55 and 66 (written in black). (With permission from M-P. Lefranc and G. Lefranc, LIGM, Founders and Authors of IMGT^®^, the international ImMunoGeneTics information system^®^, http://www.imgt.org).

The two identical Fab arms of an immunoglobulin (IG) or antibody, each comprises the two N-terminal domains VH-CH1 of the heavy chain associated with the light chain (VL-CL). Each Fab arm is involved, by the N-terminal VH and VL domains, in the antigen recognition and binding (Figure 2).

The Fc region, formed by the C-terminal domains CH2-CH3 of the two heavy H-gamma1 chains, interacts with effector molecules such as the complement component C1q and the Fc receptors [2]. The binding of these effector molecules to the Fc of antibodies coated at the surface of foreign antigens trigger elimination processes. Activation of the classical complement cascade generates a variety of potent biological molecules, which promote phagocytosis, chemotaxis and formation of the membrane attack complex, resulting in cell lysis. The pathway is triggered by the interaction of C1, a protein complex of C1q, C1r and C1s, with antigen-antibody complexes. It is the C1q head region which interacts directly with the immunoglobulin Fc. Binding of antibody-antigen complexes or aggregated immunoglobulins to the Fc receptors triggers cell functions which serve important roles against pathogenic agents as well as in the regulation of antibody production.

#### 2.1.2. B Cell Differentiation

**Figure 3:**
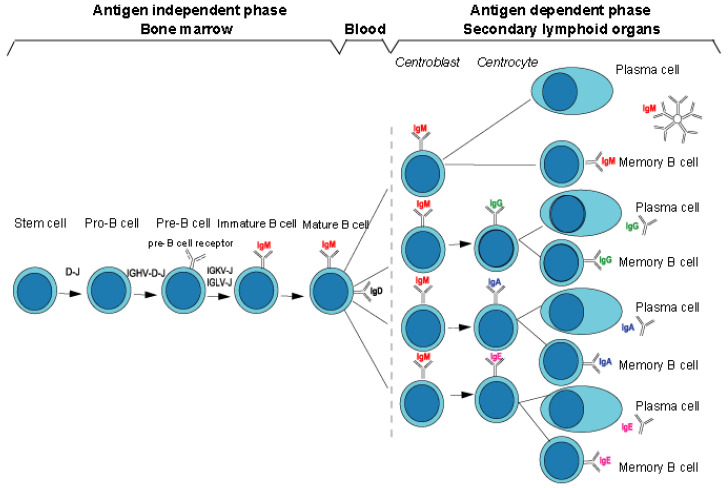
B cell differentiation with the antigen independent phase in the bone marrow and the antigen dependent phase in the secondary lymphoid organs [2]. (With permission from M-P. Lefranc and G. Lefranc, LIGM, Founders and Authors of IMGT^®^, the international ImMunoGeneTics information system^®^, http://www.imgt.org).

Immunoglobulins are expressed as membrane immunoglobulins (mIG) on the surface of the B lymphocytes (mature B cells and memory B cells) as part of a B cell receptor (BcR) or as soluble immunoglobulins secreted by plasma cells [2] (Figure 3).

The different stages of B cell differentiation from the hematopoietic stem cells into mature B cells which express IgM and IgD, occur in the bone marrow and are antigen independent [2] (Figure 3). The final differentiation stages, from the mature B cells into memory cells or plasma cells that express or secrete IG from various classes or subclasses occur in the germinal centers of the secondary lymphoid organs, and are antigen dependent, and generally requires cooperation between B and T cells [2] (Figure 3).

#### 2.1.3. Membrane Immunoglobulins and B Cell Receptor

**Figure 4:**
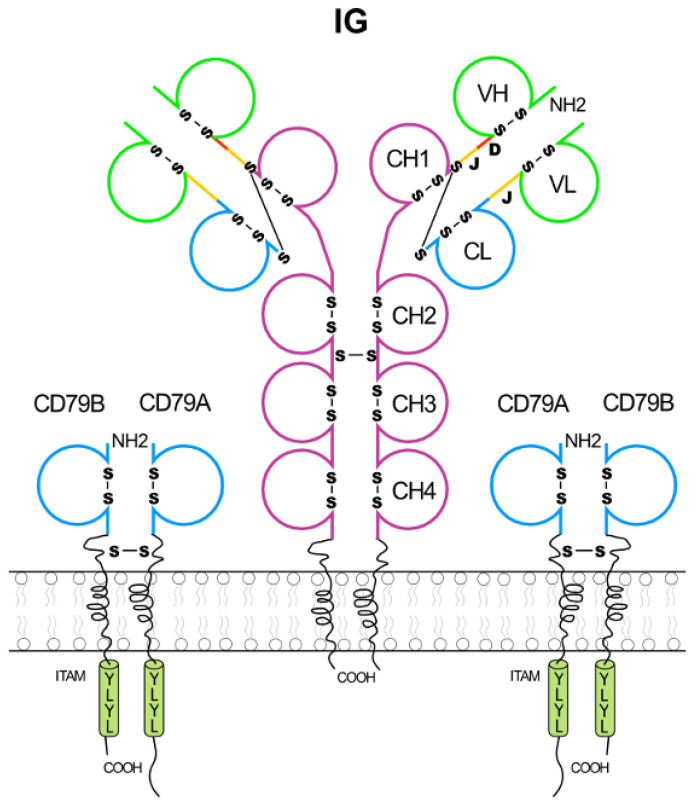
B cell receptor (BcR). The B cell receptor (BcR) on the surface of mature B cells comprises an immunoglobulin (IG) or antibody, here IgM, as a monomer H2L2, anchored in the membrane of the B cell (membrane IG or mIG) and the CD79 signalling coreceptors constituted of two heterodimers CD79A/CD79B (BcR = mIG + CD79 coreceptor). VH, CH1, CH2, CH3 and CH4 indicate the domains of the H-mu chains of the IgM. Depending on the light chain type, L-kappa or L-lambda, VL and CL correspond to V-kappa and C-kappa, or to V-lambda and C-lambda, respectively. ITAM motifs are indicated by the letters YLYL for tyrosyl (Y) and leucyl (L) amino acids. (With permission from M-P. Lefranc and G. Lefranc, LIGM, Founders and Authors of IMGT^®^, the international ImMunoGeneTics information system^®^, http://www.imgt.org).

The B cell reeptor BcR (Figure 4) comprises an antigen recognition unit constituted by a membrane immunoglobulin (mIG), on mature B cells (IgM, IgD) or on memory B cells (IgM, IgA, IgG, IgE), anchored in the membrane and a signalling coreceptor constituted by two heterodimers CD79A (Ig-alpha, mb-1, MB-1)/CD79B (Ig-beta, B29). The CD79A/CD79B dimers ensure the signal transmission when the membrane IG binds to an antigen. The CD79A and CD79B chains are composed of a single IgSF C-like domain and exist at the cell surface as a disulfide-linked heterodimer and contain, in their cytoplasmic region (CY), an immunoreceptor tyrosine-based activation motif (ITAM) (Figure 4) (IMGT^®^ http://www.imgt.org, IMGT Repertoire (RPI) > IMGT RPI entries from gene to protein > IgSF other than IG or TR > CD79A; ibid > CD79B). 

ITAM (indicated by YLYL in Figure 4) are rich in tyrosines and with a consensus (D/E)xxYxx(L/I)x_6–8_Yxx(L/I) [102]. Cross-linking of the BcR induces the tyrosylphosphorylation of the ITAM on the cytoplasmic region of CD79A and CD79B, and the signalling cascade leading to B cell activation, by recruitment of signalling molecules which belong to at least two families of protein tyrosine kinases (PTK), the Syk family and the Tec family, and provide signal transmission.

#### 2.1.4. Secreted IG 

Secreted IgG, IgD and IgE are monomeric, whereas IgM occurs as a pentamer. IgA occurs predominantly as a monomer in the serum and as a dimer in seromucous secretions.

### 2.2. Human IG Classes and Subclasses: Heavy and Light Chain Types

#### 2.2.1. IG Heavy Chain Types

Human immunoglobulins (IG) or antibodies are divided into five classes: IgM, IgD, IgG, IgA and IgE—each with distinct heavy chains (H-mu, H-delta, H-gamma, H-alpha and H-epsilon, respectively) which differ by their physicochemical properties, antigenic determinants, biological functions and effector properties (Table 2) [103,104,105,106,107,108]. 

The IgG and IgA classes in humans are further subdivided into four subclasses (IgG1, IgG2, IgG3 and IgG4) and two subclasses (IgA1 and IgA2), respectively, on their heavy chain characteristics (H-gamma1, H-gamma2, H-gamma3 and H-gamma4, H-alpha1 and H-alpha2). Thus, in humans, there is a total of nine heavy chain isotypes (H-mu, H-delta, H-gamma3, H-gamma1, H-alpha1, H-gamma2, H-gamma4, H-epsilon, H-alpha2) (Table 3). The inter H-L and inter H-H disulfide bridges (numbers and positions of the involved cysteines) characterize the human IG classes or subclasses (Table 4).

The nine isotypes are defined by the heavy chain constant region which is encoded by one of the nine functional IGHC genes (IGHM, IGHD, IGHG3, IGHG1, IGHA1, IGHG2, IGHG4, IGHE and IGHA2), listed in the order 5′–3′ in the IGH locus (IMGT^®^, http//www.imgt.org, IMGT Repertoire (IG and TR)). The kappa or lambda light chains may associate to any heavy chain isotype. Further allotypic variants of certain subclasses or class have also been identified which represent genetic markers for the gamma1, gamma2, gamma3, alpha2 and epsilon heavy chains, and are, consequently called G1m, G2m, G3m, A2m and Em allotypes [83] (IMGT^®^ http://www.imgt.org, IMGT Repertoire (IG and TR) > 2. Proteins and alleles > 4. Allotypes > Human (*Homo sapiens*) IGHC). The allotypes are polymorphic markers detected by serological methods, and are present in different individuals of the same species [83]. The Gm and Am allotypic determinants, inherited in fixed combinations or Gm-haplotypes, are useful tools in the characterization of populations [83,109,110] and genetics of immunoglobulins [111,112,113,114,115,116,117]. 

#### 2.2.2. IG Light Chain Types

The two light chain types, L-kappa and L-lambda, are common to all five classes. Either light chain type can associate with any of the heavy chain types, but in any particular immunoglobulin, both light and both heavy chains are identical. The kappa to lambda ratio in the serum of healthy individuals is approximately 2 to 1. Four lambda isotypes have been identified by the presence or absence of serological markers (Mcg, Kern and Oz) [2] (IMGT^®^ http://www.imgt.org, IMGT Repertoire (IG and TR) > 2. Proteins and alleles > 5. Isotypes: Human (*Homo sapiens*) IGLC). Three Km allotypes have been characterized [83,109,110] (IMGT^®^ http://www.imgt.org, IMGT Repertoire (IG and TR) > 2. Proteins and alleles > 4. Allotypes: Human (*Homo sapiens*) IGKC).

### 2.3. IG Chain Variable and Constant Domains

#### 2.3.1. IG Variable Domains

**Figure 5:**
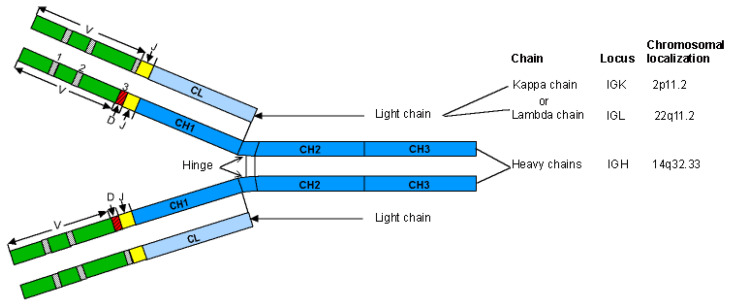
Schematic representation of an immunoglobulin [2]. The two inter heavy chain (inter-H) disulfide bridges correspond to those of a human (*Homo sapiens*) IgG1. The intrachain (Cys 23-Cys 104) disulfide bridge of each of the 12 domains are not shown. The three complementarity regions (CDR1, CDR2 and CDR3) of the VH (V-D-J-region) and of the VL (V-J-region) are indicated by hatches (and numbers 1, 2, 3 for VH). V = V-REGION (in green), J = J-REGION (in yellow), D = D-REGION (or more exactly N-AND-D-REGION to take into account the N-diversity) (in red), the C-REGION is in blue (dark blue for the heavy (H) chain, light blue for the light (L) chain). (With permission from M-P. Lefranc and G. Lefranc, LIGM, Founders and Authors of IMGT^®^, the international ImMunoGeneTics information system^®^, http://www.imgt.org).

The basic structure of an immunoglobulin (IG) or antibody comprises two identical heavy chains, associated with two identical light chains, kappa or lambda (Figure 5). Each chain folds up into domains of approximately 100 to 110 amino acids. There are two domains for a light chain, and four or five domains for a heavy chain. The N-terminal domain of the light and heavy chains is the variable (V) domain which exhibits an enormous diversity between different IG. Each V domain comprises a beta-sheet framework (FR-IMGT) [33] supporting three hypervariable loops or complementarity determining region (CDR-IMGT) 1, 2 and 3 [32], which are spatially close to each other and constitute the recognition and antigen binding site (Figure 2). The variable domain of a light chain, designated V-KAPPA or V-LAMBDA depending on the light chain type, is a V-J-REGION encoded by two rearranged genes (IGKV and IGKJ, or IGLV and IGLJ, respectively) (Table 1).

#### 2.3.2. IG Constant Domains

The other domains, designated as constant (C) domains, are identical between chains from the same class, subclass and with the same allotypes. The constant region or C-REGION of the heavy chain is encoded by one of the IGHC genes, and comprises three or four constant domains (CH1, CH2 and CH3, with a flexible hinge region between CH1 and CH2, for the H-gamma, H-alpha and H-delta chains of the IgG, IgA and IgD, CH1 to CH4 for the H-mu and H-epsilon chains) [118]. The hinge region located between the CH1 and CH2 of the H-gamma chains is encoded by one exon (for H-gamma1, H-gamma2 and H-gamma4) or several exons (2 for H-delta, 2-5 usually 4 for H-gamma3 [119,120]). In IgM and IgE, the CH2 replaces the hinge, and the CH3 and CH4 correspond to the CH2 and CH3 in IgG, IgD and IgA (IMGT^®^ http://www.imgt.org, IMGT Repertoire (IG and TR) > 1. Locus and genes > 5. Gene exon/intron organization IGHC: Human) [2]. The C-REGION of the light chain is encoded by the IGKC gene (for the kappa chains), or one of the IGLC genes (for the lambda chains) [2], and comprises a unique constant domain (C-KAPPA or C-LAMBDA, respectively). 

In intact immunoglobulins, the domains usually associate into pairs through multiple non-covalent lateral interactions. However the CH2 of the IgG, IgD and IgA, and the equivalent CH3 of the IgM and IgE are unpaired but stabilized with interposed N-linked, branched carbohydrate chains. All human IG heavy chains are glycosylated [121] (IMGT^®^ http://www.imgt.org, IMGT Education > IMGT Lexique > Immunoglobulin (IG) or antibody glycosylation). The number of potential N-glycosylation sites per heavy chain is reported in Table 3. The positions of the Asn (N) of the potential N-glycosylation sites are highlighted in the IGHC Protein display (N in green) or are identified by the underlined N-X-S/T (Asn-X-Ser/Thr) motif where X is any AA except Pro). O-glycosylation characterizes the hinge of the H-delta and H-alpha1 chains. 

The molecular structure of the IG chains in domains (12 for IgG, IgD and IgA, 14 for IgM and IgE) is extensively used for the construction and expression of engineered antibodies (IMGT^®^ http://www.imgt.org, IMGT/mAb-DB) [60].

### 2.4. Synthesis and Expression of the Immunoglobulins (IG) or Antibodies

#### 2.4.1. IG Molecular Synthesis Characteristics

**Figure 6:**
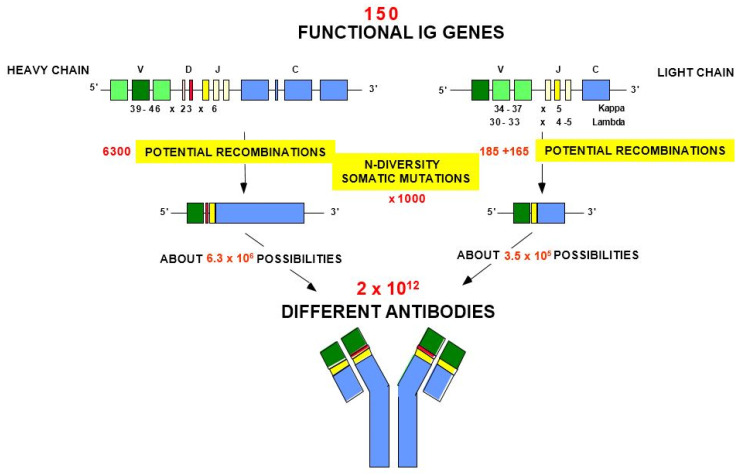
Molecular synthesis of the immunoglobulins (IG) or antibodies and origin of their diversity. The gene numbers are from the human (*Homo sapiens*) IGH, IGK and IGL loci. The molecular mechanisms creating the diversity of the antigen receptors of the adaptive immune responses include the combinatorial diversity (V-(D)-J rearrangements), the junctional diversity (including the N-diversity), the somatic hypermutations (SHM) and the pairing of the heavy and light chains. Altogether the mechanisms of diversity that occur at the DNA level in the B cell result in about 6.3 × 10^6^ and about 3.5 × 10^6^ possibilities of heavy and light chains, respectively, and the pairing of one heavy chain with one light chain (the antibody is made of two identical heavy and light chains) results into a potential repertoire of 2 × 10^12^ different antibodies. The two inter-H-L and the inter-H-H disulfide bridges which depend on IG or antibody class or subclass are not shown. Only the core gene regions are represented. V = V-REGION (in green), D = D-REGION, J = J-REGION, C = C-REGION (in blue). The gene regions involved in the H chain V-D-J and L chain V-J rearrangements are highlighted: V (in dark green), D (in red) and J (in yellow). (With permission from M-P. Lefranc and G. Lefranc, LIGM, Founders and Authors of IMGT^®^, the international ImMunoGeneTics information system^®^, http://www.imgt.org).

The variable domain of a heavy chain, designated as VH, is a V-D-J-REGION encoded by three rearranged genes (IGHV, IGHD and IGHJ) (Table 1). In humans, the genes encoding the heavy chains and the light chains, kappa and lambda, are located in the IGH, IGK and IGL loci on chromosome 14 (14q32.33), 2 (2p11.2) and 22 (22q11.2), respectively [2]. The synthesis of the IG heavy and light chains requires gene rearrangements, at the DNA level [122,123,124], in the IGH, IGK and IGL loci during the B cell differentiation in bone marrow (Figure 3). The synthesis of the chains of the antigen receptors, immunoglobulin (IG) or antibody [1,2], and T cell receptors (TR) [1,3], includes several molecular mechanisms that occur at the DNA level and are unique to the B and T cells (Figure 6): (a) combinatorial V-D-J or V-J rearrangements of the variable (V), diversity (D) and joining (J) genes (b) N-diversity resulting from the exonuclease trimming at the ends of the V, D, and J genes and the random addition of nucleotides at the V-(D)-J junctions before the gene ligation, and (c) later, during B cell differentiation, for the IG, somatic hypermutations (SHM) in the rearranged V-(D)-J genes. During the transcription in B or T cells, the rearranged V-(D)-J gene which codes the V domain is spliced to a C gene that codes the C region. Chronologically in B cells, the synthesis of the H-mu chains precedes that of the light chains.

#### 2.4.2. Synthesis of the H-Mu Chains: D-J and V-D-J Rearrangements in the IGH Locus

**Figure 7:**
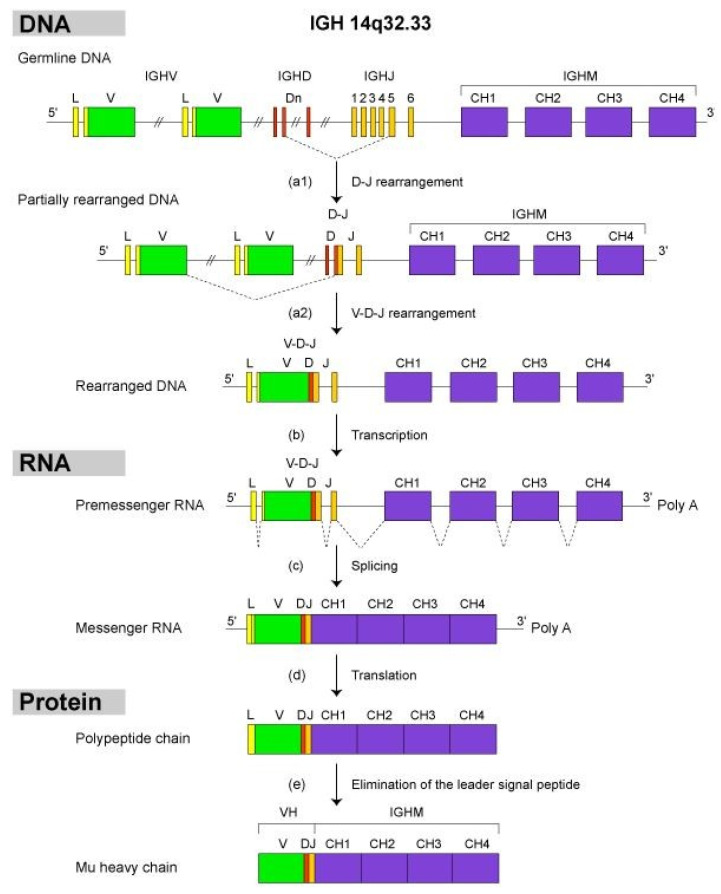
Synthesis of an IG H-mu chain [2]. (**a1**) At the DNA level, in a first step, one of the IGHD genes is joined to one of the IGHJ genes, with deletion of the intermediary DNA, to create a partially rearranged D-J gene. (**a2**) In a second step, one of the IGHV genes is joined to D-J, with deletion of the intermediary DNA, to generate a completely rearranged IGHV-D-J gene. (**b**) The rearranged IGHV-D-J gene is transcribed with the IGHM gene into a IGHV-D-J-M (or IGHV-D-J-Cmu) pre-messenger RNA. (**c**) The RNA sequences corresponding to the introns and to the non-used IGHJ genes are excised by splicing, and a mature messenger which comprises the spliced coding regions and the 5′ and 3′ untranslated sequences, is obtained. (**d**) The messenger RNA is translated into a polypeptide chain by the ribosomes. (**e**) The signal peptide is cleaved off by a peptidase following the entry of the polypeptide chain in the endoplasmic reticulum, and a mature H-mu chain is produced. In DNA and pre-messenger RNA, L for Leader corresponds L-PART1 and L-PART2, and in spliced messenger RNA to L-REGION [2]. (With permission from M-P. Lefranc and G. Lefranc, LIGM, Founders and Authors of IMGT^®^, the international ImMunoGeneTics information system^®^, http://www.imgt.org).

The IGH locus comprises variable (V), diversity (D), joining (J) and constant (C) genes. The variable domain of a heavy chain, or VH, is a V-D-J-REGION generated by the junction at the DNA level of three genes: a variable gene IGHV, a diversity gene IGHD and a joining gene IGHJ (Figure 7). The synthesis requires two successive rearrangements. First, one of the IGHD genes is joined to one of the IGHJ genes with deletion of the intermediary DNA as an excision loop, then one of the IGHV genes is joined to the partially rearranged D-J gene to generate a completely rearranged IGHV-D-J gene. This second rearrangement is also accompanied by the formation of an excision loop which is cleaved off. The rearranged IGHV-D-J gene is transcribed with the IGHM gene, the most 5′ IGHC gene in the locus, into a IGHV-D-J-M (or IGHV-D-J-Cmu) pre-messenger RNA. The IGHM gene encodes the four domains (CH1 to CH4) of the H-mu constant region. After splicing of the pre-messager RNA, translation of the messenger RNA, and elimination of the signal peptide by a peptidase in the endoplasmic reticulum, a mature H-mu chain is produced [2].

#### 2.4.3. Synthesis of the L-Kappa and L-Lambda Chains: V-J Rearrangements in the IGK and IGL Loci

**Figure 8:**
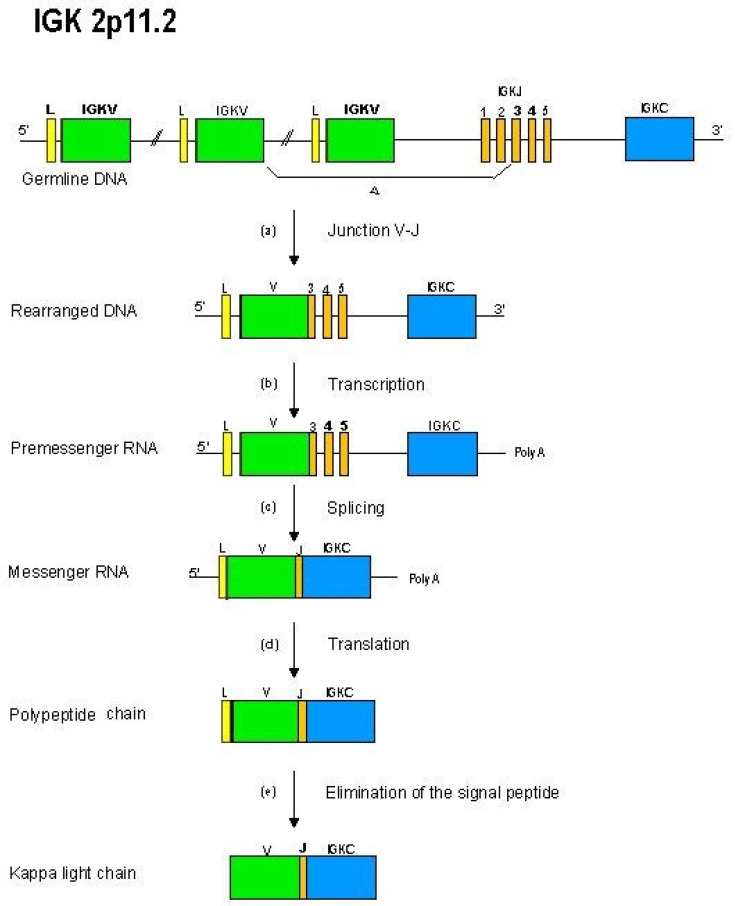
Synthesis of a L-kappa chain [2]. (**a**) At the DNA level, one of the IGKV gene is joined to one of the five IGKJ genes, with deletion of the intermediary DNA, to create a rearranged IGKV-J gene. (**b**) The rearranged IGKV-J sequence is transcribed with the IGKC gene into a IGKV-J-C pre-messenger RNA. (**c**) The RNA sequences corresponding to the introns and to the non-used IGKJ genes are excised by splicing, and a mature messenger which comprises the spliced coding regions, and the 5′ and 3′ untranslated sequences, is obtained. (**d**) The messenger RNA is translated into a polypeptide chain by the ribosomes. (**e**) The signal peptide is cleaved off by a peptidase following the entry of the polypeptide chain in the endoplasmic reticulum, and a mature L-kappa chain is produced. In DNA and pre-messenger RNA, L for Leader corresponds to L-PART1 and L-PART2, and in spliced messenger RNA to L-REGION [2]. (With permission from M-P. Lefranc and G. Lefranc, LIGM, Founders and Authors of IMGT^®^, the international ImMunoGeneTics information system^®^, http://www.imgt.org).

The kappa (IGK) locus and the lambda (IGL) locus comprise variable (V), joining (J) and constant (C) genes. The variable domain of a L-kappa or L-lambda chain, V-KAPPA or V-LAMBDA, is a V-J-REGION, generated by the junction, at the DNA level, of two genes: a variable and a joining genes, with deletion of the intermediary DNA to create a rearranged IGKV-J gene in the IGK locus (Figure 8), or a IGLV-J gene in the IGL locus.

The rearranged IGKV-J (or IGLV-J) gene is transcribed with the IGKC gene (or one of the IGLC genes) into a IGKV-J-C (or IGLV-J-C) pre-messenger RNA. The unique IGKC gene, or one of the functional IGLC genes, with their single exon, encodes the single domain of the constant region of the L-kappa or L-lambda chains, respectively. After splicing of the pre-messenger RNA, translation of the messenger RNA and elimination of the signal peptide from the polypeptide chain in the endoplasmic reticulum, a mature L-kappa (or L-lambda) chain is produced [2].

### 2.5. Origin of the Variable Domain Diversity of the Immunoglobulins

#### 2.5.1. Overview

The diversity of the variable domains of the immunoglobulin chains arises mainly from combinatorial diversity, V-J and V-D-J junctional diversity and somatic hypermutations [2]. In addition, within an immunoglobulin, the pairing of the variable domains of the heavy chain (VH) and of the light chain (VL, V-KAPPA or V-LAMBDA), to form the antigen recognition and binding site, creates an additional degree of diversity [2].

#### 2.5.2. Combinatorial Diversity

**Figure 9:**
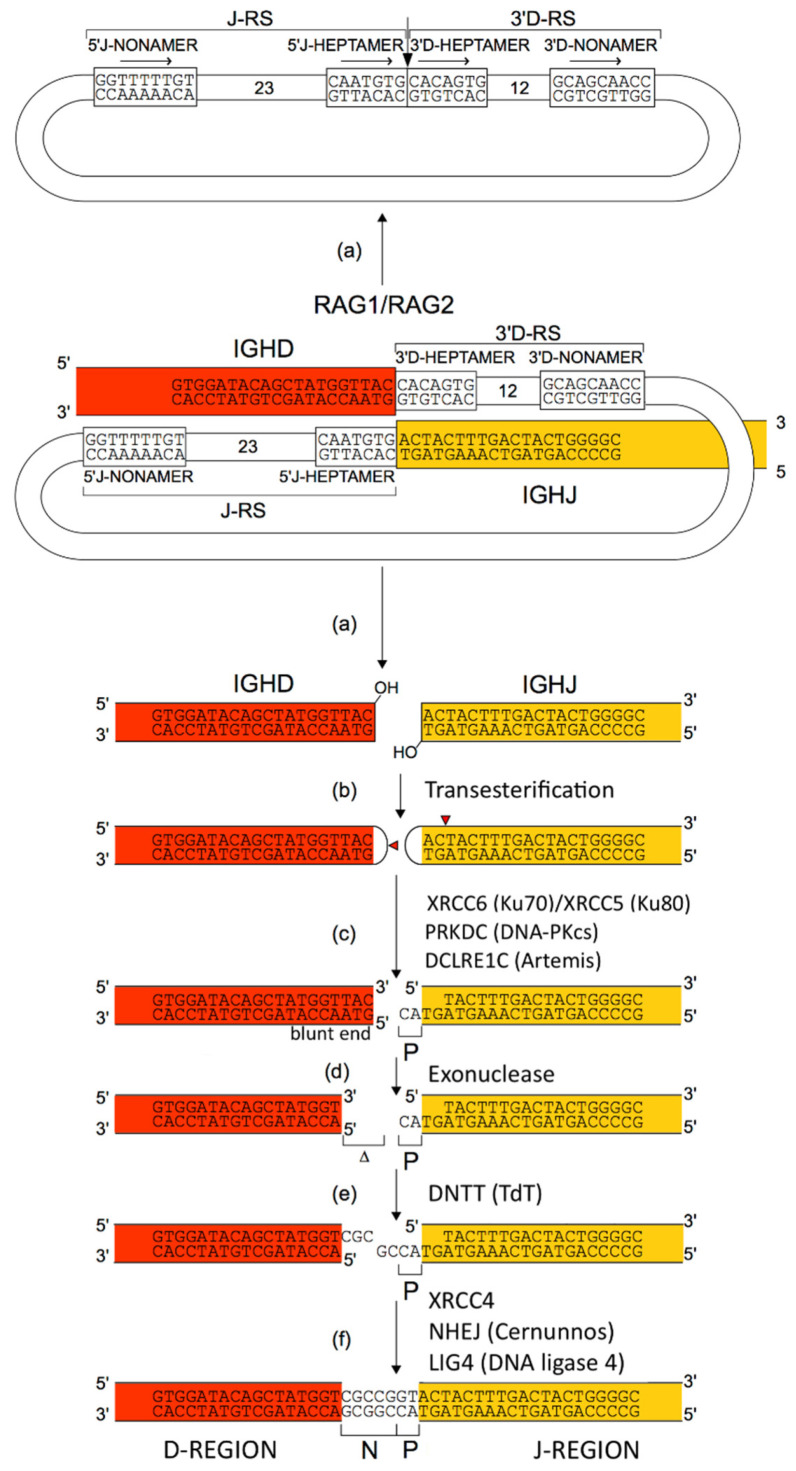
Molecular events generating the junction N-diversity, in a rearrangement between a D gene and a J gene in the IGH locus. (**a**) The enzymatic RAG1/RAG2 protein complex recognizes the recombination signal (RS) sites and cuts the germline DNA between the heptamer and the coding region, resulting in a coding joint (below) and a signal joint (top) which is eliminated as an excision circle. (**b**) A hairpin is formed at each coding end, here 3′D and 5′J ends, by transesterification. (c) The haipin is cut by the protein complex (X-ray repair cross complementing 6 (XRCC6, Ku70)/ X-ray repair cross complementing 5 (XRCC5, Ku80), protein kinase DNA-activated catalytic subunit (PRKDC, DNA-PKcs, DNA-PK), DNA cross-link repair 1C (DCLRE1C, Artemis). Depending on the position of the cut-off site, a blunt end or P nucleotides are obtained. (**d**) An exonuclease eliminates nucleotides at the coding ends. (**e**) DNA nucleotidylexotransferase (DNTT, TdT, terminal deoxynucleotidyl transferase) adds N nucleotides, preferably ‘g’ and ‘c’ at random without template. (**f**) Finally, the rearranged DNA is repaired and ends are ligated by the ligase complex (X-ray repair cross complementing 4 (XRCC4), non-homologous end joining factor 1 (NHEJ1, Cernunnos), LIG4 (DNA ligase 4, ligase IV DNA ATP-dependent). (With permission from M-P. Lefranc and G. Lefranc, LIGM, Founders and Authors of IMGT^®^, the international ImMunoGeneTics information system^®^, http://www.imgt.org).

The combinatorial diversity is created by the somatic V-D-J and V-J rearrangements. The somatic IGKV-J, IGLV-J and IGHV-D-J rearrangements require the presence of recombination signal (RS) sequences which are located in 3′ of the V genes, 5′ of the J genes, and on both sides of the D genes [2,125] (Figure 9). The RS comprise two highly preserved motifs, a palindromic heptamer and a nonamer rich in “a” or “t”, separated by a not conserved spacer of 12 ±1 or 23 ±1 nucleotides. They are recognized by the recombinase enzymes (recombination activating 1 (RAG1) and recombination activating 2 (RAG2)) [126,127]. Efficient rearrangements occur between RS of different lengths, that is one with a 12 ± 1 spacer, and another one with a 23 ± 1 spacer (12/23 joining rule) [128] (IMGT^®^ http://www.imgt.org, IMGT Repertoire (IG and TR) > 1. Locus and genes > 7. Gene tables > Recombination signals sequence logos; ibid: IMGT Education > Tutorials > Immunoglobulins and B cells > Molecular genetics of immunoglobulins). The potential repertoire resulting from the combinatorial diversity depends on the number of V, D and J genes, and on their functionality.

#### 2.5.3. Junctional Diversity

The junctional diversity is represented by the V-J junction diversity of the L-kappa chains which creates variability of the amino acid at position 115 (IMGT unique numbering) of the rearranged CDR3 [125,129], and by the N-diversity (N, for nucleotides) essentially observed at the V-D-J junctions of the IG heavy chains, and which represents the major source of the CDR3 diversity. In 1982 Alt and Baltimore proposed a model that explains the mechanism of N-diversity [130]. It results from the excision of nucleotides by an exonuclease at the ends of the V, D and J genes during rearrangement (3′ end of the V-REGION, 5′ end of the J-REGION and/or both ends of the D-REGION), followed by the addition of nucleotides randomly via the DNA nucleotidylexotransferase (DNTT, terminaldeoxynucleotidyltransferase TdT) [131]. This addition of nucleotides preferentially involves ‘g’ nucleotides and is template independent. If the ends of the coding regions are intact (no deletion due to the exonuclease), P-nucleotides may be observed adjacent to these coding regions [132]. P-nucleotides are short sequences of 1 to 3 nucleotides palindromic (inverted repeat) to the intact coding end (3′ end of the V-REGION, 5′ end of the J-REGION and/or both ends of the D-REGION. P-nucleotides result from the dissymmetric opening of the hairpin formed at the extremities of the coding regions during V-J or V-D-J rearrangements [133].

#### 2.5.4. Somatic Hypermutations 

Somatic hypermutations (SHM) appear during the B cell maturation in the germinal centers of the secondary lymphoid organs (spleen and lymph nodes) (Figure 3). They specifically affect the IG rearranged V-J and V-D-J genes during the antigen dependent stages of differentiation and represent a major mechanism for the generation of diversity of the variable domains of antibodies. This process of somatic hypermutation involves the introduction, to a high rate of point mutations in rearranged VH and VL (V-KAPPA or V-LAMBDA) sequences [134,135,136,137,138,139]. Other genes expressed in B cells are not changed by the mechanisms of somatic hypermutation. SHM occurs at a frequency estimated 10^−3^ per base in a B cell which is roughly 10^6^ times more as frequent as the rate of spontaneous mutations in other cells. Somatic hypermutation shares with class-switch recombination (CSR) common mechanisms: they are both initiated by activation induced cytidine deaminase (AICDA, AID) which necessitates transcription to target single-strand DNA. AICDA induces the deamination of cytosine (c) to uracil (u). The replication system transforms the uridine in thymidine leading to a c > t transition (and g > a transition on the opposite strand). In SHM, the uracil is removed by uracil DNA glycosylase (UNG), and this abasic site is further processed by either the DNA base excision repair (BER) pathway or the DNA mismatch repair (MMR) systems. The DNA lesions are repaired by error-prone DNA polymerases, leading to nucleotide transitions and transversions [139].

### 2.6. Expression of the Heavy Chains and IG Classes

#### 2.6.1. Coexpression of the Membrane H-Mu and H-Delta Chains

During its differentiation, an immature B cell becomes a naive mature B cell which expresses simultaneously membrane IgM and IgD classes (Figure 3). The VH domains of the H-mu and H-delta chains are identical, and are encoded by the same rearranged V-D-J-REGION. The mechanism of expression of the H-delta isotype differs from the expression of other isotypes in that its expression depends on a splicing mechanism and not on the class switching mechanism like the other isotypes. IgD is coexpressed with IgM on the surface of naive mature B cells (only case where two different IG classes are expressed by the same cell).

The IGHM and IGHD genes are located nearby in the IGH locus. B cells which express IgM and IgD produce two types of RNA premessengers of the IGH-V-D-J-gene, the first ones ending after the IGHM gene, and the second ones, containing IGHM and IGHD, ending after the IGHD gene and long of about 20 kilobases (kb) (the distance separating IGHM and IGHD being 6 kb in the human IGH locus). The RNA premessengers ending after the IGHM gene are spliced to produce mature IGHV-D-J-Cmu mRNA, translated into membrane H-mu chains. The RNA premessengers ending after the IGHD gene undergo splicing which removes the IGHM gene and produces mature IGHV-D-J-Cdelta mRNA, translated into membrane H-delta chains [140,141,142]. It is not excluded that this long premessager is also used to produce H-mu chains. 

#### 2.6.2. Expression of H-Gamma, H-Epsilon and H-Alpha Chains: Class Switch Recombination

**Figure 10:**
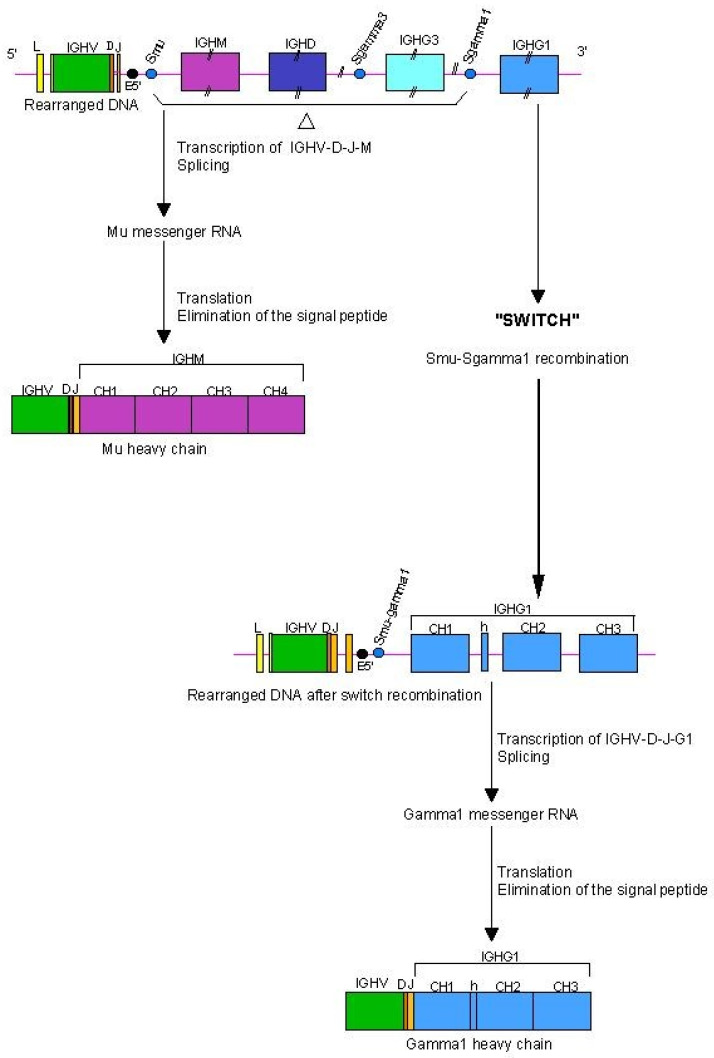
Class switch IgM-IgG1: Smu-Sgamma1 recombination [2]. In a B cell which expresses IgM on the cell surface, a productive rearranged IGHV-D-J gene on one chromosome 14 is transcribed with the IGHM gene. Before the switch (S) recombination, all the IGHC genes are present in the IGH locus. During the switch recombination, a novel DNA rearrangement occurs in the IGH locus, between the Switch mu (Smu) sequence and another S sequence located in 5′ of a more downstream IGHC gene (for example, Sgamma1 upstream of IGHG1). This leads to the deletion of the intermediary DNA and to the loss of the IGHC genes located between the two S sequences which recombine. The enhancer (E) located between the most 3′ IGHJ and Smu is retained during the switch recombination [2]. (With permission from M-P. Lefranc and G. Lefranc, LIGM, Founders and Authors of IMGT^®^, the international ImMunoGeneTics information system^®^, http://www.imgt.org).

The mature B cells which enter the lymph nodes express the IgM and IgD classes (Figure 3). After antigenic stimulation, activated B cells proliferate and can differentiate to produce other isotypes: the class switch recombination (CSR) occurs in the lymph nodes when B cells mature as a result of B and T cell cooperation. The activated B lymphocyte by its major histocompatibiliy MH2 proteins comes into contact with a T lymphocyte CD4 +. Recognition of B cell peptide/MH2 (p/MH2) by the T cell receptor (TR) leads to the T cell activation (expression of cytokines and of CD40LG on the T cell surface). The interaction between the CD40 (TNFRSF5) constitutively expressed on the B cell and its ligand CD40LG expressed on the surface of activated T cell leads to the expression of cytokin receptors on B cells, which in presence of the interleukins secreted by the T cell, provide the signal for the B cell to switch from IgM and IgD to IgG (IgG1, IgG2, IgG3 or IgG4) or IgA (IgA1 or IgA2) or IgE classes. This switch results in a change of the constant region of the heavy chain, while maintaining the expression of the same antibody specificity. In the switch recombination, the rearranged IGHV-D-J gene, previously associated with the IGHM gene in a H-mu messenger RNA of a B cell expressing IgM, is brought into the proximity of one of the other IGHC genes, downstream (more in 3′) in the locus, for example IGHG1 [2] (Figure 10). 

The switch recombination occurs between switch sequences located at about 2 kb in 5′ of each IGHC gene (except IGHD) (Figure 10). The switch sequences, of about 2 kb, are composed of 20–80 nucleotide motifs repeated in tandem. These motifs contain short ‘gggct’ and ‘gagct’ repeats and, near the recombination site, ‘tggg’ or ‘tgag’. The class switch involves the recombination of the Smu sequence with the S sequence of another IGHC gene, for example with a Sgamma sequence in the case of a switching from IgM to IgG, resulting in the deletion of the IGHC genes located between Smu and the Sgamma of the IGHG gene used (Figure 10). This occurs by the formation of excision loops which are cleaved off [143,144,145,146,147]. For example, in the case of switching from IgM to IgG1 (Figure 10), the IGHM, IGHD and IGHG3 genes are deleted [2]. 

At the molecular level, CSR shares a number of features with SHM. It necesitates transcription of the C regions which starts from a small exon (called I) located upstream of each switch region, resulting in a sterile J-C transcript which does not encode any protein. As mentioned above, CSR is also initiated by AICDA. Transcription allows the separation of both DNA strands which are then targeted by deamination of multiple ‘c’ nucleotides by AICDA. This is followed by generation of single-strand breaks by the BER system (single-strand break by the apurinic/apyrimidic endonuclease 1 (APE1) at a UNG abasic site), which may be converted into double-strand breaks by the MMR proteins. After loop excision of the intervening sequence, fusion of the switch regions is thought to be mediated by the non-homologous end-joining (NHEJ) system [148,149].

#### 2.6.3. Expression of H-Delta Chains from IgM^−^ IgD^+^ Cells

Only a minority of normal plasma cells and rare B cell malignancies express exclusively IgD (IgM^−^IgD^+^ B cells). The low frequency has been explained by the lack of a recognizable switch sequence between IGHM (Cmu) and IGHD (Cdelta). However, a region, designated as sigma delta, contains a relatively high content of pentameric repeats with an extremely “g-rich” area and appears to function as a vestigial switch recombination site leading to the expression of delta chains in germinal center B cells and plasma cells [150,151,152].

#### 2.6.4. Expression of Membrane and Secreted Immunoglobulins

**Figure 11:**
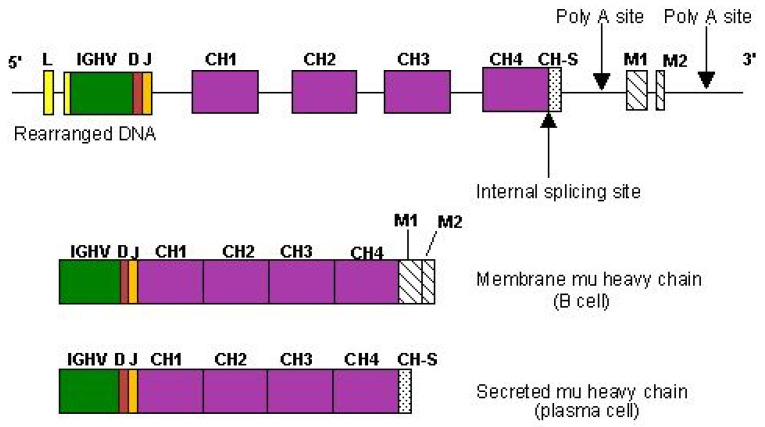
Synthesis of a membrane and of a secreted H-mu chain [2]. (With permission from M-P. Lefranc and G. Lefranc, LIGM, Founders and Authors of IMGT^®^, the international ImMunoGeneTics information system^®^, http://www.imgt.org).

Heavy chains of membrane and secreted immunoglobulins differ in their C-terminal region. The heavy chains of the membrane IG on the B cell surface have a hydrophobic C-terminal end which holds them anchored in the plasma membrane, whereas the heavy chains of the plasma cell secreted IG have an hydrophilic end [153]. Expression of membrane and secreted IG results from an alternative splicing of the heavy chain transcripts (Figure 11).

The C-terminal region of the membrane H-mu chain is encoded by two small exons, M1 and M2 located at about 2 kb in 3′ of the CH4 exon [154], M1 encodes 39 amino acids, whereas M2 only encodes two amino acids (Figure 11). These 41 amino acids represent the anchor region of the membrane H-mu chain which comprises an extracellular region (CO) of 13 amino acids between the CH4 domain and the membrane, a hydrophobic transmembrane region (TM) of 27 amino acids and a short cytoplasmic region (CY) of one amino acid. The C-terminal region of the secreted H-mu chain comprises 20 amino acids encoded by the 3′ end of the CH4 exon (designated as CHS) [2].

For the synthesis of a membrane H-mu chain, it is the poly A site located in 3′of the M2 exon, and the splicing site located in the CH4 exon, at the 5′ limit of CHS which are used (Figure 11). This splicing deletes the CHS sequence and its stop codon, as well as the sequence between CH4 and M1, and between M1 and M2. For the synthesis of a secreted H-mu chain, it is the poly A site located 103 bp from the 3′ end of the CH4 exon, and the stop codon at the 3′ end of CH4 which are used (Figure 11). One cell can therefore present the two H-mu RNA precursors and the relative expression of a H-mu chain, membrane or secreted, depends on a control in the selection of the polyA site used [155]. The organization of the 3′ region of the IGHD gene differs due to the presence of a small independent CHS exon located at 1.9 kb in 3′ of the CH3 exon, and which encodes the nine last amino acids of the secreted H-delta chains. The M1 and M2 exons, located at 0.8 kb and 1.1 kb in 3′ of CHS, respectively, encode the TM and CY, M1 encodes 53 amino acids whereas M2 encodes two amino acids. The expression of the membrane and secreted H-delta chain depends on the selection of the poly A used: poly A in 3′ of the IGHD exon M2, for the synthesis of the membrane H-delta chain, or poly A in 3′ of CHS for the synthesis of the secreted H-delta chain [156].

The expression of the membrane and secreted H-gamma, H-alpha and H-epsilon chains follows the same mechanisms as those described for the H-mu chain, the CHS being part of a domain (CH3 or CH4 depending on the IGHC gene).

### 2.7. Regulation of the Rearrangements and Chain Expression 

#### 2.7.1. Allelic and Isotypic Exclusion. Rearrangement Chronology

The B cells, and the plasma cells which derive from them, display: - allelic exclusion: in most cases, the only productive genes are either those of the paternal chromosome, or those of the maternal chromosome, but usually not the two together (functional haploidy); - isotypic exclusion: a single type of light chain, L-kappa or L-lambda, and usually a single type de heavy chain belonging to a given subclass, are synthesised.

Molecular analysis has shown that the excluded allele is usually either not rearranged, or unproductively rearranged (IGK locus in B cells synthesising a lambda chain) [157,158].

During the B cell differentiation, the IGH locus on one chromosome 14 undergoes first a D-J, then a V-D-J rearrangement. A productive rearrangement allows the synthesis of a H-mu chain in the cytoplasm of the pre-B cells.The H-mu chain is expressed at the surface of the pre-B cells in association with a lambda-like chain (IGLL1) and a V-pre-B (VPREB1) chain, which constitute together the pre-B cell receptor [159,160] (Figure 3) [2].

**Figure 12:**
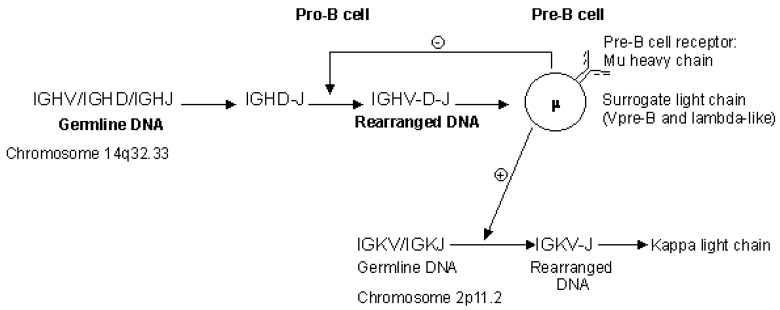
Regulation of the immunoglobulin gene rearrangements in the pre-B cell [2]. The expression of the pre-B cell receptor gives the signal which inhibits further IGHV-D-J rearrangements on chromosome 14, and the signal which starts the IGKV-J rearrangements on chromosome 2 [2]. (With permission from M-P. Lefranc and G. Lefranc, LIGM, Founders and Authors of IMGT^®^, the international ImMunoGeneTics information system^®^, http://www.imgt.org).

The expression of the surface IgM at a later stage requires the synthesis of L-kappa or L-lambda chains, that is a productive V-J rearrangement of the IGK or IGL loci. It is the expression of the pre-B cell receptor at the surface of a pre-B cell which gives the signal which inhibits further IGHV-D-J rearrangements on chromosome 14 (Figure 12), and the signal which starts the light chain V-J rearrangements. Chronologically the V-J rearrangements of the IGK locus usually precede those of the IGL locus [157,158]. A chromosome 2 will be rearranged first. If the resulting rearrangement is productive, L-kappa chains will be synthesized, which will allow the expression of IgM, the other chromosome 2 and both chromosomes 22 remaining germline. If the first rearrangement is unproductive, the other chromosome 2, then the chromosome 22 will be rearranged until a productive rearrangement allows the synthesis of a light chain. Thus, in a B cell which produces antibodies, generally only one chromosome 14 is productively rearranged (expressing a productive H-mu chain) whereas only one chromosome 2 or 22 is productively rearranged (expressing a productive L-kappa or L-lambda chain). IG genes on the other chromosomes are either germline, or rearranged but unproductive, or deleted.

#### 2.7.2. Regulation of the IG Gene Expression: Enhancers

In order to synthesize complete IG heavy and light chains, the rearranged IGHV-D-J and IGKV-J (or IGLV-J) genes are transcribed with the IGHM gene and with the IGKC (or one IGLC) genes, respectively. The transcription level, low in the first B cell development stages, becomes very high in the plasma cells which result from the clonal proliferation [161]. The V genes possess a promoter sequence in 5′ of L-PART1 (exon encoding the first part of the leader peptide) [162] and can be transcribed before they rearrange. These germline transcripts correspond to an opening, and therefore to better accessibility of the chromatin before the rearrangements. However, that transcription remains low. The C genes can also be transcribed from promoter sequences located upstream of the J genes but again the level of transcription is low and the transcripts are degraded in the nucleus [163].

Murine and human IG transcription enhancers are the first enhancers described in the DNA of eucaryote cells [164,165,166,167]. By different approaches several groups have simultaneously shown that a DNA segment located between the most 3′ IGHJ and the switch Smu sequence is not only able to increase the transcription, but also possesses the properties of the enhancers previously described in the viruses. These enhancers (i) activate the transcription whatever their orientation and their position (in 5′ or 3′) relative to the gene promoter, (ii) only activate the promoters located in *cis*, that is on the same chromosome, and (iii) activate in vitro genes others than those to which they are normally associated in vivo and increase their transcription, even when located at a distance of several kb [168]. 

The presence of an enhancer in the *Homo sapiens* IG loci has been demonstrated between the most 3′ IGHJ and the IGHM gene [169], and between the most 3′ IGKJ and the IGKC gene [170,171]. When a IGHV-D-J or IGKV-J rearrangement occur, the promoter sequence in 5′ of the IGHV or IGKV genes is not modified, but this promoter is now closer to the enhancer sequences located in 3′ of the IGHJ or IGKJ genes. By decreasing the distance between the V gene promoter and the enhancer, the IGHV-D-J and IGKV-J rearrangements allow the interaction of factors binding to these sequences, and consequently an increased trancription of the IGHV-D-J-Cmu and IGKV-J-Ckappa transcripts. During the switch recombination, the IGH enhancer, being localized at more than 1 kb upstream from the Smu sequence, is retained in the locus and can therefore be used for the expression of all the heavy chain classes and subclasses. A second enhancer has been described in 3′ of the IGKC gene [172]. Two 3′ enhancers have been characterized in the *Homo sapiens* IGH locus, one downstream of IGHA1, and another one downstream of IGHA2, within 25 kb of each gene, respectively [173]. These enhancers were duplicated along with part of the IGH locus [114,174,175,176], which occurred between about 30 and 60 million years ago. An enhancer has also been localized in the *Homo sapiens* IGL locus in 3′ of IGLC7, the most 3′ IGLC gene [177]. This enhancer consists of three modules located 6, 9.8 and 11.7 kb downstream of IGLC7 [178].

### 2.8. Structural and Biological Properties of the Secreted Immunoglobulins

#### 2.8.1. IgM

IgM represents about 10% of total serum immunoglobulins in human and is largely confined to the intravascular pool. It exists almost exclusively as a polymeric form (pentamer) made of five monomer units associated with the polypeptide designated as J (joining) chain [179]. IgM is the predominant antibody produced early in the immune response. Pentameric IgM is decavalent with small antigens but only pentavalent with larger antigens, presumably due to steric hindrance (Table 2). A disulfide bridge connects the H-mu chains between CH2 and CH3. Disulfide bridges between the CH3 and the tailpieces of the different monomers are involved in the IgM polymerization. A single J chain is present per IG. This amounts to 1.5% of pentameric IgM. The conserved features of the J chain (16 kDa) is the presence of a N-glycosylation site Asn-Ile-Ser and of eight cysteines. Six of the cysteines form three intradisulfide bridges, and two are linked to the penultimate cysteine of two H-mu chains. The five ‘Fc’ (or paired CH3 and CH4 of each monomer) are arranged into a planar pentamer (Fc5) (IMGT/3Dstructure-DB PDB 2rcj) [180]. Electron microscope studies have revealed that uncomplexed IgM has a planar and ”star” conformation with the 10 Fab arms protruding out from the Fc5. On binding to an antigenic surface, the F(ab’)2 dislocate out of the plane of the central Fc5 disc, giving a “staple” or “crab-like” (“table-like”) conformation [104]. In this latter conformation, IgM is a very efficient activator of the classical complement pathway. C1q interacts directly with the CH3 domain of the IgM.

#### 2.8.2. IgD

IgD represents less than 1% of total serum immunolobulins. IgD has a long and extended hinge region of 58 amino acids encoded by two exons, which allows great flexibility in the relative position of the two Fab arms. The hinge N-terminus half of 34 AA encoded by the first exon is heavily O-glycosylated (four to seven oligosaccharides). The hinge C-terminus half of 24 AA encoded by the second exon is rich in charged amino acids (2 Arg, 6 Lys, 9 Glu) and is very susceptible to proteolytic attack, whch makes serum IgD unstable (Table 2). 

#### 2.8.3. IgG

IgG is the major antibody class in normal human serum forming about 70% of the total immunoglobulins. IgG is a monomer and is evenly distributed between intravascular and extravascular pools. IgG is the predominant antibody of the secondary immune responses. There are four subclasses in humans.

The effector molecules binding IgG Fc are C1q, the Fcgamma receptors (FcγR) present on the surface of many cells of the immune system, the neonatal Fc receptor (FCGRT, FcRn) which transports maternal IgG to the foetus, and the bacterial Fc receptors, protein A and protein G, which are believed to mask the bacteria through immobilized immunoglobulins on their surface. 

C1q interacts directly with the CH2 domain of IgG. Binding to monomeric IgG is weak, but when several IgG bind to, and effectively aggregate at an antigenic surface, two or more C1q heads may bind simultaneously leading to tighter binding and activation of the complement cascade. There are marked differences in ability to activate complement: IgG1 and IgG3 activate well, IgG2 less well, IgG4 not at all (Table 2). 

Three classes of human FcγR have been described: FcγRI (CD64) are receptors with high affinity for IgG Fc and possess three C-like extracellular domains, FcγRII (CD32) and FcγRIII (CD16) are receptors with lower affinity and possess two C-like domains [181]. FcγRI, FcγRII and FcγRIII on macrophages and Natural Killer (NK) cells mediate antibody-dependent cell-mediated cytotoxicity (ADCC) and phagocytosis, whilst FcγRI, FcγRII and possibly FcγRIII on neutrophils are able to trigger release of activated oxygen species. The cellular responses also comprise endocytosis, enhanced antigen presentation and regulation of the antibody production, depending on the particular FcγR isoform and the type of cell [181]. FcγRI (CD64) displays high affinity for monomeric human IgG1 and IgG3, whilst the affinity for human IgG4 is about 10-fold lower and human IgG2 does not bind. The human FcγRII (CD32) binds IgG1 and IgG3. IgG4 does not bind, whilst the binding of IgG2 is controlled by an allotypic determinant in certain forms of the receptor. FcγRI and FcγRII appear to recognize overlapping but non-identical sites in the lower hinge region of IgG. 

The crystal structures of the Fc of human IgG1 in complex with *Staphylococcus aureus* protein A [182], or in complex with streptococcal protein G [183], and that of Fc of rat in complex with FCGRT (FcRn) [184] revealed binding sites at the interface between the CH2 and CH3 Fc domains. The crystal structure of the human IgG1 Fc fragment-FcγRIII complex shows that FcγRIII binds to the two CH2 domains and lower hinge of the Fc [185].

#### 2.8.4. IgA

##### IgA1 and IgA2

IgA forms about 15–20% of total serum immunoglobulins where it occurs as a monomer. IgA is the predominant immunoglobulin in seromucous secretions such as saliva, tracheobronchial secretions, colostrum, milk and genitourinary secretions, where it is found in a dimeric form known as secretory IgA (sIgA). There are two subclasses of IgA, with IgA1 being the predominant (80–90%) subclass in serum. In contrast to serum IgA, secretory IgA shows roughly equal proportions of the two subclasses. The two IgA subclasses differ in the hinge: IgA1 has an effective structural hinge of 19 amino acids containing eight potential glycosylation sites, whereas IgA2 has a short structural hinge of six amino acids including five prolines which, by its nature, is resistant to proteolysis. A further peculiarity of IgA2 is that for most molecules (allotype A2m1), the light chain is disulfide bridged, not to the heavy chain but to the light chain of the other Fab unit. The CH2 domain of both IgA suclasses has seven cysteines. Two are involved in the usual intradomain disulfide bridge, another two in a second intradomain bridge and one is thought to be free, possibly for interaction with secretory component. The remaining two form inter-H disulfide bridges. There is a further intradomain disulfide linkage in CH1 in addition to the conserved domain disulfide. 

##### Secretory IgA

The dimer involves J chain (16 kDa) and another polypeptide known as secretory component (SC) (70 kDa). Selective transport of polymeric IgA through epithelial cells depends on the incorporation of the J chain into the polymers. Two of the J chain cysteines are linked to the penultimate cysteine of the alpha chains. The J chain, which was identified initially in human IgA [186] amounts to 4% of dimeric human IgA. The SC, unlike IG and J chain which are produced by plasma cells, is synthesized in epithelial cells. With extra segments to attach it to the epithelial cell membrane, SC serves as a receptor for polymeric IG (poly-IG) containing J chain, i.e., IgA (or IgM). After endocytosis and transport, cleavage of the poly-IG/poly-IG receptor complex releases poly-IG (poly-IG with the J-chain) associated with SC. This process is particularly important for secretory IgA release. The poly-IG receptor is composed, in its poly-IG binding portion (i.e., SC) of five highly conserved C-like domains of approximately 100 amino acids. SC (70 kDa) probably interacts non-covalently with the Fc and J chain and forms a single disulfide bridge to one of the monomers of dimeric IgA.

##### IgA Effector Function 

IgA can activate the alternative complement pathway and bind to specific FcαR. FcαR is present on monocytes, macrophages, neutrophils and eosinophils and can mediate ADCC, phagocytosis and degranulation. FcαR has two extracellular C-like domains and spans the membrane once. FcαR binds at a site in the IgA CH2 domain. 

#### 2.8.5. IgE

IgE [187,188,189], though a trace IG in serum, is found bound to receptors, specific for the IgE Fc, on the cell surface of mast cells and basophils in all individuals. IgE is involved in protection against helminthic parasites [190] but is most commonly associated with atopic allergies. The ability of IgE-Fc to undergo conformational changes is critical for IgE function [191]. IgE binds to two principal receptors, FCER1 (FcεRI, tetrameric IgE Fc receptor I), the “high affinity” receptor for the IgE Fc, on the surface of mast cells and basophils, and FCER2 (IgE Fc receptor II, FcεRII, CD23), the “low affinity“ receptor for IgE Fc, a Ca^2+^ -dependent C-type lectin and a B cell specific antigen [192]. 

FCER1 is tetrameric and consists of an alpha chain (FCER1A, IgE Fc binding site) chain, a beta chan (FCER1B, which amplifies the signal), and two disulfide linked gamma chains (FCER1G, where the downstream signal initiates). FCERI is expressed on tissue mast cells, blood basophils, airway epithelial and smooth muscle cells and intestinal epithelial cells [193,194]. Aggregation of the receptors by binding of multivalent antigens, such as pollen, to prebound IgE results in cell degranulation and release of pre-formed mediators of inflammation causing an allergic response and an immediate hypersensitivity response that, if intense, can cause anaphylactic shock and even death. The FCERIA binding site on IgE involves CH3 (next to the interface between CH2 and CH3). The crystal structure of the human IgE Fc and its high-affinity FCERIA reveals that the receptor binds one Fc asymmetrically. The CH3 of each chain of the Fc is bound to two different sites of the C-like domain [D2] of FCERIA (IMGT/3Dstructure-DB, PDB code: 1f6a) [195].The IgE Fc is highly flexible adopting an acutely bent conformation when unbound (IMGT/3Dstructure-DB: PDB code: 2y7q) [196], partially bent conformation in a complex with omalizumab Fab (IMGT/3Dstructure-DB, PDB code: 5g64) [197], fully extended in a complex with aεFab (IMGT/3Dstructure-DB, PDB code: 4j4p) [198] and with the 8D6 Fab (IMGT/3Dstructure-DB, PDB code: 6eyo) [199]. 

FCER2 (FcεRII, CD23), the low-affinity receptor for IgE is present on monocytes, B cells, T cells, gut and airway epithelial cells, plays a role in cytotoxicity against parasites such as schistosomes. FCER2 has essential roles in B cell growth and differentiation, and the regulation of IgE production [200,201,202,203]. It also exists as a soluble secreted form [204], then functioning as a potent mitogenic growth factor. The interaction between IgE and FCER2 appears to require the presence of the IgE CH2, CH3 and CH4 domains, the latter serving to promote the dimerization of the two epsilon chains, necessary for receptor binding. Crystal structure of IgE bound to its B cell FCER2 reveals a mechanism of reciprocal allosteric inhibition with the high affinity receptor FCER1 (IMGT/3Dsructure-DB, PDB code: 4gko) [205].

Antibodies classically bind antigens via their complementarity determining regions, but an alternative mode of interaction involving V-domain framework regions has been observed for some B cell “superantigens”. The crystal structure of an antibody from an allergic individual, bindng to the grass pollen allergen *Phl p* 7 has shown that both modes of interaction were employed simultaneously with binding of two antigen molecules (IMGT/3Dstructure-DB, PDB code: 5otj) [206].

## 3. Immunoglobulin Genes: IMGT^®^ Gene and Allele Nomenclature

### 3.1. IMGT^®^ Standardized Genes and Alleles (Classification)

#### 3.1.1. IG and TR Genes and Concepts of Classification: Birth of IMGT^®^ and Immunoinformatics

**Figure 13:**
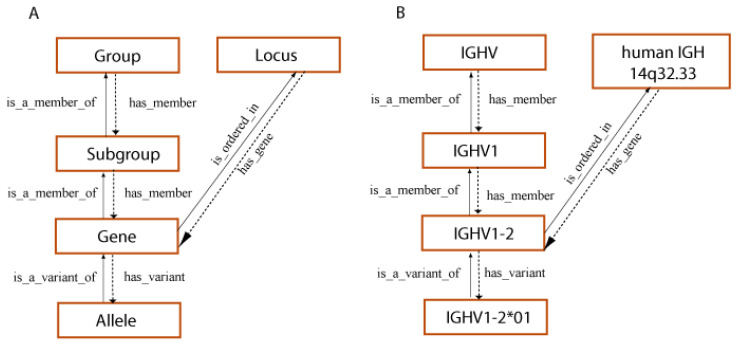
Concepts of classification for IMGT gene and allele nomenclature (CLASSIFICATION axiom) [37,38]. (**A**) Hierarchy of the concepts of classification and their relations. The definition of the reciprocal relations between concepts can be read, from one concept to the other, either ascending the hierarchy (solid arrows) or descending the hierarchy (dotted arrows). (**B**) Examples of concept instances for each concept of classification. The concept instances are associated to an instance of the “Taxon” concept, and more precisely for the “Gene” and “Allele” concepts to an instance of the “Species” concept (here, *Homo sapiens*) [1,2,3,4,5,37,38,84,85]. The “Locus” concept is a concept of localization (LOCALIZATION axiom) [28]. It is shown with the reciprocal relations to the “Gene” concept. (With permission from M-P. Lefranc and G. Lefranc, LIGM, Founders and Authors of IMGT^®^, the international ImMunoGeneTics information system^®^, http://www.imgt.org).

The creation of IMGT^®^ in 1989 by Marie-Paule Lefranc (LIGM, UM, CNRS), during the 10th Human Genome Mapping Workshop (HGM10, New Haven, CT, USA, 11–17 June 1989) gave birth to immunoinformatics, a new science at the interface between immunogenetics and bioinformatics [1]. Indeed, for the first time, immunoglobulin (IG) or antibody and T cell receptor (TR) variable (V), diversity (D), joining (J), and constant (C) genes were officially recognized as “genes” as well as were the conventional genes, with the entry of all genes of the *Homo sapiens* TRG locus in the HGM database [207,208]. This major breakthrough allowed IG and TR genes and alleles of the complex and highly diversified adaptive immune responses to be managed in genomic databases and tools. IMGT^®^ gene and allele names are based on the concepts of classification of ‘Group’, ‘Subgroup’, ‘Gene’ and ‘Allele’ [37,38,84,85]. ‘Group’ allows to classify a set of genes which belong to the same multigene family, within the same species or between different species. For example, there are 10 groups for the IG of higher vertebrates: IGHV, IGHD, IGHJ, IGHC, IGKV, IGKJ, IGKC, IGLV, IGLJ, IGLC. ‘Subgroup’ allows to identify a subset of genes which belong to the same group, and which, in a given species, share at least 75% identity at the nucleotide level, e.g., *Homo sapiens* IGHV1 subgroup. Subgroups, genes and alleles are always associated to a species name (84,85). An allele is a polymorphic variant of a gene, which is characterized by the mutations of its sequence at the nucleotide level, identified in its core sequence (V-REGION, D-REGION, J-REGION, C-REGION) and compared to the gene allele reference sequence, designated as allele *01. For example, *Homo sapiens* IGHV1-2*01 is the allele *01 of the *Homo sapiens* IGHV1-2 gene that belongs to the *Homo sapiens* IGHV1 subgroup which itself belongs to the IGHV group (84,85) (Figure 13). For the IGH locus, the constant genes are designated by the letter (and eventually number) corresponding to the encoded isotypes (IGHM, IGHD, IGHG3…), instead of using the letter C. IG and TR genes and alleles are not italicized in publications.

The IMGT IG and TR gene names [1,2,3,4,5] were approved by the Human Genome Organisation (HUGO) Nomenclature Committee (HGNC) in 1999 and were endorsed by the WHO-IUIS Nomenclature Subcommittee for IG and TR [84,85]. The IMGT IG and TR gene names, with the allele *01 of the IMGT reference sequences, have been provided since 1999, via HGNC, to NCBI (first ‘LocusLink’, then ‘Entrez Gene’, now superseded by ‘Gene’) [2,3,4,5]. 

IMGT human IG and TR gene names have been integrated in the CLASSIFICATION axiom [37,38] of IMGT-ONTOLOGY [29] (IMGT^®^ http://www.imgt.org, IMGT Index > IMGT-ONTOLOGY), on the NCBO BioPortal and, on the same site, in the HUGO ontology and in the National Cancer Institute (NCI) Metathesaurus. Since 2006, IMGT gene and allele names have been used for the description of the therapeutic mAb and FPIA from the WHO-INN programme [86,87]. Amino acid sequences of the IMGT human IG and TR constant genes (e.g., *Homo sapiens* IGHM, IGHG1, IGHG2) were provided to UniProt in 2008, and those of the IMGT human IG variable genes with their IMGT gene definition (e.g., P23083, *Homo sapiens* IGHV1-2, Immunoglobulin heavy variable 1-2), in 2016. There are reciprocal direct links between the gene entries of IMGT/GENE-DB [56], the IMGT^®^ gene database, and HGNC, NCBI Gene, Ensembl, Vega and UniProt. 

#### 3.1.2. *Homo sapiens* IG Genes and Concepts of Identification and Description

To date (July 25, 2020), four hundred sixty-five IG genes have been identified in *Homo sapiens*, 389 of them in Major Locus and 76 in orphon sets [56] (Table 5). Genes of the major loci participate to the IG chain synthesis whereas those of the orphon sets do not.

**Figure 14:**
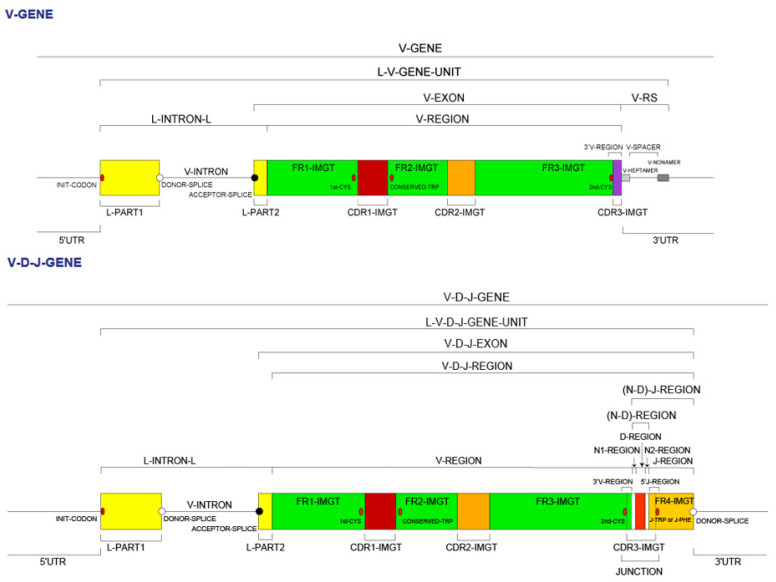
Description of a V-GENE and of a V-D-J-GENE (DESCRIPTION axiom) [34,35]. IMGT standardized labels [32,33,34], generated from the concepts of description, are in capital letters (no plural), e.g., V-REGION, JUNCTION, CDR-IMGT [32], FR-IMGT [33]. Forty labels (27 for V-GENE and 33 for V-D-J-GENE of which 20 are shared) are necessary and sufficient for a complete description. The labels for the description of genomic rearranged genes in IMGT/LIGM-DB [54,55] are L-V-J-GENE and L-V-D-J-GENE. The labels “GENE-UNIT” have been created to bridge the DESCRIPTION and CLASSIFICATION axioms. Indeed they refer to genomic entities, in germline (for V, D and J) or undefined (for C) configuration, entered in IMGT/GENE-DB [56]. Exceptionally they may refer to rearranged entities (as the L-V-D-J-GENE-UNIT, shown here) when the corresponding germline genes are not yet known. (With permission from M-P. Lefranc and G. Lefranc, LIGM, Founders and Authors of IMGT^®^, the international ImMunoGeneTics information system^®^, http://www.imgt.org).

**Figure 15:**
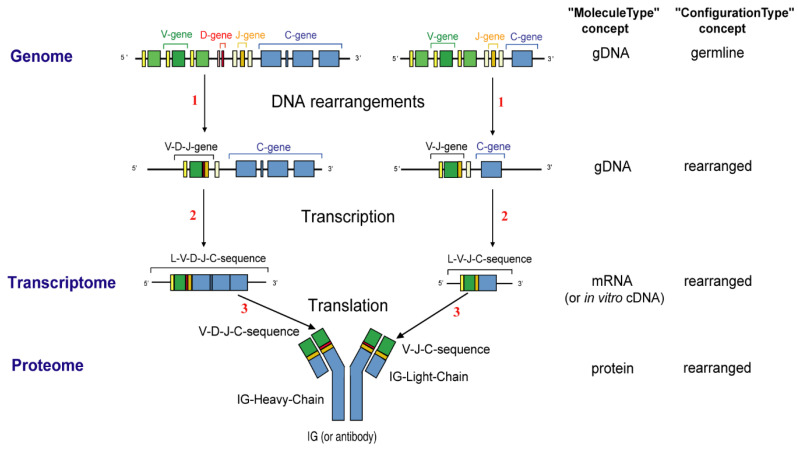
Molecular entity types of the IG synthesis (IDENTIFICATION axiom) [16,30,31] in jawed vertebrates (*Gnathostomata*). (1) DNA rearrangements (is_rearranged_into). (2) Transcription (is_transcribed_into). (3) Translation (is_translated_into). IMGT standardised keywords, generated from the concepts of identification, identify the entity types based on the MoleculeType and and the ConfigurationType. The MoleculeType is gDNA, mRNA, in vitro cDNA or protein. The ConfigurationType is undefined (conventional and C-gene), germline (V-gene, D-gene and J-gene) or rearranged. The rearranged entities include V-D-J-gene (label L-V-D-J-GENE) and V-J-gene (label L-V-J-GENE) (gDNA), L-V-D-J-C-sequence and L-V-J-C-sequence (cDNA), V-D-J-C-chain and V-J-C-chain (protein) [30]. The functionality of undefined and germline entities is functional (F), open reading frame (ORF) or pseudogene (P) [30]. The functionality of rearranged entities is productive or unproductive [30] (Table 6). L= L-REGION (shown in ‘Transcriptome’) (light yellow), V = V-REGION (in green), D = D-REGION, J = J-REGION, C = C-REGION (in blue). The gene core regions involved in the V-D-J (heavy chain) and V-J (light chain) rearrangements are highlighted: V (in dark green), D (in red) and J (in orange yellow). (With permission from M-P. Lefranc and G. Lefranc, LIGM, Founders and Authors of IMGT^®^, the international ImMunoGeneTics information system^®^, http://www.imgt.org).

Given the complexity of the IG synthesis which generates a huge diversity of sequences, thirty-two molecular entity types have been defined. They are identified with IMGT standardized keywords [30], based on the “GeneType”, “ConfigurationType”, and “MoleculeType” which define them (IDENTIFICATION axiom [30,31]). The ten most classical IG and TR entity types are shown in Table 6. To each molecular entity type corresponds a molecular entity prototype. These prototypes are described with IMGT standardized labels [34] (DESCRIPTION axiom [34,35]). The V-GENE and V-D-J-GENE are shown as examples (Figure 14). Other prototypes are available on the IMGT^®^ web site (IMGT^®^ http://www.imgt.org, IMGT Scientific chart > 1. Sequence and 3D structure identification and description > IMGT prototypes table). The ten molecular entity types are reported in the IG synthesis in Figure 15, bridging genes, sequences and proteins. 

### 3.2. Homo sapiens IGH Locus and Genes

#### 3.2.1. Organization of the *Homo sapiens* IGH Locus

**Figure 16:**
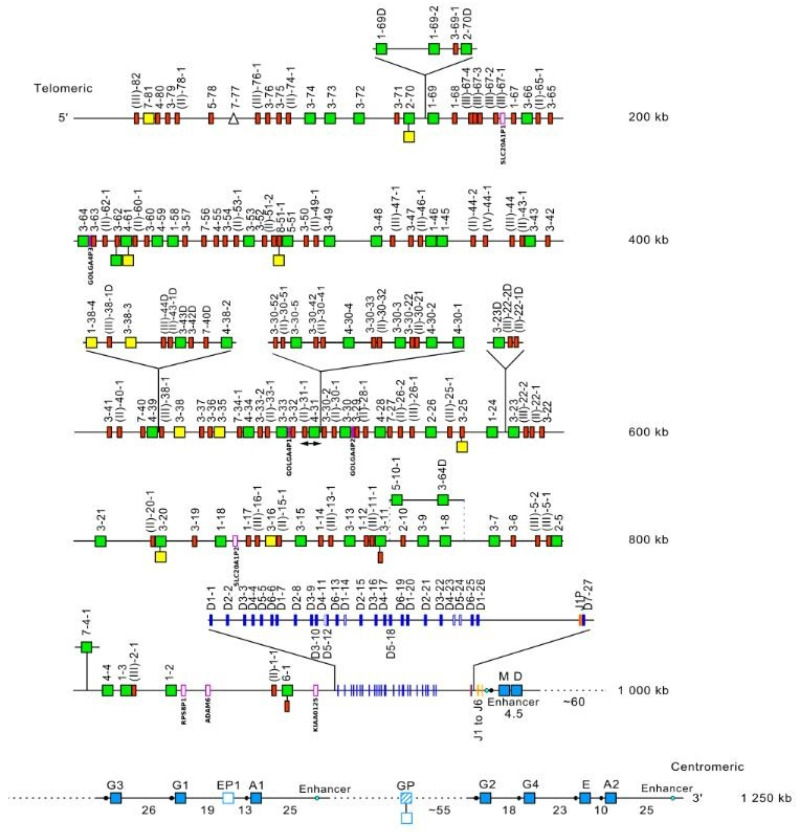
Representation of the human IGH locus at 14q32.33 [2]. The boxes representing the genes are not to scale. Exons are not shown. Switch sequences are represented by a filled circle upstream of the IGHC genes. Pseudogenes which could not be assigned to subgroups with functional genes are designated by a Roman numeral between parentheses, corresponding to the clans, followed by a hyphen, and a number for the localization from 3′ to 5′ in the locus [2]. (With permission from M-P. Lefranc and G. Lefranc, LIGM, Founders and Authors of IMGT^®^, the international ImMunoGeneTics information system^®^, http://www.imgt.org).

The *Homo sapiens* IGH locus is located on chromosome 14 [209], at the telomeric extremity of the long arm [210,211], at band 14q32.33 [2]. The orientation of the locus has been determined by the analysis of translocations, involving the IGH locus, in leukemia and lymphoma. The *Homo sapiens* IGH locus spans 1250 kb [2] (Figure 16). It consists of 123 to 129 IGHV genes [212,213,214,215,216,217,218] depending on the haplotypes, 27 IGHD genes, belonging to seven subgroups [219,220,221,222,223], nine IGHJ genes [223,224] and, in the most frequent haplotype, 11 IGHC genes [114,119,120,154,174,175,225,226,227,228,229,230,231,232,233,234,235,236]. Eighty-two to 88 IGHV genes belong to seven subgroups [2,237], whereas 41 pseudogenes, which are too divergent to be assigned to subgroups, have ben assigned to the clans. 

#### 3.2.2. IGHC Multigene Deletions and Gene Order, IGHC and IGHV Copy Number Variation (CNV) Haplotypes

**Figure 17:**
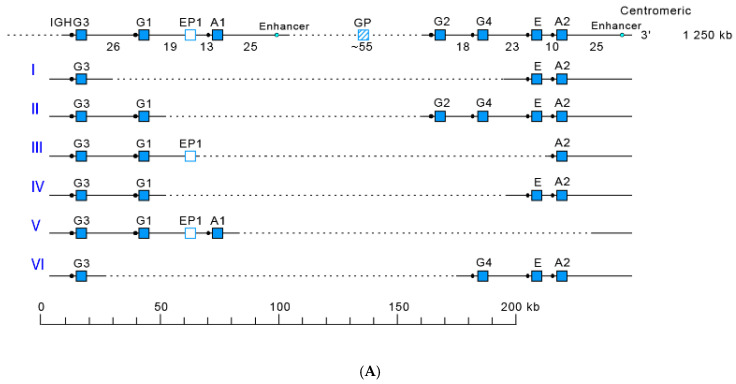

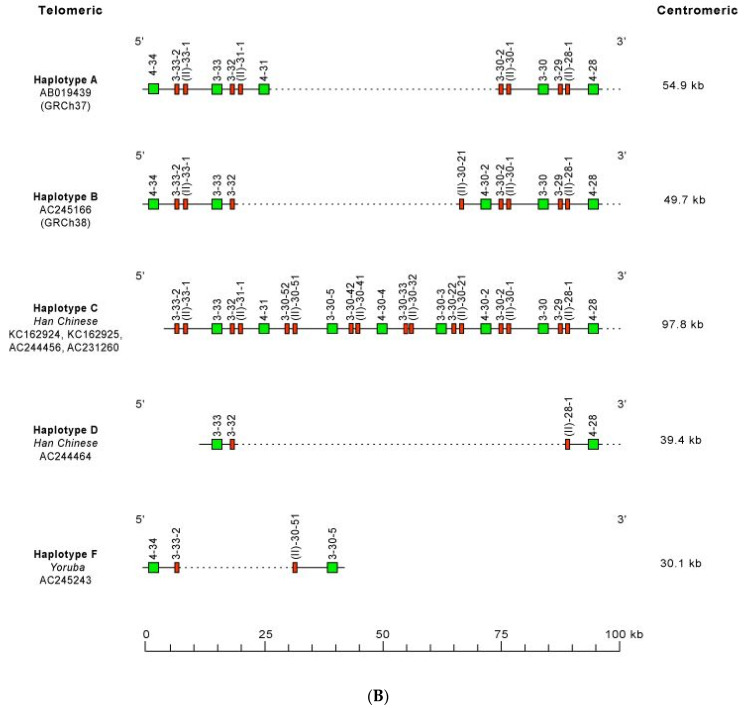
Multigene deletions and copy number variations (CNV) in the *Homo sapiens* IGH locus on chromosome 14 (14q32.33). (**A**). IGHC-CNV deletions, either identical or different, on both chromosomes, designed I to VI according to the chronogical order in which they were found. Deletion I, first identified by the absence of the Gm1 allotypes in a 70-year old healthy Tunisian woman (TAK3), homozygous for that deletion [113,114] allowed the ordering of the *Homo sapiens* IGHC genes in the IGH locus [174,175]. Deletions I and II [113,114,115], found in healthy individuals from consanguinesous families, involve highly homologous spots of recombination [176], as also described in an healthy individual (T17) homozygous for deletion III and lacking IgA1, IgG2, IgG4 and IgE [116]. (**B**). Polymorphisms by insertion/deletion between IGHV4-34 and IGHV4-28 (haplotypes A to F). The distance between IGHV4-34 and IGHV4-28 (sequence length shown by the regular line) is indicated in kilobases (kb) for each haplotype. Haplotype A is from GRCh37 and corresponds to the main line of IMGT Locus Representation [2]. Haplotype B is from GRCh38 and corresponds to BAC clone sequences [247] from the CHORI-17 BAC library. Dotted lines indicate missing genes compared to the haplotype C. (With permission from M-P. Lefranc and G. Lefranc, LIGM, Founders and Authors of IMGT^®^,the international ImMunoGeneTics information system^®^, http://www.imgt.org).

IGHC haplotypes have been identified in *Homo sapiens*, which correspond to IGHC copy number variations (IGHC CNV) with absence of the corresponding IG classes and subclasses in healthy indiduals having IGHC deletions on both chromosomes (either homozygous for a same deletion, or heterozygous for two different deletions). These IGHC CNV deletions are designed I to VI according to the chronogical order in which they were found (Figure 17). 

The first two identified deletions, deletion I (del G1-EP1-A1-GP-G2-G4) [113,114] and deletion II (del EP1-A1-GP) [115] allowed ordering of the *Homo sapiens* IGHC genes by determinng the relative positions of two cosmids [174,175]. Deletion III (del A1-GP-G2-G4-E) [116,238] includes the IGHE gene and corresponds to the complete absence of the IgA1, IgG2, IgG4 subclasses and of the IgE class as demonstrated by the gene deletion molecular analysis in the healthy and homozygous individual T17 [116]. The mechanism of these IGHC multigene deletions [113,114,115,116,239,240], several of them found in conguineous families [241,242]. involves, as demonstrated for one of them, highly homologous hot spots of recombination [176]. CNV with IGHC multigene duplication or triplication were detected by Southern analysis and pulsed field gel electrophoresis (PFGE) [117,243,244,245,246]. 

The IGHV cluster comprises several CNV reported in Figure 17B. As an example, the genome assembly from GRCh38 from the hydatidiform mole CHORI-17 BAC library corresponds to a new haplotype (haplotype B) in the highly polymorphic region by insertion/deletion between IGHV4-34 and IGHV4-28 [247] (IMGT^®^ http://www.imgt.org, IMGT Repertoire (IG and TR) > 2. Locus representations > IGH Locus representation: Human (*Homo sapiens*) Polymorphism by insertion/deletion between IGHV4-34 and IGHV4-28 (haplotypes A to F)). In addition, eight CNV-containing haplotypes were identified from a panel of nine diploid genomes of diverse ethnic origin [247]. These polymorphisms confirm the diversity of genomic IGH alleles and/or CNV polymorphisms identified in extensive studies of different populations, as restriction fragment length polymorphism (RFLP) alleles [117,248,249,250,251]

#### 3.2.3. IGH Orphons

Thirty-five IGH genes have been found outside the main locus in other chromosomal localizations. These genes designated as orphons cannot contribute in the synthesis of the immunoglobulin chains, even if they have an ORF. Nine IGHV orphons and 10 IGHD orphons have been described on chromosome 15 (15q11.2), and 16 IGHV orphons on chromosom 16 (16p11.2) [218]. In addition one IGHC processed gene, IGHEP2 (so far, the only processed IG gene described in the major loci) is localized on chromosome 9 (9p24.2-p24.1) [252] (IMGT^®^ http://www.imgt.org, IMGT Repertoire (IG and TR) > 1. Locus and genes > 7. Gene tables > IGHV > Human > Gene table: Human *(Homo sapiens)* IGHV orphons; ibid: IGHD; ibid: IGHC).

#### 3.2.4. Potential *Homo sapiens* IGH Genomic Repertoire

The potential genomic IGH repertoire per haploid genome comprises 38 to 46 functional IGHV genes belonging to six or seven subgroups depending on the haplotypes [2,237], 23 IGHD, 6 IGHJ and, in the most frequent haplotype, nine IGHC genes (Table 7). The total number of human IGH genes per haploid genome in the major locus is 170-176 of which 76-84 genes are functional.

The potential repertoire of the *Homo sapiens* IGH V-CLUSTER is shown in Table 7A and that of the IGH D-J-C-CLUSTER in Table 7B. The IGHV subgroups (Table 7A) are listed per subgroup and inside each subgroup, in an ascending numerical order. Only the IGHV subgroups with at least one functional allele are shown, excepted for the IGHV8 subgroup recently assigned with an ORF. The CDR-IMGT lengths (in number of AA or codons) are shown between square brackets, with lengths of the CDR1-IMGT, CDR2-IMGT and CDR3-IMGT separated by dots. The allele functionality is indicated by F: functional, (F): when the accession number refers to rearranged genomic DNA or cDNA and the corresponding germline gene has not yet been isolated, ORF: open reading frame, P: pseudogene. In the column ‘Nb of alleles P’, the number of pseudogenes with V-REGION in-frame and the number of pseudogenes with frameshift(s) are shown separated by a dot, between parentheses. Copy number variations (CNV) are numbered from 5′ to 3′ in the locus (Figure 16), with ‘i’ for an insertion, ‘d’ for a deletion, ‘e’ for an exchange.The IGHD, IGHJ and IGHC genes of the D-J-C-CLUSTER (Table 7B) are listed according to the gene order. All the IGHD genes have at least one in-frame reading frame without stop-codons, however four genes are assigned to ORF owing to an unusual 5′D-HEPTAMER. Only the six functional IGHJ genes are shown. The IGHEP1 (4 P) and the IGHGP (2 ORF, 1P) are listed owing to the structural organization of the IGHC genes in two duplicated clusters. Gene order is according to the IMGT Locus gene order (IMGT^®^ http://www.imgt.org, IMGT Repertoire (IG and TR) > 1. Locus and Genes > 3. Locus descriptions > Locus gene order > IGH).

### 3.3. Homo sapiens IGK Locus and Genes

#### 3.3.1. Organization of the *Homo sapiens* IGK Locus

**Figure 18:**
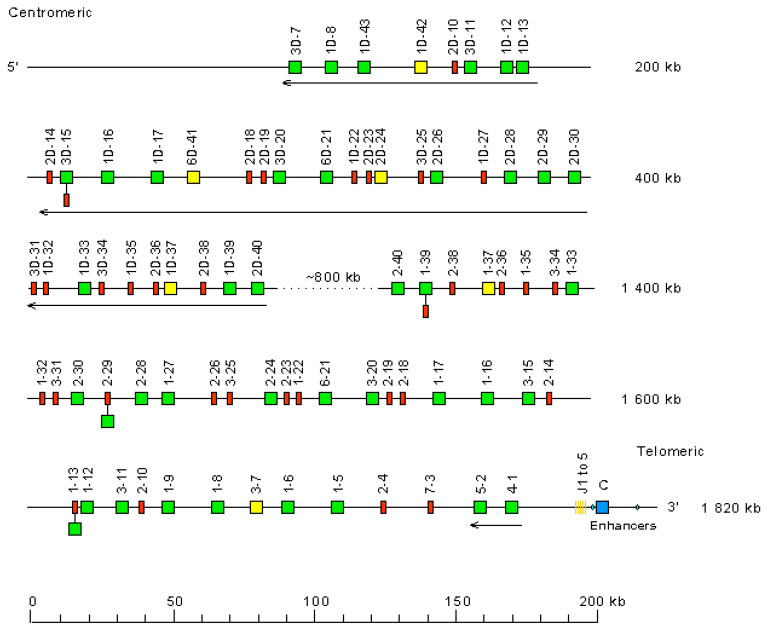
Representation of the human IGK locus at 2p12 [2]. The boxes representing the genes are not to scale. Exons are not shown. The IGKV genes of the proximal V-CLUSTER are designated by a number for the subgroup, followed by a hyphen and a number for the localization from 3′ to 5′ in the locus. The IGKV genes of the distal duplicated V-CLUSTER are designated by the same numbers as the corresponding genes in the proximal V-CLUSTER, with the letter D added. Arrows show the IGKV genes polarity of which is opposite to that of the J-C-CLUSTER [2]. (With permission from M-P. Lefranc and G. Lefranc, LIGM, Founders and Authors of IMGT^®^, the international ImMunoGeneTics information system^®^, http://www.imgt.org).

The *Homo sapiens* IGK locus is located on chromosome 2 [253], on the short arm [254], at band at 2p11.2 [2]. The orientation of the locus has been determined by the analysis of translocations, involving the IGK locus, in leukemia and lymphoma. The *Homo sapiens* IGK locus spans 1820 kb [2] (Figure 18). It consists of 76 IGKV genes belonging to seven subgroups [237,255,256,257,258,259,260,261], five IGKJ genes [261,262,263] and a unique IGKC gene [263]. The 76 IGKV genes are organized in two clusters separared by 800 kb [2]. The IGKV distal cluster in 5′ of the locus and in the most centromeric position) spans 400 kb and comprises 36 genes. The IGKV proximal cluster (in 3′ of the locus, closer to IGKC, aand in the most telomeric position) spans 600 kb and comprises 40 genes. 

The potential genomic IGK repertoire per haploid genome comprises 31-35 functional IGKV genes belonging to five subgroups, the five IGKJ genes and the unique IGKC gene [2,264]. One rare IGKV haplotype has been described which contains only the proximal cluster. This haplotype comprises the 40 proximal IGKV genes belonging to seven subgroups, of which 17–19 are functional and belong to five subgroups [2]. If both the proximal and distal IGKV clusters are present, the total number of human IGK genes per haploid genome is 82 of which 37 to 41 are functional. If only the proximal IGKV cluster is present, the total number of genes per haploid genome is 46 of which 23-25 genes are functional [2].

#### 3.3.2. IGK Orphons

Twenty-five IGKV orphons have been identified and sequenced: two on the short arm of chromosome two but outside of the major IGK locus, 12 on the long arm of chromosome 2, five on chromosome 22, one on chromosome 1, one on chromosome 15, and four outside of chromosome 2 [2,261] (IMGT^®^ http://www.imgt.org, IMGT Repertoire (IG and TR) > 1. Locus and genes > 7. Gene tables > IGKV > Human > Gene table: Human (*Homo sapiens*) IGKV orphons).

#### 3.3.3. Potential *Homo sapiens* IGK Genomic Repertoire

The potential repertoire of the *Homo sapiens* IGK V-CLUSTER is shown in Table 8A and that of the IGK J-C-CLUSTER in Table 8B. The IGKV subgroups (Table 8A) are listed per subgroup and inside each subgroup in an ascending numerical order, first the proximal cluster, then the distal cluster. Only the IGKV subgroups with at least one functional allele are shown. The CDR-IMGT lengths (in number of AA or codons) are shown between square brackets, with lengths of the CDR1-IMGT, CDR2-IMGT and CDR3-IMGT separated by dots. The allele functionality is indicated by F: functional, [F]: when the accession number refers to genomic DNA, but not known as being germline or rearranged, ORF: open reading frame, P: pseudogene. In the column ‘Nb of alleles P’, the number of pseudogenes with V-REGION in-frame and the number of pseudogenes with frameshift(s) are shown separated by a dot, between parentheses. Seven novel IGKV alleles were characterized [265] following the sequencing of the IGK locus from the CH17 hydatidiform mole BAC library. Gene order is according to the IMGT Locus gene order (IMGT^®^ http://www.imgt.org, IMGT Repertoire (IG and TR) > 1. Locus and Genes > 3. Locus descriptions > Locus gene order > IGK).

#### 3.3.4. *Homo sapiens* IGKC Allotypes (Km Alleles)

*Homo sapiens* IGKC allotypes are designated as Km (for ‘kappa marker’) (previously Inv) [83] The three Km allotypes Km1, Km2 and Km3 defined three Km alleles Km3 (or Km-1, Km-2 serologically), Km1,2 and Km1 which correspond to IGKC amino acid changes at position 45.1 and 101, according to the IMGT unique numbering for C-DOMAIN [42]. Km3. has Ala A45.1 and Val V101 and corresponds to four IGKC alleles (¨*01,*02;*03 and *05), Km1,2 has Ala A45.1 and Leu L101 (IGKC*04 allele) and Km1 has Val V45.1 and Leu L101 (IGKC*06) allele [83] (IMGT^®^ http://www.imgt.org, IMGT Repertoire (IG and TR) > 2. Proteins and alleles 4. Allotypes; ibid. 2. Alignments of alleles > IGKC > Mammalia > human (*Homo sapiens*)).

### 3.4. Homo sapiens IGL Locus and Genes

#### 3.4.1. Organization of the *Homo sapiens* IGL Locus 

**Figure 19:**
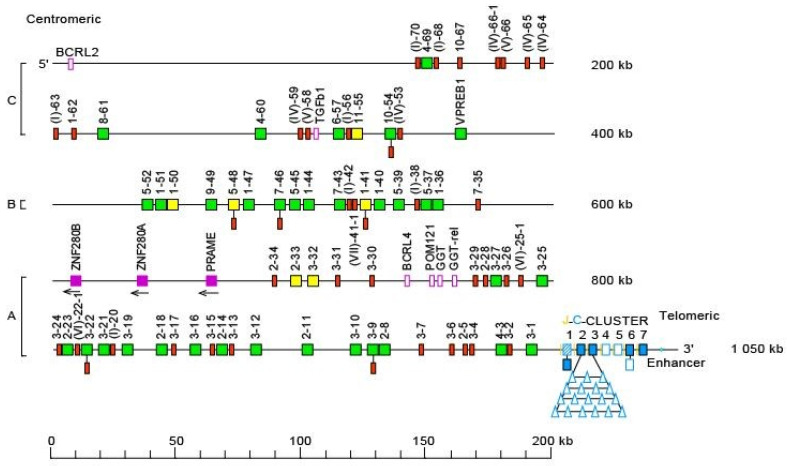
Representation of the human IGL locus at 22q11.2 [2]. The boxes representing the genes are not to scale. Exons are not shown. (**A**–**C**) refer to three distinct V-CLUSTER based on the IGLV gene subgroup content [271]. Pseudogenes which could not be assigned to subgroups with functional genes are designated by a Roman numeral between parentheses, corresponding to the clans, followed by a hyphen, and a number for the localization from 3′ to 5′ in the locus. (With permission from M-P. Lefranc and G. Lefranc, LIGM, Founders and Authors of IMGT^®^, the international ImMunoGeneTics information system^®^, http://www.imgt.org).

The *Homo sapiens* IGL locus is located on chromosome 22 [266], on the long arm, at band 22q11.2 [2], The human IGL locus spans 1050 kb [2] (Figure 19). The orientation of the locus has been determined by the analysis of translocations, involving the IGL locus, in leukemia and lymphoma [267]. Sequencing of the long arm of chromosome 22 showed that it encompasses about 35 megabases of DNA and that the IGL locus is localized at six megabases from the centromere [268]. Although the correlation between DNA seqences and chromosomal bands was not yet been made, the localization of the IGL locus could be refined at 22q11.2. The human IGL locus consists of 73-74 IGLV genes [237,269,270,271,272,273], localized on 900 kb, seven to 11 IGLJ and seven to 11 IGLC genes depending on the haplotypes, each IGLC gene being preceded by one IGLJ gene [274,275,276,277]. Fifty-six-57 genes belong to 11 subgroups, whereas 17 pseudogenes which are too divergent to be assigned to subgroups, have been assigned to the clans. The potential genomic IGL repertoire per haploid genome comprises 29-33 functional IGLV gnes in the 7-IGL gene haplotype [2,278,279]. One, two, three or four additional IGLC genes, each one probably preceded by one IGLJ, have been shown to characterize IGLC haplotypes with eight, nine, 10 or 11 genes [280,281], but these genes have not yet been sequenced. The total number of human IGL genes per haploid genome is 87–96 of which 37–43 are functional [2]. 

#### 3.4.2. IGL Orphons

Six orphons have been identified, four (two IGLV, two IGLC) on chromosome 22q outside of the major IGL locus and two IGLV on chromosome 8 [282]. A processed gene, IGLJ-C/OR18, has been found on chromosome 18 [283].

#### 3.4.3. Potential *Homo sapiens* IGL Genomic Repertoire

The potential repertoire of the *Homo sapiens* IGL V-CLUSTER in shown in Table 9A and that of the IGL J-C-CLUSTER in Table 9B. The IGLV subgroups (Table 9A) are listed per subgroup and inside each subgroup, in an ascending numerical order. Only the IGLV subgroups with at least one functional allele are shown. The CDR-IMGT lengths (in number of AA or codons) are shown between square brackets, with lengths of the CDR1-IMGT, CDR2-IMGT and CDR3-IMGT separated by dots. The allele functionality is indicated by F: functional, ORF: open reading frame, P: pseudogene. In the column ‘Nb of alleles P’, the number of pseudogenes with V-REGION in-frame and the number of pseudogenes with frameshift(s) are shown separated by a dot, between parentheses. IGLV alleles increase the diversity of the lambda light chain repertoire in the human population [284,285,286,287]. Four novel IGLV alleles and one IGLC allele were characterized [265] following the sequencing of the IGL locus from the CH17 hydatidiform mole BAC library. Limited CNV polymorphism by insertion and/or deletion seems to indicate that the V-CLUSTER of the human IGL locus has undergone less evolutionary shuffling that the human IGH or IGL loci [284]. However, CNV with a variable number of additional J-C cassettes, from one to four, have been identified by Southern blot analysis. These additional J-C cassettes are localized between the J2-C2 and J3-C3 cassettes. Gene order is according to the IMGT Locus gene order (IMGT^®^ http://www.imgt.org, IMGT Repertoire (IG and TR) > 1. Locus and Genes > 3. Locus descriptions > Locus gene order > IGL).

#### 3.4.4. *Homo sapiens* IGL Isotypes

The *Homo sapiens* IGL C-REGION, encoded by 4-5 IGLC genes in the 7-IGLC gene haplotype, belong to four serological isotypic forms. These lambda isotypes differ by limited amino acid changes which produce the Oz, Kern (Ke) and Mcg serological markers [2,276,279] (IMGT^®^ http://www.imgt.org, IMGT Repertoire (IG and TR) > 2. Proteins and alleles > 5. Isotypes > IGLC > Mammalia: human (*Homo sapiens*) IGLC). Mcg^+^ proteins have Asn N1 and Thr T3 (positions according to the IMGT unique numbering for C-DOMAIN [42]) whereas Mcg^−^ proteins have Ala 1 and Ser 3. Position 82 initially described as characteristic of the Mcg marker is not involved. Ke^+^ proteins have Gly G45 whereas Ke^−^ proteins have Ser S45. Oz^+^ proteins have Lys K100 whereas Oz^−^ proteins have Arg R100 [276,279].

## 4. Immunoglobulin Structures: IMGT Unique Numbering and IMGT^®^ Collier de Perles

### 4.1. IMGT Unique Numbering and IMGT Colliers de Perles for V-DOMAIN (NUMEROTATION)

#### 4.1.1. V Domain Definition and Main Characteristics

The V domain includes the V-DOMAIN of the IG and of the TR, which corresponds to the V-J REGION or V-D-J-REGION encoded by V-(D)-J rearrangements [2,3], and the V-LIKE-DOMAIN of the IgSF other than IG and TR. The V domain description of any receptor, any chain and any species is based on the IMGT unique numbering for V domain (V-DOMAIN and V-LIKE-DOMAIN) [39,40,41,44,45,46]. 

A V domain comprises about 100 amino acids and is made of nine antiparallel beta strands (A, B, C, C′, C″, D, E, F and G) linked by beta turns (AB, CC′, C″D, DE and EF) and three loops (BC, C′C″ and FG), forming a sandwich of two sheets [ABED] [GFCC’C”] [39,40,41,44,45,46]. The sheets are closely packed against each other through hydrophobic interactions giving a hydrophobic core, and joined together by a disulfide bridge between a first highly conserved cysteine (1st-CYS) in the B strand (in the first sheet) and a second equally conserved cysteine (2nd-CYS) in the F strand (in the second sheet) [39,40,41,44,45,46]. 

#### 4.1.2. V-DOMAIN IMGT Colliers de Perles

**Figure 20:**
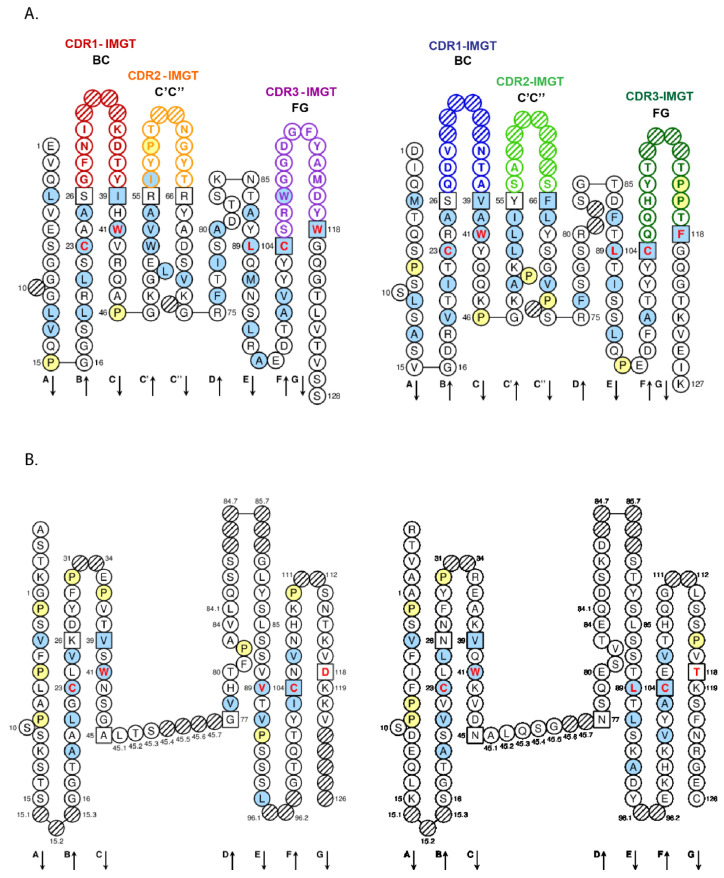
IMGT Collier de Perles of IG V-DOMAIN and C-DOMAIN on one layer [40,41,42]. (**A**) Trastuzumab VH (on the left) and V-KAPPA (on the right). The CDR1-IMGT, CDR2-IMGT and CDR3-IMGT (corresponding to the BC, C’C” and FG loops, respectively) are represented online in red, orange and purple, for the VH and in blue, green and bluegreen, for the VL (V-KAPPA (here) or V-LAMBDA). The CDR-IMGT lengths are [8.8.13] for VH and [6.3.9] for V-KAPPA (**B**) Trastuzumab CH1 (on the left) and C-KAPPA (on the right). Amino acids are shown in the one-letter abbreviation. Positions at which hydrophobic amino acids (hydropathy index with positive value: I, V, L, F, C, M, A) and tryptophan (W) are found in more than 50% of analysed sequences are shown online in blue. All proline (P) are shown online in yellow. Hatched circles correspond to missing positions according to the IMGT unique numbering for V domain [40,41] or C domain [42]. Arrows indicate the direction of the beta strands and their designations in 3D structures. Anchors are shown in squares. (With permission from M-P. Lefranc and G. Lefranc, LIGM, Founders and Authors of IMGT^®^, the international ImMunoGeneTics information system^®^, http://www.imgt.org).

**Figure 21:**
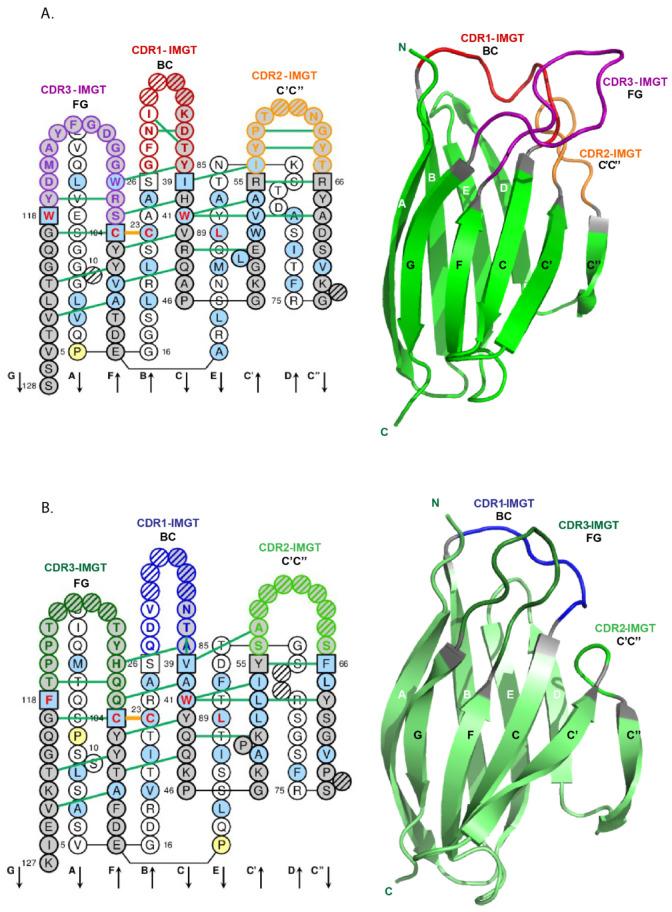
IMGT Collier de Perles of V-DOMAIN on two layers and ribbon representation [94]. (**A**) Trastuzumab VH. (**B**) Trastuzumab V-KAPPA. The IMGT Colliers de Perles on two layers (on the left) show, in the forefront, the GFCC′C′′ strands (forming the sheet located at the interface VH/VL) and, in the back, the ABED strands. The IMGT Colliers de Perles were generated using the IMGT/Collier-de-Perles tool [51] integrated in IMGT/3Dstructure-DB [57,58,59]. The CDR1-IMGT, CDR2-IMGT and CDR3-IMGT (corresponding to the BC, C’C” and FG loops, respectively) are represented in red, orange and purple, for the VH and in blue, green and bluegreen, for the VL, here V-KAPPA. The CDR-IMGT lengths are [8.8.13] for VH and [6.3.9] for V-KAPPA. Hatched circles correspond to missing positions according to the IMGT unique numbering for V domain [40,41]. Arrows indicate the direction of the beta strands and their designations in 3D structures. Anchors are shown in squares. Hydrogen bonds (green lines online were automatically added from the experimental structural data). The disulfide bridge (orange line between C23 and C104) was also automatically added from the experimental structural data. The ribbon representation of the 3D structures (on the right) was obtained using PyMOL (http://www.pymol.org). The identifiers of the chains to which the domains belong are 1n8z_B and 1n8z_A (IMGT^®^ http://www.imgt.org, IMGT/3Dstructure-DB). (With permission from M-P. Lefranc and G. Lefranc, LIGM, Founders and Authors of IMGT^®^, the international ImMunoGeneTics information system^®^, http://www.imgt.org).

The loop and strands are visualized in the IMGT Colliers de Perles [47,48,49,50,51,52] which can be displayed on 1 layer (closer to the amino acid sequence) (Figure 20) or on 2 layers (closer to the 3D structure) (Figure 21). The three loops, BC, C’C” and FG (or CDR1-IMGT, CDR2-IMGT and CDR3-IMGT for a V-DOMAIN) are delimited by the IMGT anchors. The six anchors belong to strands and comprise positions 26 and 39 (anchors of the BC loop or CDR1-IMGT), 55 and 66 (anchors of the C’-C’’ loop or CDR2-IMGT), 104 and 118 (anchors of the FG loop or CDR3-IMGT), and are shown in square in the IMGT Colliers de Perles. The CDR3-IMGT anchors are highly conserved, they are C104 (2nd-CYS, in F strand) and F118 or W118 (J-PHE or J-TRP in G strand). The JUNCTION of an IG or TR V-DOMAIN includes the anchors 104 and 118 and is therefore two amino acids longer than the corresponding CDR3-IMGT (positions 105-117).

#### 4.1.3. V-DOMAIN Strands and Loops (FR-IMGT and CDR-IMGT)

The V domain strands and loops and their delimitations and lengths, based on the IMGT unique numbering for V domain [39,40,41,44,45,46], are shown in Table 10. In the IG and TR V-DOMAIN, the three hypervariable loops BC, C’C” and FG involved in the ligand recognition (native antigen for IG and pMH for TR) are designated complementarity determining regions (CDR-IMGT), whereas the strands form the framework region (FR-IMGT), which includes FR1-IMGT, FR2-IMGT, FR3-IMGT and FR4-IMGT (Table 10). Correspondences between the IMGT unique numbering for V-DOMAIN [39,40,41] with other numberings, e.g., Kabat [118], or canonical structures [288,289,290], are available in the IMGT Scientific chart (IMGT^®^ http://www.imgt.org, IMGT Scientific chart > Numbering > Correspondence between V numberings).

For a V domain, the BC loop (or CDR1-IMGT in a V-DOMAIN) encompasses positions 27 to 38, the C’C’’ loop (or CDR2-IMGT in a V-DOMAIN) positions 56 to 65, and the FG loop (or CDR3-IMGT) positions 105 to 117. In a V-DOMAIN, the CDR3-IMGT encompasses the V-(D)-J junction that results from a V-J or V-D-J rearrangement [2,3] and is more variable in sequence and length than the CDR1-IMGT and CDR2-IMGT that are encoded by the V gene region only. For CDR3-IMGT of length >13 AA, additional IMGT positions are added at the top of the loop between 111 and 112 (Table 11) (IMGT^®^ http://www.imgt.org, IMGT Scientific chart > 2. Numbering > IMGT unique numbering for V-DOMAIN and V-LIKE-DOMAIN).

In biological data, the lengths of the loops and strands are given by the number of occupied positions (unoccupied positions or ‘IMGT gaps’ are represented with hatches in the IMGT Colliers de Perles (Figure 20A) or by dots in alignments). The CDR-IMGT lengths are given in number of amino acids (or codons), into brackets and separated by dots: for example VH [8.8.13] means that the BC, C’C” and FG loops (or CDR1-IMGT, CDR2-IMGT and CDR3-IMGT for a V-DOMAIN) have a length of 8, 8 and 13 amino acids (or codons), respectively. Similarly [25.17.38.11] means that the FR1-IMGT, FR2-IMGT, FR3-IMGT and FR4-IMGT have a length of 25, 17, 38 and 11 amino acids (or codons), respectively. Together, the four FR of a VH domain usually comprise 91 amino acids and the individual FR-IMGT lengths are [25.17.38.11], whereas the four FR of a VL domain usually comprise 89 amino acids and the individual FR-IMGT lengths are [26.17.36.10] (Figure 20A).

#### 4.1.4. V-DOMAIN Conserved Amino Acids

A V domain has five characteristic amino acids at given positions (positions with bold (online red) letters in the IMGT Colliers de Perles). Four of them are highly conserved and hydrophobic [81] and are common to the C domain: 23 (1st-CYS), 41 (CONSERVED-TRP), 89 (hydrophobic) and 104 (2nd-CYS). These amino acids contribute to the two major features shared by the V and C domain: the disulfide bridge (between the two cysteines 23 and 104) and the internal hydrophobic core of the domain (with the side chains of tryptophan W41 and amino acid 89). The fifth position, 118, is an anchor of the FG loop. It is occupied, in the V domains of IgSF other than IG or TR [19], by amino acids with diverse physicochemical properties [81]. In contrast, in IG and TR V-DOMAIN, the position 118 is occupied by remarkably conserved amino acids which consist in a phenylalanine or a tryptophan encoded by the J-REGION and therefore designated J-TRP or J-PHE 118. The bulky aromatic side chains of J-TRP and J-PHE are internally orientated and structurally contribute to the V-DOMAIN hydrophobic core [40,41].

#### 4.1.5. V-DOMAIN Delimitation 

A criterion used in the IMGT^®^ characterization of a V domain is its delimitation taking into account the exon delimitations, whenever appropriate. This IMGT^®^ genomic approach integrates the strands A and G which are usually absent of structural alignments [45]. The exon rule is not used for the delimitation of the 5′ end of the first N-terminal domain of proteins with a leader. In those cases, the 5′ end of the first N-terminal domain corresponds to the proteolytic site between the leader (L-REGION) and the coding region of the mature protein. This is the case of the V-DOMAIN of the IG and TR chains. The IG V-DOMAIN (VH, V-KAPPA, V-LAMBDA) is therefore delimited in 5′ by the proteolytic site which delimits the 5′ end of the V-REGION and in 3′ by the splicing site of the J-REGION of the rearranged V-D-J (for IGH) or V-J (for IGK or IGL) [45].

#### 4.1.6. Protein Displays for the V-REGION and J-REGION

**Figure 22:**
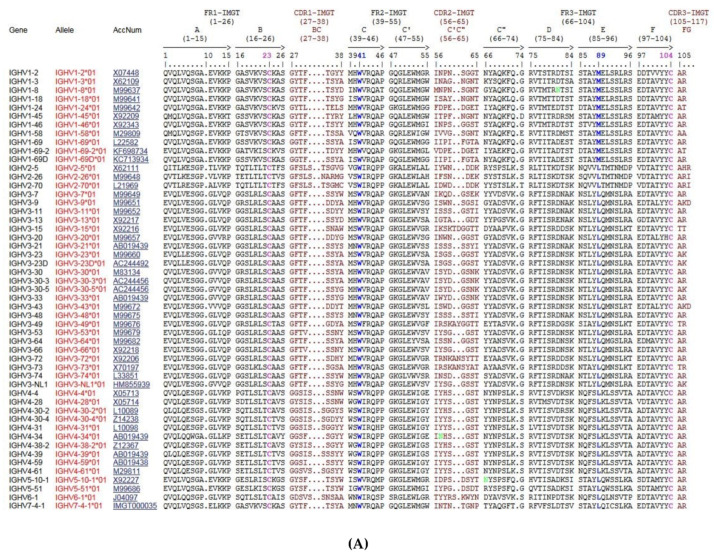

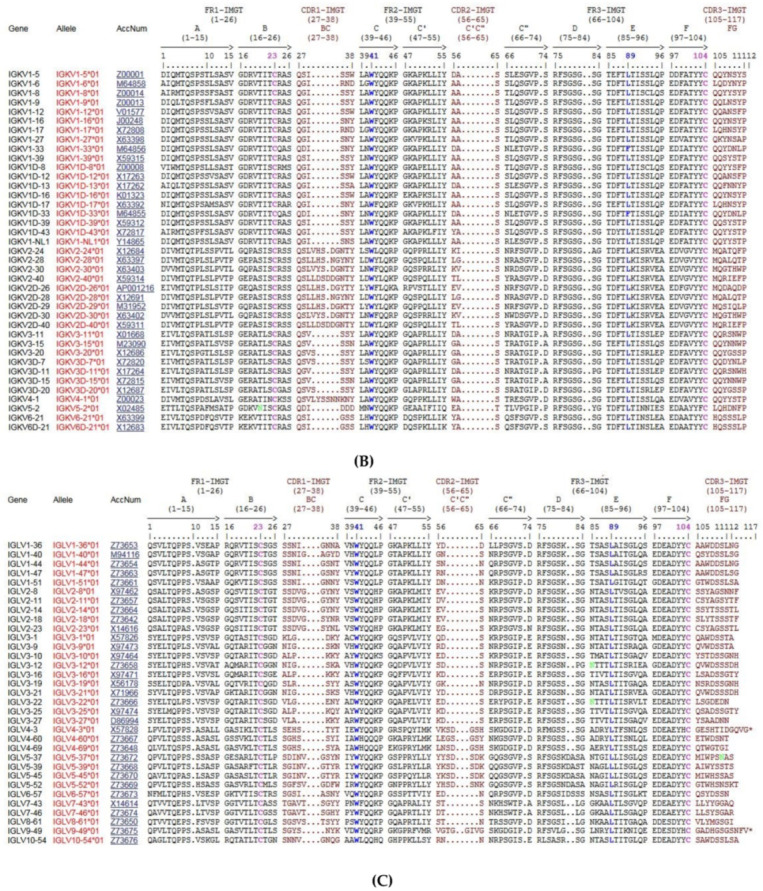
Protein displays of the *Homo sapiens* IGH, IGK and IGL V-REGION. Only the *01 allele of each functional or ORF V-REGION shown. (**A**). IGHV genes are listed per subgroup and, for each subgroup, increasing numbers corresponding generally to their position from 3′ to 5′ in the locus [2,237]. (**B**). IGKV genes are listed per subgroup and, for each subgroup, increasing numbers corresponding generally to their position from 3′ to 5′ in the locus, with the letter D for genes of the distal V-CLUSTER [2,237]. The IGKV2-29 gene is not represented the *01 allele being a pseudogene. For information, the IGKV2-29*02 F differs from IGKV2D-29*01 by P49 > S, N66 > S, S107 > G, Q109 > H [2,237]. (**C**). IGLV genes are listed per subgroup and, for each subgroup, increasing numbers corresponding generally to their position from 3′ to 5′ in the locus [2,237]. The header of the IMGT Protein display of V-REGION comprises 7 lines. Line 1: FR-IMGT and CDR-IMGT. Line 2: start and end positions of FR-IMGT and CDR-IMGT, e.g., (1-26). Line 3: name of strands (A, B…) and loops (AB, BC…). Line 4: start and end positions of strands, e.g., (1-15). Line 5: arrows (for the strands). Line 6: positions according to the IMGT unique numbering for V-DOMAIN and V-LIKE-DOMAIN [41]. Line 7: Pipes for the start and end positions of strands and loops and highlighted positions, dots for the other positions (dots indicate gaps according to the IMGT unique numbering [41]). The four conserved amino acids 23 (1st-CYS), 41 (CONSERVED-TRP), 89 (hydrophobic) and 104 (2nd-CYS) are highlighted (C23 and C104 in pink, W41 and hydrophobic 89 in blue). N (Asn, asparagine) of potential N-glycosylation sites (NXS/T, where X is different from P) (N-linked glycosylation) is shown in green. (With permission from M-P. Lefranc and G. Lefranc, LIGM, Founders and Authors of IMGT^®^, the international ImMunoGeneTics information system^®^, http://www.imgt.org).

The V-REGION of the IGHV, IGKV and IGLV genes in germline configuration are displayed in Protein displays (Figure 22) [2,237], with the FR1-IMGT, CDR1-IMGT, FR2-IMGT, CDR2-IMGT, FR3-IMGT and germline CDR3-IMGT. 

The J-REGION of the IGHJ, IGKJ and IGLJ genes in germline configuration are displayed in Protein displays, aligned on the conserved J-MOTIF ‘F/WGXG’ (where F is J-PHE, W is J-TRP, G, glycine, X, any amino acid, and G, glycine) [2,237] (IMGT^®^ http://www.imgt.org, IMGT Repertoire (IG and TR) > 2. Proteins and alleles > 1. Protein displays > V-REGION > IGHV, ibid. > IGKV, ibid. > IGLV; ibid. > J-REGION > IGHJ, ibid. IGKJ, ibid. IGLJ). The translation of the IGH D-REGION in the six frames are displayed in Alignments of alleles [2,223] (IMGT^®^ http://www.imgt.org, IMGT Repertoire (IG and TR) > Proteins and alleles > 2. Alignments of alleles > IGHD > Mammalia: human (*Homo sapiens*) > IGHD overview). 

### 4.2. IMGT Unique Numbering and IMGT Colliers de Perles for C Domain (NUMEROTATION)

#### 4.2.1. C Domain Definition and Main Characteristics

The C domain includes the C-DOMAIN of the IG and of the TR [2,3] and the C-LIKE-DOMAIN of the IgSF other than IG and TR. The C domain description of any receptor, any chain and any species is based on the IMGT unique numbering for C domain (C-DOMAIN and C-LIKE-DOMAIN) [42,44,45,46]. A C domain comprises about 90-100 amino acids and is made of seven antiparallel beta strands (A, B, C, D, E, F and G), linked by beta turns (AB, DE and EF), a transversal strand (CD) and two loops (BC and FG), and forming a sandwich of two sheets [ABED] [GFC] [42,44,45,46]. A C domain has a topology and a three-dimensional structure similar to that of a V domain but without the C’ and C’’ strands and the C’C’’ loop, which is replaced by a transversal CD strand [42]. 

#### 4.2.2. C Domain IMGT Colliers de Perles

**Figure 23:**
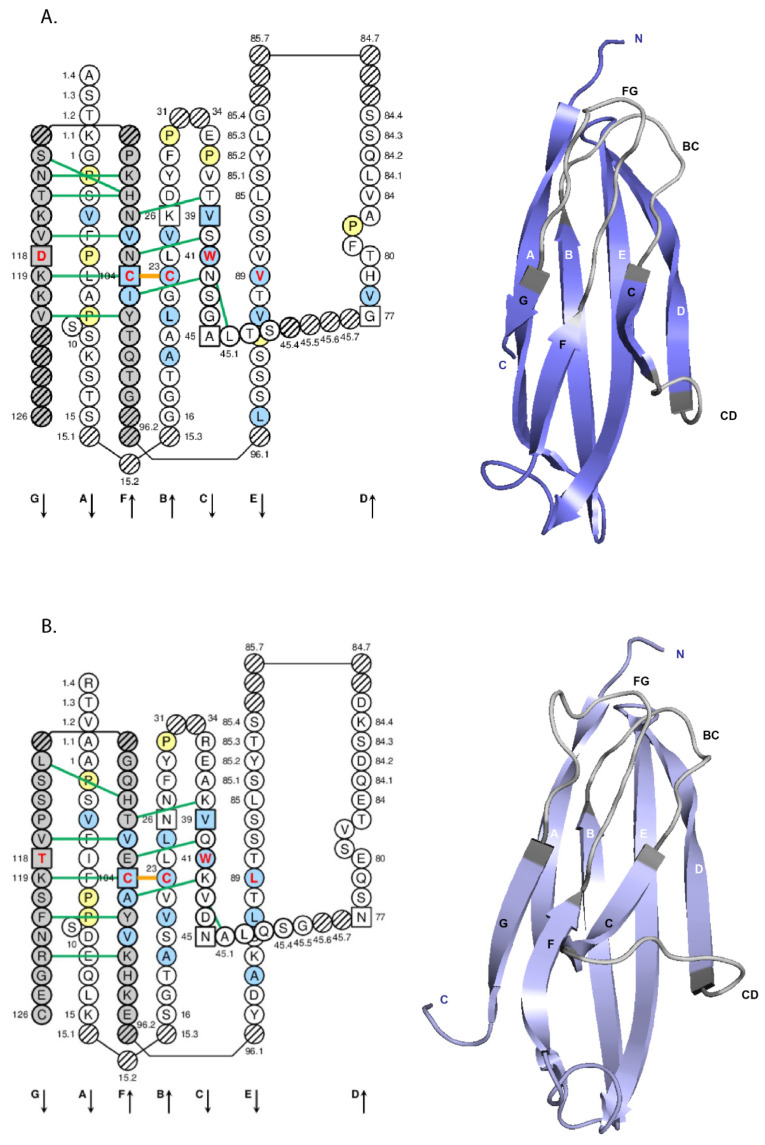
IMGT Collier de Perles of C-DOMAIN on two layers and ribbon representation [94]. (**A**) Trastuzumab CH1. (**B**) Trastuzumab C-KAPPA. The IMGT Colliers de Perles on two layers (on the left) show, in the forefront, the GFC strands and, in the back, the ABED strands (located at the interface CH1/C-KAPPA), linked by the CD transversal strand. The IMGT Colliers de Perles were generated by the IMGT/Collier-de-Perles tool [51] integrated in IMGT/3Dstructure-DB [57,58,59]. Hydrogen bonds (green lines online were automatically added from the experimental structural data). The disulfide bridge (orange line between C23 and C104) was also automatically added from the experimental structural data. Amino acids are shown in the one-letter abbreviation. Positions at which hydrophobic amino acids (hydropathy index with positive value: I, V, L, F, C, M, A) and tryptophan (W) are found in more than 50% of analysed sequences are shown online in blue. All proline (P) are shown online in yellow. Hatched circles correspond to missing positions according to the IMGT unique numbering for C domain [42]. Arrows indicate the direction of the beta strands and their designations in 3D structures. Anchors are shown in squares. The ribbon representation of the 3D structures (on the right) was obtained using PyMOL (http://www.pymol.org). The identifiers of the chains to which the domains belong are 1n8z_B and 1n8z_A (IMGT^®^ http://www.imgt.org, IMGT/3Dstructure-DB). (With permission from M-P. Lefranc and G. Lefranc, LIGM, Founders and Authors of IMGT^®^, the international ImMunoGeneTics information system^®^, http://www.imgt.org).

The lengths of the strands and loops are visualized in the IMGT Colliers de Perles [48,49,50,51,52], on one layer (Figure 20B) and two layers (Figure 23). There are six IMGT anchors in a C domain (four of them identical to those of a V domain): positions 26 and 39 (anchors of the BC loop), 45 and 77 (by extension, anchors of the CD strand as there is no C’-C’’ loop in a C domain [42], and 104 and 118 (anchors of the FG loop). 

#### 4.2.3. C Domain Strands and Loops

The C domain strands, turns and loops and their delimitations and lengths, based on the IMGT unique numbering for C domain [42,44,45,46], are shown in Table 12. Correspondences between the IMGT unique numbering with other numberings (Eu, Kabat [118]) are available in the IMGT Scientific chart (IMGT^®^ http://www.imgt.org, IMGT Scientific chart > Numbering > Correspondence between C numberings).

#### 4.2.4. C Domain Conserved Amino Acids

A C domain has five characteristic amino acids at given positions (positions with bold (online red) letters in the IMGT Colliers de Perles). Four of them are highly conserved and hydrophobic [81] and are common to the V domain: 23 (1st-CYS), 41 (CONSERVED-TRP), 89 (hydrophobic) and 104 (2nd-CYS). As mentioned above, these amino acids contribute to the two major features shared by the V and C domain: the disulfide bridge (between the two cysteines 23 and 104) and the internal hydrophobic core of the domain (with the side chains of tryptophan W41 and amino acid 89). The fifth position, 118, is diverse and is characterized as being a FG loop anchor.

#### 4.2.5. C Domain Genomic Delimitation 

In IMGT^®^, the C domains (C-DOMAIN and C-LIKE-DOMAIN) are delimited taking into account the exon delimitation, whenever appropriate. The exon/intron organization of the *Homo sapiens* IGHC genes shows that each CH C-domain corresponds to one exon [2] (IMGT^®^ http://www.imgt.org, IMGT Repertoire (IG and TR) > 1. Locus and genes > 5. Gene exon/intron organization > IGHC > Human). As for the V domain, this IMGT^®^ genomic approach integrates the strands A and G which are usually absent of structural alignments [45]. 

#### 4.2.6. C-REGION Protein Displays

**Figure 24:**
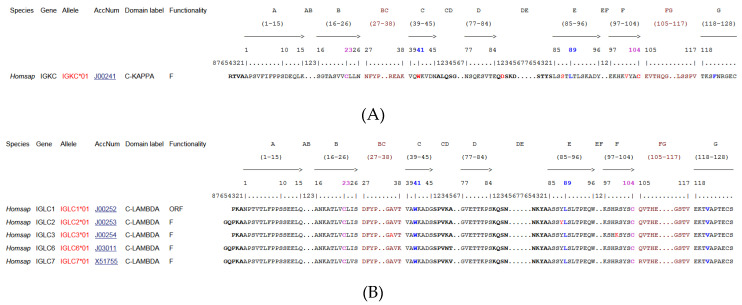

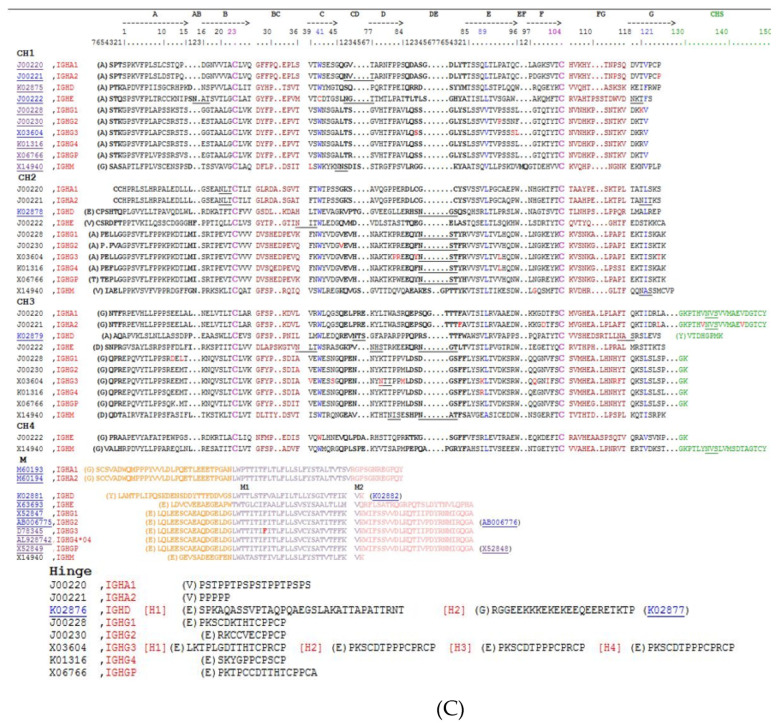
IMGT Protein displays of the *Homo sapiens* IGKC, IGLC and IGHC genes. (**A**). IGKC Protein display. Ala A45.1 and Val V101 characterize the Km3 allotype (see Section 3.3.4). (**B**). IGLC Protein display. Asn N1 and Thr T3 characterize the Mcg isotype marker, Gly G45 characterizes the Ke isotype marker, and Lys K100, the Oz isotype marker (see Section 3.4.4). (**C**). IGHC Protein display. The IGHC Protein display is shown per domain (CH1, CH2, CH3, and for IGHM and IGHE, CH4). The CO + TM + CY of the membrane IG (mIG) is encoded by two exons, excepted for IGHA1 and IGHA2 where it is encoded by a single exon. The CHS of the sIG is colored in green. The hinge region (presented here, at the bottom of the figure) is located between CH1 and CH2. It is encoded by one exon (in IGHG1, IGHG2, IGHG4, IGHGP genes), two exons (in IGHD gene), two to five exons (in IGHG3 gene, 4 for the most common haplotype, shown here). The hinge region of the IGHA1 and IGHA2 is fused in 5′ to the CH2 domain (encoded in a H-CH2 exon) [2]. The IGHGP, although an ORF, is shown for compleness. The C-KAPPA, C-LAMBDA and CH are numbered according to the IMGT unique numbering for C-DOMAIN and C-LIKE-DOMAIN [42]. The header of the IMGT Protein display of the C-DOMAIN obtained automatically (A and B) comprises 5 lines. Line 1: name of strands (A, B…), turns and loops (AB, BC…). Line 2: start and end positions of strands, e.g., (1-15). Line 3: arrows (for the strands). Line 4: positions according to the IMGT unique numberin [42]. Missing positions (32 and 33 in the BC loop and 46 to 76) in the C domain by comparison with the V domain are not shown. Line 5: Pipes for the start and end positions of strands and loops and highlighted positions, and dots for the other positions (dots indicate gaps according to the IMGT unique numbering [42]). The four conserved amino acids 23 (1st-CYS), 41 (CONSERVED-TRP), 89 (hydrophobic) and 104 (2nd-CYS) are highlighted (C23 and C104 in pink, W41 and hydrophobic 89 in blue). The IMGT Protein display of the *Homo sapiens* IGHC obtained manually (C) allows to display the CHS, the CO+TM+CY and the hinge. The N-linked glycosylation motifs (NXS/T, where X is different from P) are underlined. In the automatic version for the CH displayed online, the letter N (Asn, asparagine) of potential N-glycosylation sites are shown in green. (With permission from M-P. Lefranc and G. Lefranc, LIGM, Founders and Authors of IMGT^®^, the international ImMunoGeneTics information system^®^, http://www.imgt.org).

The Protein display of the C-REGION of the IGKC and IGLC corresponds to the C-KAPPA and C-LAMBDA domains (Figure 24A,B).The Protein display of the C-REGION of the IGHC genes (Figure 24C) is shown per CH domain and comprises, in addition to the 3 or 4 CH domains, the hinge region for the H-alpha, H-delta and H-gamma chains and, for the membrane IG (mIG), the region CO + TM + CY (encompassing the connecting region (CO), the transmembrane region (TM) and the cytoplasmic region (CY) (Table 1) with delimitation of the exons M or M1 and M2 [2]) and for the secreted IG (sIG), the CHS (expressed instead of CO + TM + CY).

## 5. IMGT^®^ Databases and Tools for IG Sequences and Structures

### 5.1. IMGT^®^, the International ImMunoGeneTics Information System^®^

**Figure 25:**
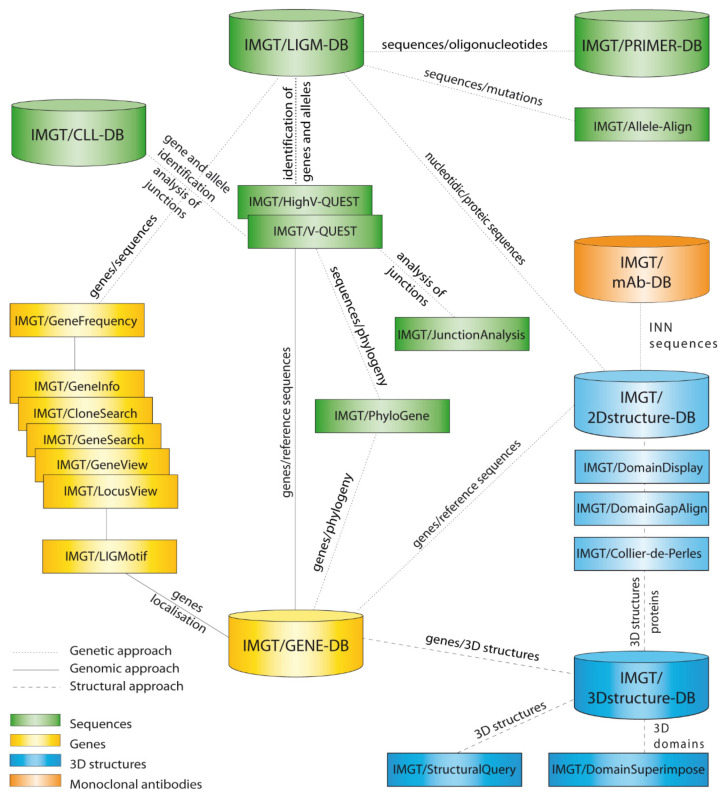
IMGT^®^, the international ImMunoGenetics information system^®^, http://www.imgt.org [1,94]. The IMGT Web resources (>25,000 pages, the IMGT Marie-Paule page) are not shown. IMGT/mAb-DB for therapeutic monoclonal antibodies and fusion proteins for immune applications (FPIA) has been online since December 4, 2009. IMGT/HighV-QUEST portal for next generation sequencing (NGS) high-throughput sequence analysis, created in October 2010, has been available on the web since November 22, 2010. (With permission from M-P. Lefranc and G. Lefranc, LIGM, Founders and Authors of IMGT^®^, the international ImMunoGeneTics information system^®^, http://www.imgt.org).

IMGT^®^, the international ImMunoGeneTics information system^®^ comprises seven databases [54,55,56,57,58,59,60] and 17 tools [61,62,63,64,65,66,67,68,69,70,71,72,73,74,75,76,77,78,79,80] and >25,000 pages of Web resources (Figure 25) [1,94].

### 5.2. IMGT^®^ Nucleotide Sequence and Repertoire Analysis

#### 5.2.1. IMGT/V-QUEST for Nucleotide Sequence Analysis

**Figure 26:**
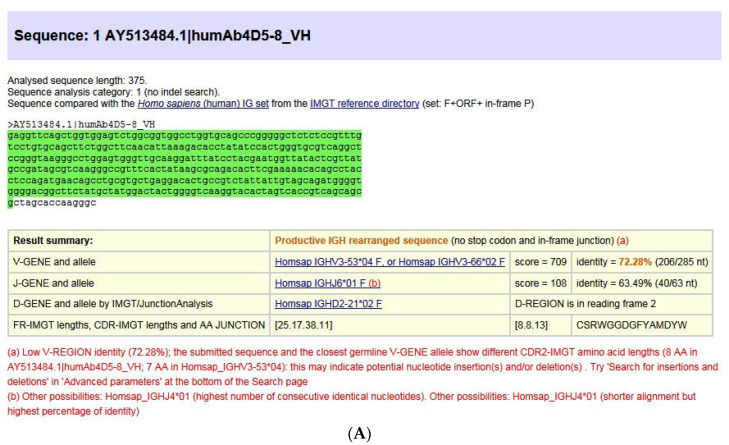

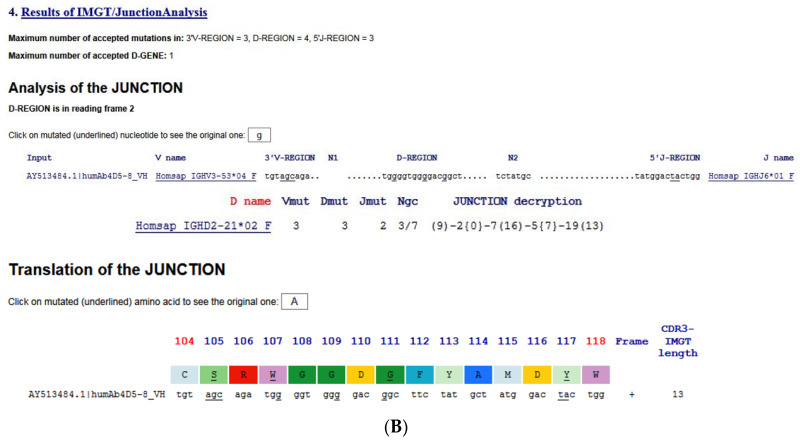
IMGT/V-QUEST. (**A**). Result summary. AY513484 is the nucleotide sequence of the VH domain of humAb4D5-8 (trastuzumab) (identified in IMGT/LIGM-DB [54,55] by the clone name). Comments in red are added automatically by the IMGT/V-QUEST tool. The sequence identified by IMGT/V-QUEST as containing a V domain in the user sequence, and therefore analysed by the tool, is displayed with a green background. (**B**). Results of IMGT/JunctionAnalysis. The JUNCTION decryption (3′V)3′{N1}5′(D)3′{N2}5′(5′J) (labels here for one D) provides region lengths (in nt) of (3′V), (D) and (5′J) (numbers between parentheses), of {N1} and {N2} (numbers between braces), and between these regions and at the 3′ or 5′ end of V, D or J, numbers indicating, either trimmed nt (negative (−) values) or palindromic P nucleotides (positive (+) values) (trimmed or P nt are mutually exclusive) (IMGT^®^ http://www.imgt.org, IMGT/JunctionAnalysis (doc) > IMGT/JunctionAnalysis Documentation). Color menu is according to the AA physicochemical properties [81]. (With permission from M-P. Lefranc and G. Lefranc, LIGM, Founders and Authors of IMGT^®^, the international ImMunoGeneTics information system^®^, http://www.imgt.org).

IMGT/V-QUEST [61,62,63,64,65,66] is the IMGT^®^ online tool for the analysis of IG and TR nucleotide sequence analysis [1]. The entry type corresponds to user nucleotide sequences of V domains (1-50 sequences per analysis), from rearranged gDNA or cDNA. IMGT/V-QUEST identifies the variable (V), diversity (D) and junction (J) genes in rearranged IG and TR sequences and, for the IG, the nucleotide (nt) mutations and amino acid (AA) changes resulting from somatic hypermutations by comparison with the IMGT/V-QUEST reference directories sets. (links available from the IMGT/V-QUEST Welcome page). The IMGT/V-QUEST reference directory sets include IMGT reference sequences (one per allele) from functional (F) genes and alleles, open reading frame (ORF) and pseudogenes (P) alleles with in-frame V-REGION. The tool integrates IMGT/JunctionAnalysis [67,68] for the detailed characterization of the V-D-J or V-J junctions, and IMGT/Automat [69,70] for a complete sequence annotation. The IMGT/V-QUEST tool functionalities include:

(1) Introduction of IMGT gaps, according to the IMGT unique numbering for V-DOMAIN (Section 4),
(2) Identification of the closest V, D and J genes and alleles, according to the IMGT gene and allele nomenclature (Section 3) (e.g., for *Homo sapiens* [2,4]) (Figure 26A),
(3) IMGT/JunctionAnalysis results [67,68] (Figure 26B),
(4) Description of mutations and amino acid changes [65].

The amino acid (AA) changes are described for the hydropathy (three classes), volume (five classes) and physicochemical properties (11 classes) [81] (IMGT^®^ http://www.imgt.org, IMGT Education > IMGT Aide-mémoire > Amino acids > IMGT classes of the 20 common amino acids). S40 > G (++−) means that the two AA involved in the change (S > G) at codon 40 belong to the same hydropathy (+) and volume (+) classes but to different physicochemical properties (−) classes [81]. This qualification of AA replacement has led to the identification of four types of AA changes: very similar (+++), similar (++−, +−+), dissimilar (−−+, −+−, +−−), and very dissimilar (−−−).

(5) Identification of indels and their correction [65] (option),
(6) IMGT/Automat annotation [69,70] (annotations are those found in IMGT/LIGM-DB flatfiles),
(7) IMGT Colliers de Perles [51].

Users can choose the option ‘Search for insertions and deletions in V-REGION’ [65] (1-50 sequences per run) in Advanced parameters. The option ‘Analysis of single chain Fragment variable (scFv) [75], available in Advanced functionalities, allows the analysis of long read scFv sequences from combinatorial libraries containing two V-DOMAIN. Customized parameters and results provided by IMGT/V-QUEST and IMGT/JunctionAnalysis have been described elsewhere [61,62,63,64,65,66].

IMGT/V-QUEST is frequently used by clinicians for the analysis of the somatic hypermutations in leukemia, lymphoma and myeloma, and more particularly in chronic lymphocytic leukemia (CLL) [291,292,293] in which the percentage of mutations in the patient VH has a prognostic value. The sequences of the V-(D)-J junctions determined by IMGT/JunctionAnalysis are also used in the characterization of stereotypic patterns in CLL and B cell lymphoproliferations [291,292,293,294] and for the junction synthesis of specific probes for the follow-up of residual diseases in leukemias and lymphomas.

#### 5.2.2. IMGT/HighV-QUEST

IMGT/HighV-QUEST basic functionalities for NGS repertoire analysis: IMGT/HighV-QUEST [66,71,72,73,74] is the high-throughput version of IMGT/V-QUEST. It is so far the only online portal available for the direct analysis of long IG and TR sequences from next generation sequencing (NGS) [1]. The submitted entries are user long read nucleotide sequences of V domains (1,000,000 sequences per run). IMGT/HighV-QUEST uses the same algorithms and IMGT reference directories (and therefore provides the same degree of resolution and high quality results) as IMGT/V-QUEST. The tool works for the IG of any species for which an IMGT reference directory is available [1]. The option ‘Analysis of single chain Fragment variable (scFv)’ allows the analysis of scFv long read sequences which contain two V domains [75], and the repertoire analysis of phage display scFv combinatorial libraries [295,296]. The IMGT/HighV-QUEST basic functionalities include:

(1). Introduction of IMGT gaps, according to the IMGT unique numbering for V-DOMAIN [41] (Section 4),
(2). Identification of indels and their correction [65] (by default),
(3). Identification of the closest V, D and J genes and alleles, according to the IMGT gene and allele nomenclature (Section 3) (e.g., for *Homo sapiens* [2,4]),
(4). IMGT/JunctionAnalysis results [67,68],
(5). Description of mutations and amino acid changes [65],
(6). IMGT/Automat annotation [69,70].

The IMGT/HighV-QUEST output is provided in eleven results files in CVS format (results equivalent to those of the Excel file from IMGT/V-QUEST online) (Table 13). A twelfth file ‘scFv’ is only present if ‘Analysis of single chain Fragment variable (scFv)’ was selected in Advanced functionalities).

**Figure 27:**
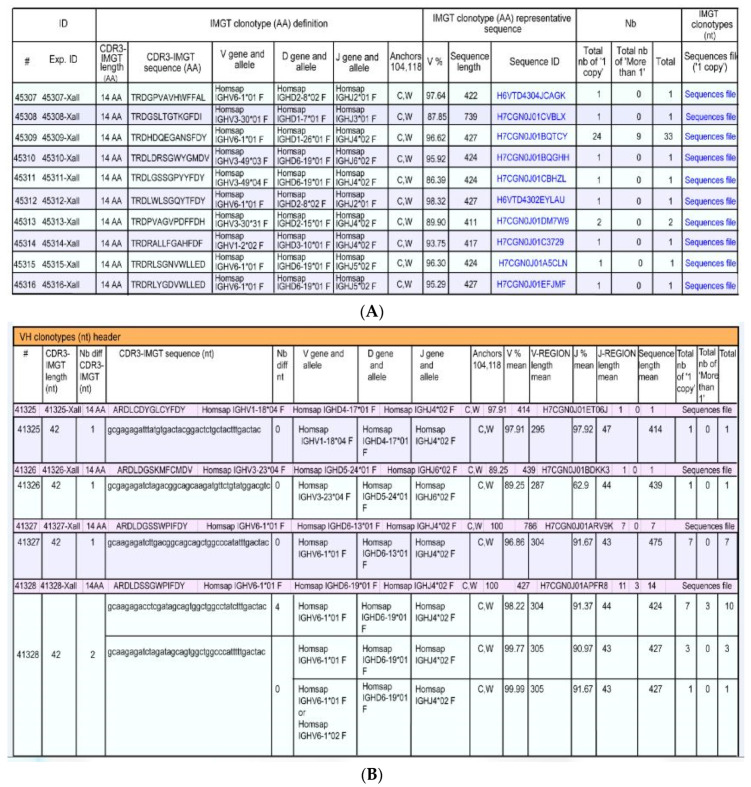
Displays of (**A**) IMGT clonotypes (AA) and (**B**) IMGT clonotypes (nt). IMGT clonotypes (AA) and (nt) are characterized by the IMGT/HighV-QUEST statistical module [66,73]. Displays are based on CDR3-IMGT lengths. In B, the pink lines correspond to IMGT clonotypes (AA) with, below each one of them, the corresponding IMGT clonotypes (nt). (With permission from M-P. Lefranc and G. Lefranc, LIGM, Founders and Authors of IMGT^®^, the international ImMunoGeneTics information system^®^, http://www.imgt.org).

The outputs of the IMGT/HighV-QUEST can be statistically analysed (two batches up to 1 million sequences, comprising outputs of different sets) for the identification and characterization of the IMGT clonotypes (AA) online [73] (Figure 27) and their comparison between sets.

An IMGT clonotype (AA) is defined by:

- a unique V-(D)-J-rearrangement (V and J genes and alleles) (nt)
- a unique CDR3-IMGT (AA)
- conserved anchors 104, 118 (C104, W118 or F118)

The IMGT clonotype (AA) definition includes the IMGT names of the V, D, J genes and alleles, the CDR3-IMGT length (AA), the CDR3-IMGT sequence (AA), and the anchors 104 and 118 of the junction (i.e., for VH, ‘C, W’ for 2nd-CYS 104 and J-TRP 118, respectively) [73].

IMGT clonotype (AA and nt) results per locus are provided in 10 sections (HTML pages) (IMGT^®^ http://www.imgt.org, IMGT/HighV-QUEST > Documentation):

(1). IMGT clonotypes (AA) per Nb
(2). IMGT clonotypes (AA) per Nb with detailed clonotypes (nt)
(3). IMGT clonotypes (AA) per V gene
(4). IMGT clonotypes (AA) per V gene with detailed clonotypes (nt)
(5). IMGT clonotypes (AA) per CDR3-IMGT length (AA)
(6). IMGT clonotypes (AA) per CDR3-IMGT length (AA) with detailed clonotypes (nt)
(7). IMGT clonotypes (AA) by CRD3-IMGT sequence (AA) alphabetical order with detailed clonotypes (nt)
(8). IMGT clonotype (AA) diversity and expression histograms: per V, (D), J-GENE and per CDR3-IMGT length
(9). IMGT clonotype (AA) diversity and expression tables: per V, (D), J-GENE and per CDR3-IMGT length
(10). V gene and allele table: Rearrangements, Nb of sequences and Nb IMGT clonotypes (AA) per V-GENE and allele

Figure 27 shows a very small part of a list of 64,093 unique IMGT clonotypes (AA) identified in a case study. The display is based on the CDR3-IMGT length and corresponds to 5. and 6. in the list above. Each IMGT clonotype (AA) of a dataset, identified and defined by IMGT/HighV-QUEST, has an ‘Exp. ID’ identifier. For example, #45307, with an Exp. ID ‘45307-Xall’, is defined by a CDR3-IMGT length (AA) of ‘14 AA’, a CDR3-IMGT sequence (AA) ‘TRDGPVAVHWFFAL’, the ‘IGHV6-1*01 F – IGHD2-8*02 F – IGHJ2*01 F’ rearrangement, and conserved anchors 104 and 118 ‘C, W’. The IMGT clonotype (AA) representative sequence [73] has a V-REGION which is 97.64% identical to that of the germline IGHV6-1*01 and a length of 422 nt. The link to a ‘Sequence ID’ gives access to the representative sequence in FASTA format; the three following numbers indicates the number of sequences assigned to an IMGT clonotype (AA) (‘1 copy’, ‘More than 1’ and Total) [73]. ‘Sequences file’ gives access to a file containing the ‘1 copy’ sequences assigned to a given IMGT clonotype (AA), in FASTA format. In Figure 27B, IMGT clonotypes (AA) (in the pink horizontal lines) are displayed with the corresponding IMGT clonotypes (nt). For example, the ‘IMGT clonotype (AA)’ #45325, with an Exp. ID ‘45325_Xall’, is defined as above (CDR3-IMGT length (AA) ‘14 AA’, CDR3-IMGT sequence (AA) ‘ARDLCDYGLCYFDY’, ‘IGHV1-18*04 F-IGHD4-17*01 F-IGHJ4*02 F’ rearrangement, conserved anchors 104 and 118 ‘C, W’, V-REGION representative sequence 97.91% identical to that of the germline IGHV1-18*04, and length of 414 nt. ‘Sequence ID’, the three following numbers, ‘Sequences file’ are as above. The header for the description of the VH clonotypes (nt) is given at the top. The nb of different CDR3-IMGT (nt) indicates the nb of IMGT clonotypes (nt) for a given IMGT clonotype (AA) (e.g., 2 for #41328). The CDR3-IMGT sequence (nt) is shown with the number of different nucleotides (‘Nb diff nt’) by comparison with that of the representative sequence, ‘0′ indicates that the CDR3-IMGT sequence (nt) is identical to that of the IMGT clonotype (AA) representative sequence. For #41328, there is an IMGT clonotype (nt) with 4 nt differences (‘c’ instead of ‘t’ at position 9, ‘c’ instead of ‘a’ at position 12, ‘t’ instead of ‘c’ at position 30 and ‘c’ instead of ‘t’ at position 33) compared to the CDR3-IMGT of the representative sequence. #41328 also shows an example of ‘several alleles’ (for V and J) (2 alleles for IGHV) assigned to an IMGT clonotype (AA).

To date (July 10, 2020), more than 21 billion of sequences were analysed by IMGT/HighV-QUEST, by 3177 users from 46 countries (43% users from USA, 34% from EU, 22% from other parts of the world).

**Figure 28:**
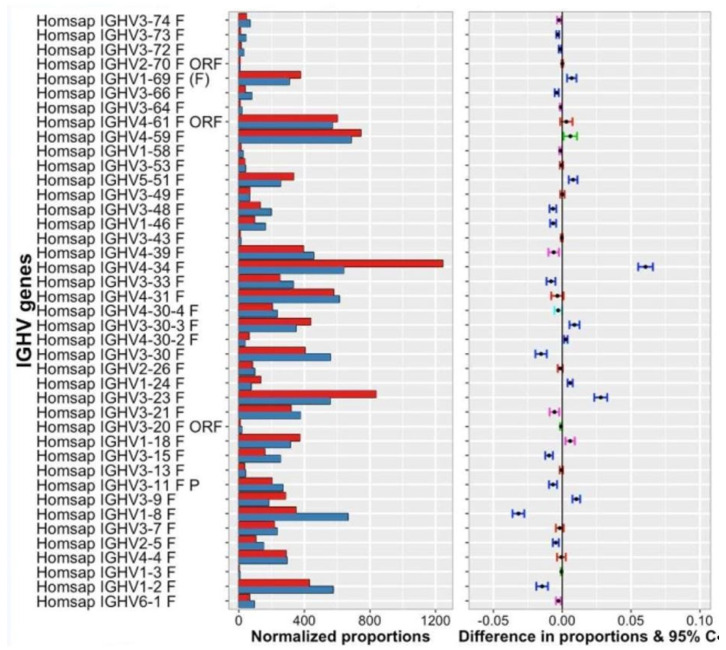
Results of IMGT/StatClonotype [76,77]. Differences in proportions graph. Genes are ordered by their position in the locus with the IMGT functionality [2]. The differences in proportions graph with significance and confidence interval (CI) are shown for *Homo sapiens* IGH IMGT clonotypes (AA) with a given gene, between two sets of B cell populations. Negative differences in proportions are shown on the left of the vertical line (abscissa equal to 0) and positive differences are shown on the right of the vertical line. CI bars colors correspond to the test interpretation before adjustment of *p* - values, red (non-significant) and cyan (significant) (rawp), significant differences validated by the IMGT/StatClonotypes seven procedures (All_p): dark blue, by two or more multiple testing procedures (Min_2p): pink, and only by Benjamini & Hochberg (BH) (Only_BH): green). (With permission from M-P. Lefranc and G. Lefranc, LIGM, Founders and Authors of IMGT^®^, the international ImMunoGeneTics information system^®^, http://www.imgt.org).

Results from the IMGT/HighV-QUEST statistical analysis and IMGT clonotypes (AA) characterization can be analysed by the downloadable package IMGT/StatClonotype [76,77] which provides pairwise evaluation and visualisation comparison of NGS IG and TR IMGT clonotype (AA) diversity or expression (Figure 28).

### 5.3. IMGT^®^ Amino Acid Sequence Analysis and Representation

#### 5.3.1. IMGT/DomainGapAlign

IMGT/DomainGapAlign [58,79,80] is the IMGT^®^ online tool for the analysis of amino acid sequences and 2D structures of domains (e.g., V and C for IG) [1]. It is very popular in antibody humanization as it allows the comparison of the user V domain against reference sequences of V-REGION and J-REGION of genes and alleles of *Homo sapiens* and other vertebrate species (e.g., mouse, rat) and the delimitation and characterization of the FR-IMGT and CDR-IMGT.

IMGT/DomainGapAlign analyses amino acid domain sequences by comparison with the IMGT reference directory sets (translation of the germline V and J genes and of the C gene domains]. These reference amino acid sequences can be displayed by querying IMGT/DomainDisplay online. Several amino acid sequences can be analysed simultaneously in IMGT/DomainGapAlign, provided that they belong to the same domain type.

IMGT/DomainGapAlign displays the user V domain sequences aligned with the closest V and J regions, with IMGT gaps and delimitations of the strands and loops and the FR-IMGT and CDR-IMGT, according to the IMGT unique numbering [41]. If several closest genes and/or alleles are identified, the user can select the display of each corresponding alignment. The user amino acid sequence is displayed, according to the IMGT color menu, with the delimitations of the V-REGION, J-REGION, and for VH domains, (N-D)-REGION (identified by the tool by comparison with the delimitations of the closest V and J gene and allele). The characteristics of the AA changes [81] are shown in strands and loops and in FR-IMGT and CDR-IMGT. Clicking on the user sequence name in the alignment gives access to the IMGT/Collier-de-Perles tool which automatically provides the IMGT Collier de Perles of the analysed VH or VL domain (V-D-J region or V-J region, respectively) with highlighted amino acid differences (in pink circles online) with the closest germline sequence.

IMGT/DomainGapAlign analyses the user C domain sequences with similar functionalities: alignments and identification of the genes and alleles with the closest C domain, delimitation of the C-DOMAIN in the user sequence, characteristics of the AA changes in strands, turns and loops, IMGT Collier de Perles of the C-DOMAIN with highlighted amino acid differences (in pink circles online) with the closest reference sequence.

**Figure 29:**
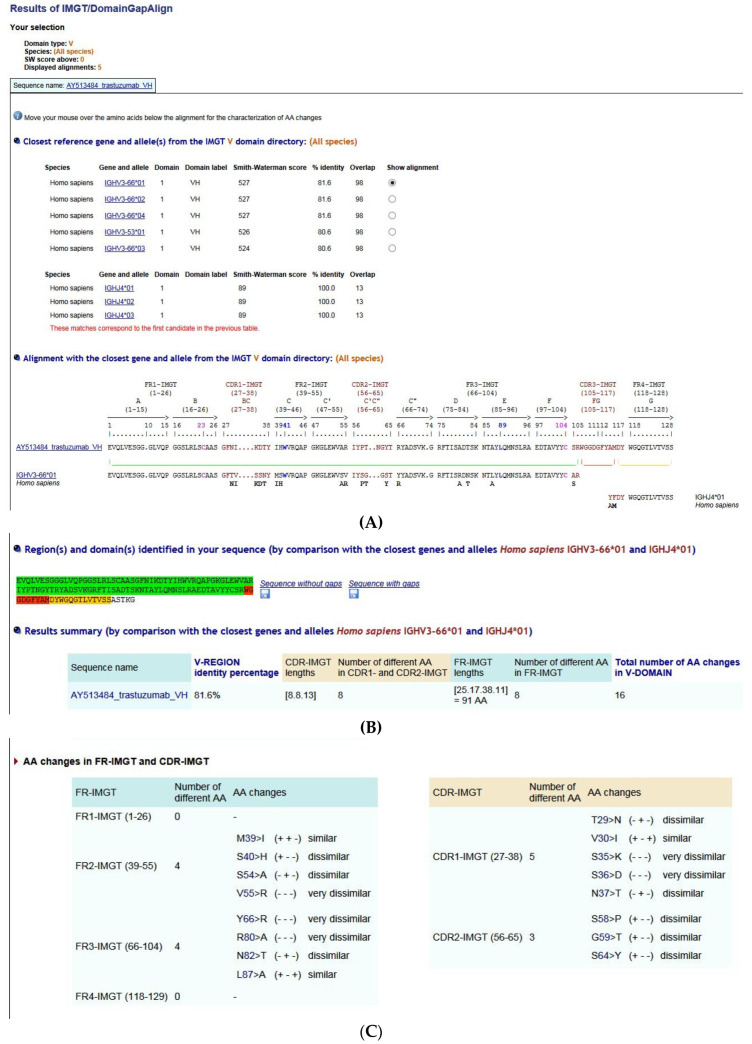
IMGT/DomainGapAlign [58,79,80]. (**A**). Analysis of the translated AY513484 sequence (trastuzumab VH). The analysis at the amino acid level gives the same percent of identity and same score for three alleles of IGHV3-66*02 (the one identified by nucleotide analysis, as well as *01 and *04. Analysis at the amino acid level gives IGHJ4*01 as the closest IGHJ gene and allele, mentioned in the IMGT/V-QUEST comments (in red in Figure 26A). The results are presented according to the IMGT Protein display header for V-DOMAIN [41]. (**B**). Regions and domains identified by IMGT/DomainGapAlign in the trastuzumab VH (V-REGION in green, N-AND-D-REGION in red and J-REGION in orange) and Results summary (by comparison with the closest genes and alleles IGHV3-66*01 and IGHJ4*01). Results summary comprises the V-REGION identity percentage (81.6%), CDR-IMGT lengths [8.8.13], Number of different AA changes in CDR1-IMGT and CDR2-IMGT, FR-IMGT lengths, Number of different AA in FR-IMGT and Total number of AA change in V-DOMAIN. (**C**). AA changes in FR-IMGT and CDR-IMGT with similarity characteristics. (With permission from M-P. Lefranc and G. Lefranc, LIGM, Founders and Authors of IMGT^®^, the international ImMunoGeneTics information system^®^, http://www.imgt.org).

The IMGT/DomainGapAlign tool functionalities include:

(1). Introduction of IMGT gaps, according to the IMGT unique numbering for V-DOMAIN [41] or for C-DOMAIN [42] (Section 4),
(2). Identification of the closest V, D and J genes and alleles, according to the IMGT gene and allele nomenclature (Section 3) (e.g., for *Homo sapiens* [2,4]) (Figure 29A),
(3). Delimitations of the domains, according to the IMGT unique numbering for V-DOMAIN [41] or for C-DOMAIN [42] (Section 4) (Figure 29B),
(4). Description of AA changes (Figure 29C),
(5). IMGT Collier de Perles [47,48,49,50] with highlighted AA changes (pink circles online) created by the IMGT/Collier-de-Perles tool [51].

IMGT/DomainGapAlign is widely used for the amino acid sequence analysis and standardized comparison for antibody humanisation and engineering using IMGT gene and allele nomenclature and IMGT unique numbering [88,89,90,91,92,93,94,95,96,97,98,99,100]. in the annotation of the IMGT/2Dstructure-DB and IMGT/3Dstructure-DB [57,58,59] entries with reciprocal links to IMGT/mAb-DB [60], and for the WHO INN description of therapeutic monoclonal antibodies [86,87].

#### 5.3.2. IMGT/Collier-de-Perles Tool

The IMGT/Collier-de-Perles tool [51], on the IMGT^®^ Web site at http://www.imgt.org (IMGT tools), allows the user to draw IMGT Colliers de Perles [47,48,49,50], on one or two layers, starting from their own domain amino acid sequences. Sequences have to be gapped according to the IMGT unique numbering (using for example IMGT/DomainGapAlign [58,79,80]). IMGT/Collier-de-Perles tool can be customized to display the CDR-IMGT according to the IMGT color menu and the amino acids according to their hydropathy or volume, or to the eleven IMGT physicochemical classes [81]. (IMGT^®^ http://www.imgt.org, IMGT Education > IMGT Aide-Mémoire > Amino acids > IMGT classes of the 20 common amino acids). IMGT color menu for the CDR-IMGT of a V-DOMAIN indicates the type of rearrangement, V-J or V-D-J [2,3]. Thus, the IMGT color menu for CDR1-IMGT, CDR2-IMGT and CDR3-IMGT, is red, orange and purple for VH (encoded by a V-D-J-REGION resulting from a V-D-J rearrangement), and blue, green and greenblue for V-KAPPA or V-LAMBDA (encoded by a V-J-REGION resulting from a V-J rearrangement). The IMGT/Collier-de-Perles tool is incorporated in IMGT/V-QUEST [61,62,63,64,65,66] (users start from IG and TR V domain nucleotide sequences), IMGT/DomainGapAlign [58,79,80] (users start from V, C and G amino acid sequences).

IMGT Colliers de Perles for V, C and G domains are provided in IMGT/2Dstructure-DB (for amino acid sequences in the database) and in IMGT/3Dstructure-DB (on two layers with hydrogen bonds for the V or C domains or with the pMH contact sites for the G domains, for 3D structures) [57,58,59].

### 5.4. IMGT^®^ Structure and Amino Acid Databases

#### 5.4.1. IMGT/3Dstructure-DB

IMGT/3Dstructure-DB [57,58,59], on the IMGT^®^ Web site at http://www.imgt.org, provides IMGT annotations and contact analysis of three-dimensional structure data of IG, TR, MH, and RPI and their complexes. The ‘PDB code’ (four letters and/or numbers, e.g., 1n8z) is used as ‘IMGT entry ID’ for the 3D structures obtained from the Research Collaboratory for Structural Bioinformatics (RCSB) Protein Data Bank (PDB) [297]. The IMGT/3Dstructure-DB card (per entry) provides eight search/display options: ‘Chain details’, ‘Contact analysis’, ‘Paratope and epitope’ [298,299], ‘3D visualization Jmol or QuickPDB’, ‘Renumbered IMGT files’, ‘IMGT numbering comparison’, ‘References and links’, ‘Printable card’.

**Figure 30:**
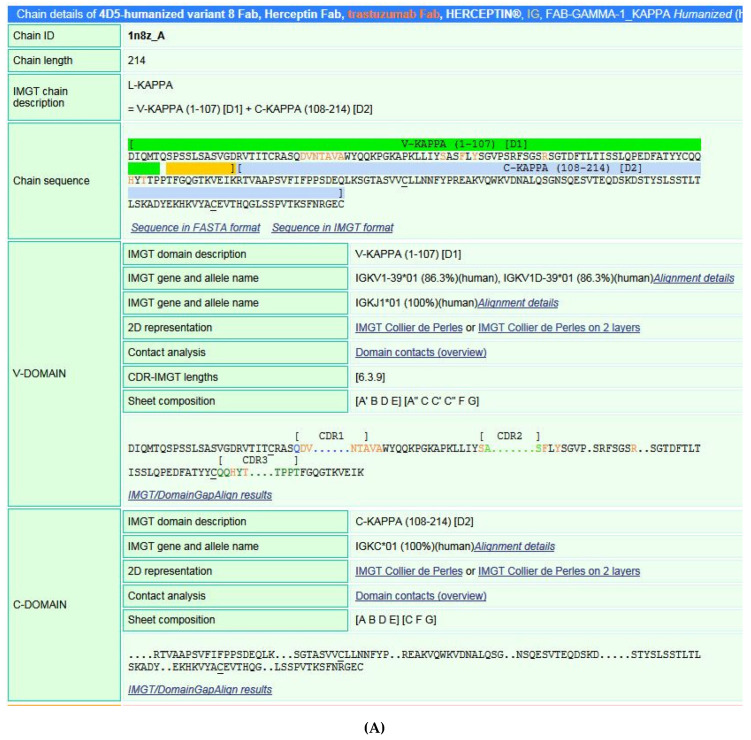

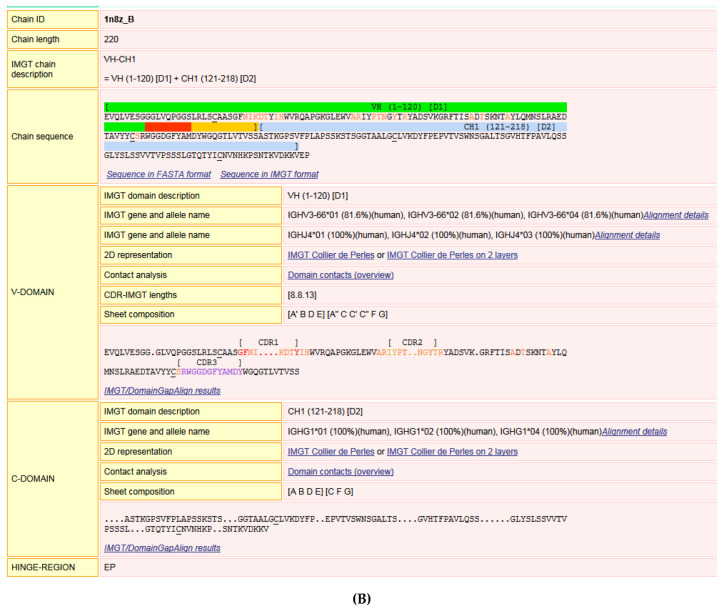
Chain details of the trastuzumab Fab ‘1n8z’ in IMGT/3Dstructure-DB [57,58,59]. (**A**). L-KAPPA chain (1b8z_A). The chain sequence is identified as containing a V-KAPPA (V-REGION in green and J-REGION in orange) and a C-KAPPA (blue). (**B**). VH-CH1 chain (1n8z_B). The chain sequence is identified as containing a VH (V-REGION in green, N-AND-D-REGION in red and J-REGION in orange) and a CH1 (in blue). (With permission from M-P. Lefranc and G. Lefranc, LIGM, Founders and Authors of IMGT^®^, the international ImMunoGeneTics information system^®^, http://www.imgt.org).

‘Chain details’ provides detailed IMGT annotation which includes the IMGT gene and allele identification (CLASSIFICATION), region and domain delimitations (DESCRIPTION) and amino acid (AA) positions according to the IMGT unique numbering. The closest IMGT genes and alleles expressed in the AA sequences of the 3D structures are identified by aligning the AA sequences of the 3D structures with the IMGT domain reference directory. The ‘1n8z’ comprises the trastruzumab Fab chains (L-KAPPA ‘1n8z_A’ and the VH-CH1 ‘1n8z_B’ (Figure 30). The Fab is in complex with the *Homo sapiens* ERRB2 (erb-b2 receptor tyrosine kinase 2, HER2, NEU, CD340) (Ligand ‘1n8z_C’) [300].

**Figure 31:**
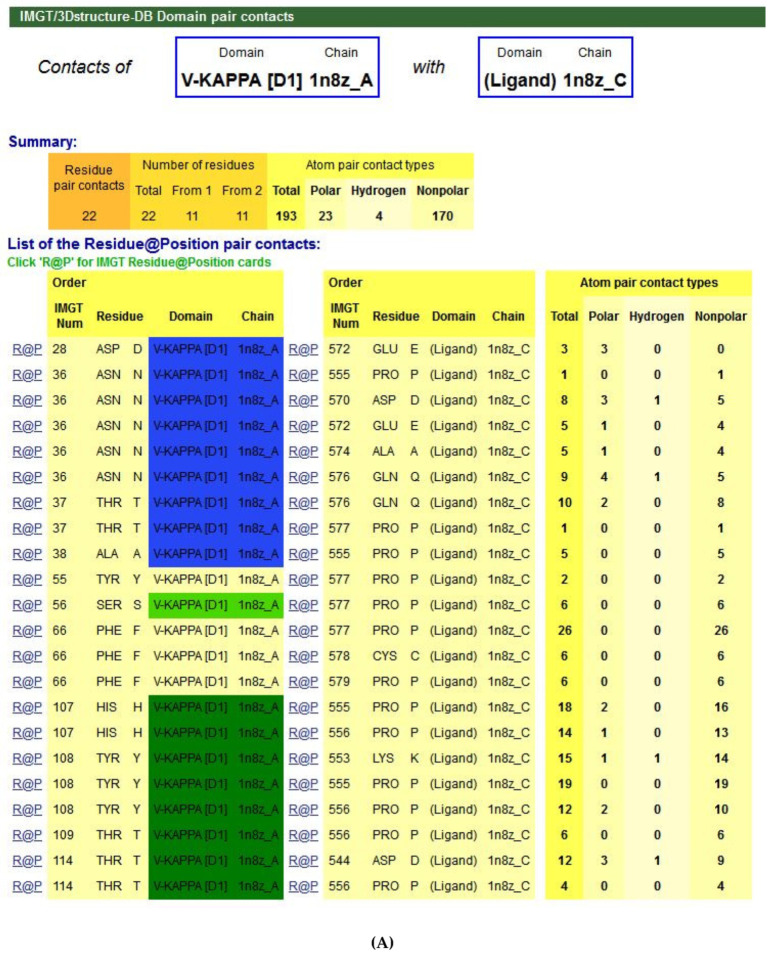

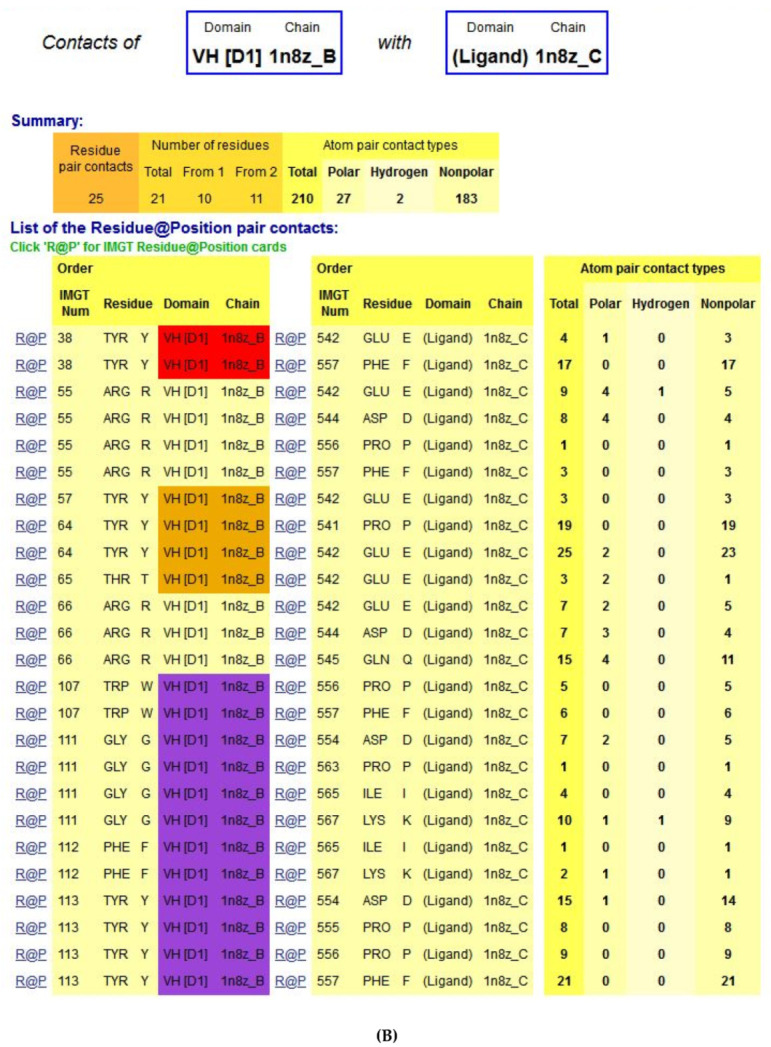
IMGT/3Dstructure-DB Domain pair contacs [57,58,59]. (**A**). Domain pair contacts between trastuzumab Fab V-KAPPA (1n8z_A) and ERBB2 ((Ligand) 1n8z_C). (**B**). Domain pair contacts between trastuzumab Fab VH (1n8z_B) and ERBB2 ((Ligand) 1n8z_C). Positions in the CDR1-IMGT, CDR2-IMGT and CDR3-IMGT are represented in blue, green and bluegreen, for the V-KAPPA and in red, orange and purple, for the VH. Positions in the FR-IMGT are in yellow. 55 and 66 are anchors of the CDR2-IMGT. Clicking on ‘R@P’ gives access to the IMGT Residue@Position card. (With permission from M-P. Lefranc and G. Lefranc, LIGM, Founders and Authors of IMGT^®^, the international ImMunoGeneTics information system^®^, http://www.imgt.org).

‘Contact analysis’ includes the ‘Domain pair contacts’ and the contacts between amino acids at given positions or ‘IMGT Residue@Position’. Domain pair contacts between the trastuzumab Fab V-KAPPA [D1] 1n8z_A and ERBB2 (‘(Ligand)’ 1n8z_C) and between the trastuzumab Fab VH [D1] 1n8z_B and ERBB2 (‘(Ligand)’ 1n8z_C) are shown in Figure 31. An ‘IMGT Residue@Position’ is defined by the IMGT position numbering in a domain (or if not characterized, in the chain), the AA name (3-letter and between parentheses 1-letter abbreviation), the IMGT domain description and the IMGT chain ID, e.g., ’57 – TYR (Y) – VH – 1n8z_B’. Its characteristics are reported in an IMGT Residue@Position card (or ‘R@P’) which includes (i) general information (PDB file numbering, IMGT file numbering, residue full name and formula), (ii) structural information ‘IMGT LocalStructure@Position’ (secondary structure, Phi and Psi angles (in degrees) and accessible surface area (ASA) (in square angstrom)) and (iii) detailed contact analysis. Contact analysis of IG/antigen complexes, is provided with detailed and standardized description of paratope/epitope in crystal structures. 

‘Renumbered IMGT flat file’ allows to view (or download) an IMGT coordinate file renumbered according to the IMGT unique numbering and to which has been added the IMGT specific information. This IMGT information (identical to that provided in ‘Chain details’) is in the ‘REMARK 410’ lines (blue online) added in the IMGT coordinate files. Tools associated to IMGT/3Dstructure-DB include IMGT/StructuralQuery and IMGT/DomainSuperimpose, available online.

**Figure 32:**
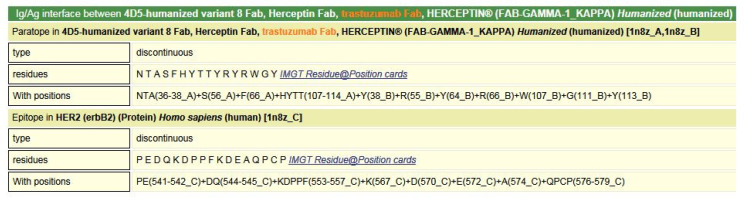
IMGT/3Dstructure-DB paratope/epitope of trastuzumab/ERBB2 in 1n8z [57,58,59]. The IMGT paratope, or antigen-binding site, include the part of the VH and VL domains that recognizes (binds to) the antigen (Ag) (epitope or antigenic determinant) [298,299]. In addition to the contacts between amino acids of the CDR1-IMGT, CDR2-IMGT and CDR3-IMGT of the V-KAPPA and VH and the antigen ERBB2 (Figure 31), framework positions which are detected as having relevant contacts with the antigen are included in the paratope. This is the case, here, of the anchor positions of the CDR2-IMGT in V-KAPPA F(66_A) (Figure 31A) and in VH R(55_B) and R(66_B) (Figure 31B). These posiions are classically taken into account in V domain humanization by grafting. (With permission from M-P. Lefranc and G. Lefranc, LIGM, Founders and Authors of IMGT^®^, the international ImMunoGeneTics information system^®^, http://www.imgt.org).

In summary, the IMGT/3Dstructure-DB provides:

(1) Identification of the closest genes and alleles, according to the IMGT gene and allele nomenclature (Section 3) (e.g., for *Homo sapiens* [2,4]) (Figure 30),
(2) IMGT/DomainGapAlign results [58,79,80],
(3) IMGT Collier de Perles [47,48,49,50] (on two layers with hydrogen bonds for V and C) created by the IMGT/Collier-de-Perles tool [51],
(4) Contact analysis between a pair of domains or between a domain and a ligand (Figure 31) (paratope/epitope description of IG/antigen (Figure 32) and TR/pMH complexes) [57,58,59],
(5) Renumbered IMGT files [57,58,59],
(6) IMGT numbering comparison [57,58,59].

In July 2020, IMGT/3Dstructure-DB contained 3985 IG structure entries (including 2152 IG/Ag complexes).

#### 5.4.2. IMGT/2Dstructure-DB

IMGT/2Dstructure-DB was created as an extension of IMGT/3Dstructure-DB [57,58,59] to describe and analyse amino acid (AA) sequences of antibodies for which no 3D structures were available. IMGT/2Dstructure-DB uses the IMGT/3Dstructure-DB informatics frame and interface, which allow one to analyse, manage, and query IG, TR, and MH, as well as other IgSF and MhSF and engineered proteins (FPIA, CPCA), as polymeric receptors made of several chains, in contrast to the IMGT/LIGM-DB sequence database that analyses and manages sequences individually [54,55]. The AA sequences are analyzed with the IMGT^®^ criteria of standardized identification [30,31], description [32,33,34,35], nomenclature [37,38] and numerotation [39,40,41,42,43,44,45,46].

The current IMGT/2Dstructure-DB entries include 336 AA sequences of antibodies from Kabat [118] (those for which there were no available nucleotide sequences), and AA sequences of mAb and FPIA from IMGT/mAb-DB [60] and the WHO-INN programme [86,87]. Queries can be made on an individual entry, using the Entry ID or the Molecule name. Thus a ‘trastuzumab’ query in ‘Molecule name’ allows to retrieve 18 results: five INN from IMGT/2Dstructure-DB, and thirteen 3D structures (of which “1nz8”) from IMGT/3Dstructure-DB. The same query interface is used for IMGT/2Dstructure-DB and IMGT/3Dstructure-DB.

The IMGT/2Dstructure-DB cards provide standardized IMGT information on chains and domains and IMGT Colliers de Perles on one or two layers, identical to that provided for the sequence analysis in IMGT/3Dstructure -DB, however the information on experimental structural data (hydrogene bonds in IMGT Collier de Perles on two layers, Contact analysis) is only available in the corresponding IMGT/3Dstructure-DB cards, if the antibodies have been cristallised.

#### 5.4.3. IMGT/mAb-DB

IMGT/mAb-DB [60], has been developed to provide an easy access to therapeutic antibody amino acid sequences (links to IMGT/2Dstructure-DB) and structures (links to IMGT/3Dstructure-DB, if 3D structures are available). IMGT/mAb-DB data include monoclonal antibodies (mAb, INN suffix -mab, being defined by the presence of at least an IG variable domain) and fusion proteins for immune applications (FPIA, for example, a receptor or membrane ligand fused to a Fc) from the WHO INN programme [86,87]. This database also includes a few composite proteins for clinical applications (CPCA) (e.g., protein or peptide fused to a Fc for only increasing their half-life; INN prefix ef- recently adopted for these CPCA) and some related proteins of the immune system (RPI) used, unmodified, for clinical applications.

## 6. Using the IMGT Numbering for V and C-Domain for Antibody Description and Engineering

### 6.1. Antibody V-DOMAIN Humanization by IMGT-CDR Grafting

#### 6.1.1. CDR-IMGT Delimitation for Grafting

For many years, the main source of specific monoclonal antibodies [301] has been from mouse or rat species, owing to the difficulty of obtaining human monoclonal antibodies by the hybridoma methodology. The objective of antibody humanization has been to graft at the DNA level the CDR of an antibody V domain, from mouse (or other species) and of a given specificity, onto the V domain framework of a human antibody, thus preserving the specificity of the original (murine or other species) antibody while decreasing its immunogenicity [302]. The Contact analysis of IG/Ag complexes in IMGT/3Dstructure-DB [57,58,59] and their analysis [303] demonstrate the preponderance of the CDR-IMGT amino acids in the paratope [298] (Figure 31; Figure 32). IMGT/DomainGapAlign [58,79,80] has become the IMGT reference tool for antibody humanization design based on CDR grafting. Indeed, it precisely defines the CDR-IMGT to be grafted and helps selecting the most appropriate human FR-IMGT by providing the alignment of the amino acid sequences between the mouse (or other species) and the closest human V-DOMAIN [90,91,92,93,94,95,96,97,98,99,100] (IMGT^®^ http://www.imgt.org, The IMGT Biotechnology page > Antibody humanization).

Analyses performed on humanized therapeutic antibodies underline the importance of a correct delimitation of the CDR and FR. As an example, two amino acid changes were required in the first version of the humanized VH of alemtuzumab, in order to restore the specificity and affinity of the original rat antibody. The positions of these amino acid changes (S28 > F and S35 > F) are now known to be located in the CDR1-IMGT and should have been directly grafted, but at the time of this mAb humanization they were considered as belonging to the FR according to the Kabat numbering [118]. In contrast, positions 66-74 were, at the same time, considered as belonging to the CDR according to the Kabat numbering, whereas they clearly belong to the FR2-IMGT and the corresponding sequence should have been ‘human’ instead of being grafted from the ‘rat’ sequence (IMGT^®^ http://www.imgt.org, The IMGT Biotechnology page > Antibody humanization > Alemtuzumab).

#### 6.1.2. Amino Acid Interactions between FR-IMGT and CDR-IMGT

IMGT Colliers de Perles from crystallized 3D structures in IMGT/3Dstructure-DB [57,58,59] highlight two conserved hydrogen bonds between FR-IMGT and CDR-IMGT positions: FR2-IMGT 39 with CDR2-IMGT 56 (or 57) and FR2-IMGT 40 with CDR3-IMGT 105 (Figure 21). Antibody engineering and humanization should therefore preserve these bondings which stabilize the loops. It is also worthwhile to note that, in VH CDR3, the stem of the CDR3 loop is stabilized by a conserved salt bridge between R106 (arginine contributed by the 3′V-REGION) and D116 (aspartate contributed by the 5′J-REGION of the *Homo sapiens* IGHJ2, IGHJ3, IGHJ4, IGHJ5 or IGHJ6) (IMGT^®^ http://www.imgt.org, IMGT Repertoire (IG and TR) > 2. Proteins and alleles > 2. Alignments of alleles > IGHJ > Mammalia > human (*Homo sapiens*)).

### 6.2. Only-Heavy-Chain Antibodies

#### 6.2.1. Dromedary IgG2 and IgG3

The dromedary or Arabian camel (*Camelus dromedarius*) IGHV3 genes belong to two sets based on four AA differences which have been linked to two antibody formats expressed in Camelidae: the conventional IG (H2L2) and the “only-heavy-chain” IG (H2, i.e., no light chain and only two identical H-gamma chains lacking CH1) [304] (IMGT^®^ http://www.imgt.org, IMGT Repertoire (IG and TR) > 2. Proteins and alleles > 1. Protein displays > V-REGION > IGHV > Arabian camel (*Camelus dromedarius*)). The AA differences characteristic of each set are located at four FR2-IMGT positions, 42, 49, 50 and 52 (42 in the C strand, 49, 50 and 52 in the C’ strand), and belong to the [GFCC’C”] sheet at the hydrophobic VH-VL interface in conventional antibodies of Camelidae as well as of any vertebrate species [1] whereas, in camelid ‘only-heavy-chain’ antibodies (no light chains, and therefore no VL), these positions are exposed to the environment with, through evolution, a selection of hydrophilic amino acids [305].

**Figure 33:**
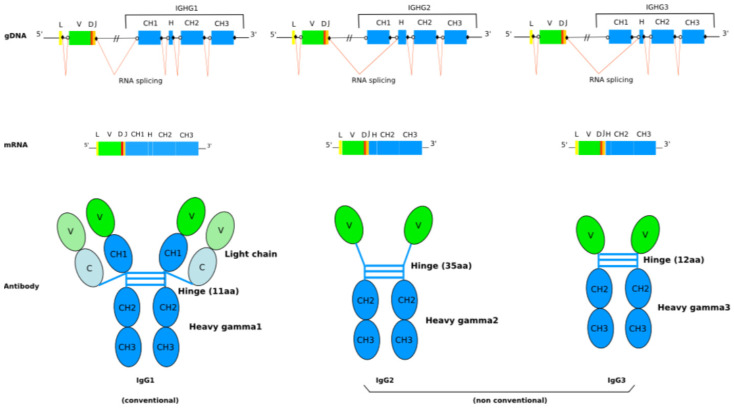
Characteristics of the immunoglobulin synthesis in Camelidae. The Arabian camel (*Camelus dromedarius*) and the llama (*Lama glama*) have three IgG subclasses: the conventional IgG1 and the non-conventional IgG2 and IgG3. IgG2 and IgG3 are characterized by the absence of light chain. This lack of light chain is itself a consequence of the absence of the CH1 domain in the H-gamma 2 and H-gamma 3 chains, due to a splicing defect of the CH1 exon (non canonical DONOR-SPLICE: nat instead of ngt). (With permission from M-P. Lefranc and G. Lefranc, LIGM, Founders and Authors of IMGT^®^, the international ImMunoGeneTics information system^®^, http://www.imgt.org).

The first set of IGHV3 genes is expressed in conventional tetrameric IgG1 that constitute 25% of circulating antibodies. The second set is expressed in ‘only-heavy-chain’ IgG2 and IgG3 that constitute 75% of the circulating antibodies [304]. The respective H-gamma2 and H-gamma3 chains are both characterized by the absence of the CH1 domain owing to a splicing defect [306]. It is the absence of CH1 which is responsible for the lack of association of the light chains (see 6.1.2) (Figure 33) (IMGT^®^ http://www.imgt.org, IMGT Repertoire (IG and TR) > 1. Locus and Genes > 7. Gene tables; ibid., IMGT Biotechnology page > Characteristics of the camelidae (camel, llama) antibody synthesis). Only-heavy-chain antibodies is a feature of the Camelidae IG as they have also been found in the Bactrian camel (*Camelus bactrianus*) of Central Asia and in the llama (*Lama glama*) and alpaca (*Vicugna pacos*) of South America. The genetic event (splicing defect) responsible for the lack of CH1 occurred in their common ancestor before the radiation between the ‘camelini’ and ‘lamini’, dating approximately 11 million years (Ma) ago.

The V domains of Camelidae ‘only-heavy-chain’ antibodies have characteristics for potential pharmaceutical applications (e.g., specificities with binding to protein clefts for those with extended CDR3, easy production and selection of single-domain format with novel specificities). They are designated as VH_H_ domain when they have to be distinguished from conventional VH (the V sequence criterion is based on the four AA at positions 42, 49, 50 and 52, particularly 49 and 50, with E49 (or Q49) and R50 in VH_H_, and G49 and L50 (or P50) in VH. A more complete knowledge of the germline genes is required to identify somatic mutations from genetic polymorphisms, for the other positions. Most llama VH_H_ have normal or long CDR3-IMGT and, as expected from Arabian camel, have E49 (or Q49) and R50 (i.e., llama anti-TNF 5m2i [8.8.16] and 5m2m [12.8.18] [307], or llama anti-HIV 5hm1 [8.7.14] [308]). However some llama VH_H_, defined as having no paired VL, were found unexpectedly to have the conventional G49 and L50, and to be characterized by a short CDR3-IMGT of 8 amino acids and Arg R118 (instead of J-TRP W118) (i.e., 5m2j [8.8.8] [307]). The selection of the J-REGION R118 (instead of W118, in the IGHJ 118-121 ‘W-G-X-G’ motif, G strand of the V domain) and that of the V-REGION R50 (instead of L50 or P50, in the IGHV, C’ strand of the V domain) represent therefore two different evolutionary paths which make the G-F-C-C’ layer of the VH_H_ more hydrophilic (Figure 21). The term ‘nanobody’ initially used for describing a single-domain format antibody is not equivalent to VH_H_, as it has been used for V domains other than VH_H_ and for constructs containing more than one V domain and is a registered mark (VH and/or VH_H_) (e.g., caplacizumab, ozoralizumab) (IMGT^®^ http://www.imgt.org, IMGT/mAb-DB > caplacizumab; ibid. ozoralizumab).

#### 6.2.2. Human Heavy Chain Diseases (HCD)

The camelidae ‘only-heavy-chain’ antibodies synthesis is remarkably reminiscent of what is observed in human heavy chain diseases (HCD). These proliferative disorders of B lymphoid cells produce truncated monoclonal immunoglobulin heavy chains which lack associated light chains. In most HCD, the absence of the heavy chain CH1 domain by deletion or splicing defect may be responsible for the lack of assembly of the light chain [309]. Similar observations have also been reported in mouse variants [309] (IMGT^®^ http://www.imgt.org, IMGT Education > Pathology of the immune system > Molecular defects in Immunoglobulin Heavy Chain Diseases (HCD); ibid., IMGT Lexique > Heavy Chain Diseases (HCD)).

#### 6.2.3. Nurse Shark IgN

A convergence mechanism in evolution is observed in nurse shark *Ginglymostoma cirratum* (‘Gincir’ in the IMGT 6-letter abbreviaton code for species) IgN antibodies (or IgNAR, ‘immunoglobulin new antigen receptor’) [310] which are ‘only-heavy-chain’ antibodies (H2 homodimeric H-nu chains without CH1, and no associated light chains). The IGHV genes expressed in the Gincir H-nu chains belong to the IGHV2 subgroup and are characterized by the absence of the CDR2-IMGT owing to a deletion that encompasses position 54 to 67. The Gincir IGH genes are organized in duplicated cassettes, and those that express IgN comprise Gincir IGHV2 subgroup genes and an IGHN constant gene (IMGT^®^ http://www.imgt.org, IMGT Repertoire (IG and TR) > 2. Proteins and alleles > 1. Protein displays > IGHV > nurse shark (*Ginglymostoma cirratum*), ibid., IGHC; ibid., > 1. Locus and genes > 7. Gene tables > IGHV > Chondrichthyes > nurse shark (*Ginglymostoma cirratum*), ibid., IGHC).

### 6.3. Contact Analysis of TR-Mimic Antibodies and TR

**Figure 34:**
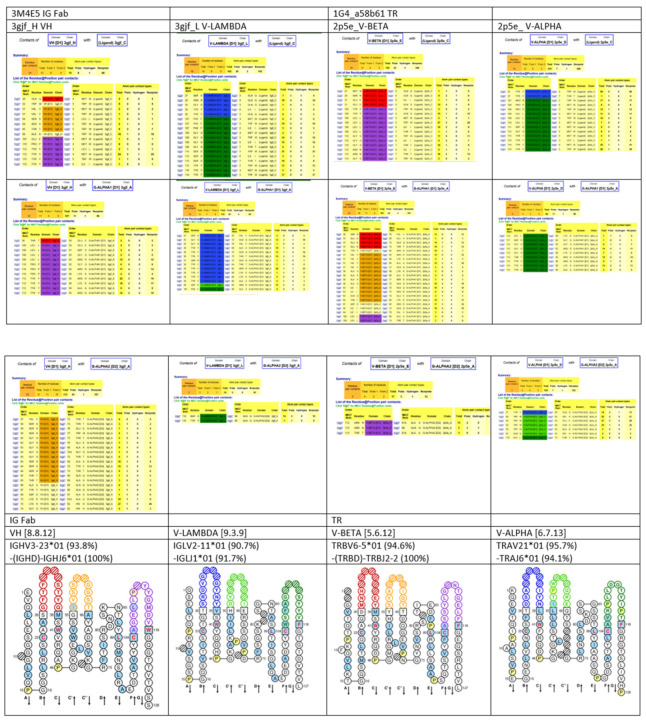

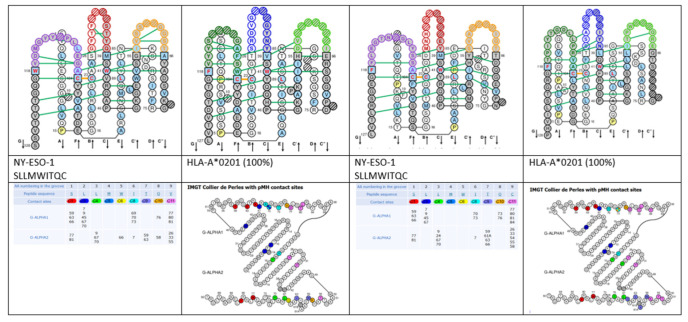
Contact analysis and IMGT Collier de Perles of a TR-mimic antibody and of a TR targeting peptide-HLA. VH and V-LAMBDA of the IG Fab 3M4E5 and V-BETA and V-ALPHA of the TR 1G4_a58b61 target the NY-ESO-1 peptide presented by HLA-A*02:01 [311,312]. From top to bottom are shown: 1. contacts with the ligand, 2. contacts with G-ALPHA1, 3. contacts with G-ALPHA2, 4. IMGT Collier de Perles on one layer, 5. IMGT Collier de Perles on two layers with hydrogen bonds, 6. contacts of the NY-ESO-1 peptide SLLMWITQC with the MH1 HLA-A*02:01 groove [313,314,315]. The CDR1-IMGT, CDR2-IMGT and CDR3-IMGT are represented in red, orange and purple for the IG VH and the TR V-BETA, and in blue, green, bluegreen for the IG VL, here V-LAMBDA and for the TR V-ALPHA. Three-dimensional structure PDB codes are 3gjf, for Fab 3M4E5 (IG/pMH1), and 2p5e, for 1G4_a58b61 (TR/pMH1) (IMGT^®^ http://www.imgt.org, IMGT/3Dstructure-DB). (With permission from M-P. Lefranc and G. Lefranc, LIGM, Founders and Authors of IMGT^®^, the international ImMunoGeneTics information system^®^, http://www.imgt.org).

The IMGT unique numbering has recently allowed the contact analysis comparison between an antibody and a T cell receptor, with a same ligand [311]. Both immunoglobulin, IG Fab 3M4E5, a TR-mimic antibody, and the T cell receptor, TR 1G4_a58b61, target the NY-ESO-1 peptide SLLMWITQC presented by HLA-A*02:01 [312] (Figure 34). IMGT Colliers de Perles on one and two layers and contact analyses of the IG/pMH1 (3gjf, for Fab 3M4E5) and TR/pMH1 (2p5e, for 1G4_a58b61) complexes [311] are available in IMGT/3Dstructure-DB, based on the unique numbering V-DOMAIN for IG and TR [40,41,42], and the IMGT unique numbering for G-DOMAIN for MH [43]. They allow to visualize the features and differences in the antigen recognition by an antibody and a TR targeting the same p/MH antigen (Figure 34). The contacts of the NY-ESO-1 peptide SLLMWITQC with the MH1 HLA-A*02:01 groove are similar in the two peptide-HLA complexes as expected [313,314,315].

### 6.4. Antibody C-Domain Post-Translational Modifications, Engineering and Allotypes

The constant region of the IG heavy chain is made of several CH domains, which are analysed and described in IMGT^®^ using the IMGT unique numbering [42,44,45,46]. This allows a universal standardized comparison of sequences and 3D structures between C domains of any chain, any receptor and any species. Examples of post-translational modifications (glycosylations), effector properties and engineering at the C-DOMAIN level are given in the following subsections.

#### 6.4.1. N-Linked Glycosylation Sites CH2 N84.4

**Figure 35:**
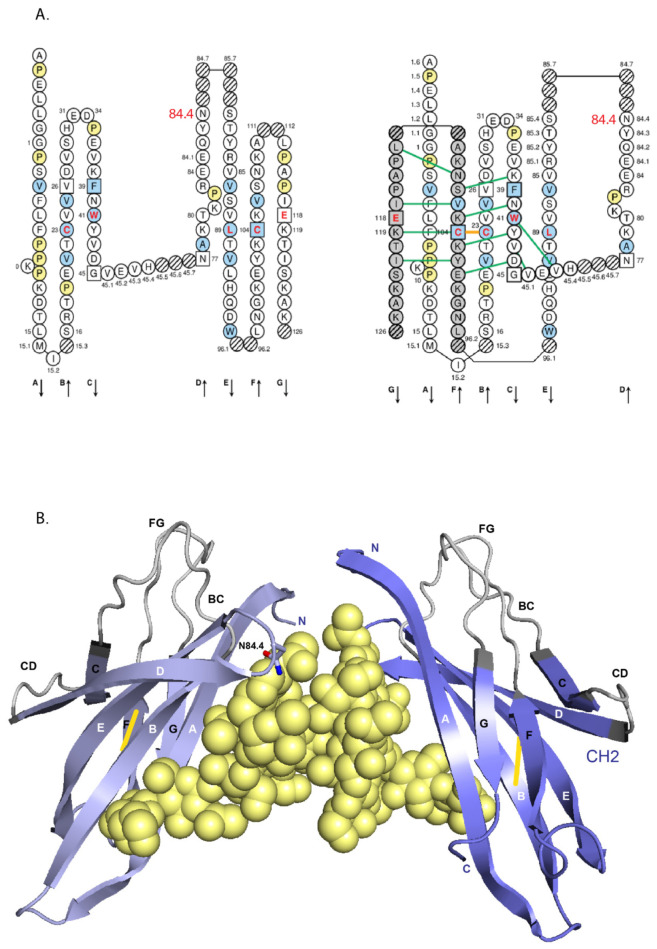
*Homo sapiens* IGHG1 CH2 and N-linked glycosylation site N84.4 [94]. (**A**) IMGT Collier de Perles of IGHG1 CH2 on one layer (on the left) and on two layers with hydrogen bonds (on the right). The N84.4 is at the DE turn. The identifier of the chain to which the domain belongs is 1hzh_H. The IMGT Colliers de Perles on two layers show, in the forefront, the GFC strands and, in the back, the ABED strands, linked by the CD transversal strand. The IMGT Colliers de Perles were generated by the IMGT/Collier-de-Perles tool [51] integrated in IMGT/3Dstructure-DB [57,58,59]. Hydrogen bonds (green lines) and disulfide bond between C23 and C104 (orange line) were automatically added from the experimental structural data). Amino acids are shown in the one-letter abbreviation. Positions at which hydrophobic amino acids (hydropathy index with positive value: I, V, L, F, C, M, A) and tryptophan (W) are found in more than 50% of analysed sequences are shown in blue. All proline (P) are shown in yellow. Hatched circles correspond to missing positions according to the IMGT unique numbering for C domain [42]. Arrows indicate the direction of the beta strands and their designations in 3D structures. Anchors (26, 39, 45, 77, 104, 118) are shown in squares. (**B**) 3D structure of the IGHG1 CH2 dimer with the two carbohydrate chains. The N84.4 at the DE turn is shown on the CH2 on the left whereas the N84.4 of the CH2 on the right is hidden behind the carbohydrates. The ribbon representation of the 3D structures was obtained using PyMOL (http://www.pymol.org). The identifiers of the H-gamma1 chains to which the CH2 domains belong are 1hzh_H and 1hzh_K (IMGT^®^ http://www.imgt.org, IMGT/3Dstructure-DB). (With permission from M-P. Lefranc and G. Lefranc, LIGM, Founders and Authors of IMGT^®^, the international ImMunoGeneTics information system^®^, http://www.imgt.org).

The N-linked glycosylation site present in the CH2 domain of the constant region of the four human IG H-gamma chains is located at CH2 N84.4. As shown in the IMGT Collier de Perles, this asparagine is localized at the DE turn (Figure 35).

**Figure 36:**
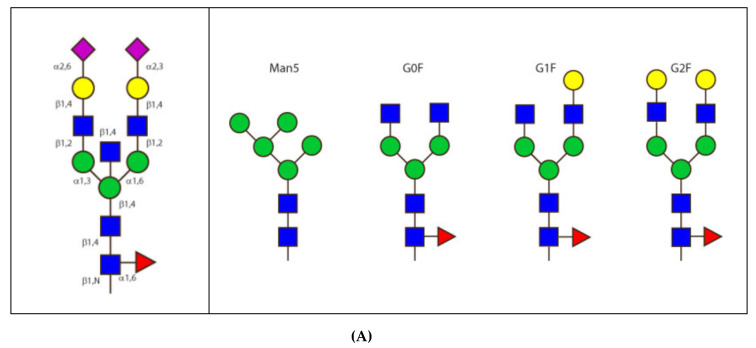

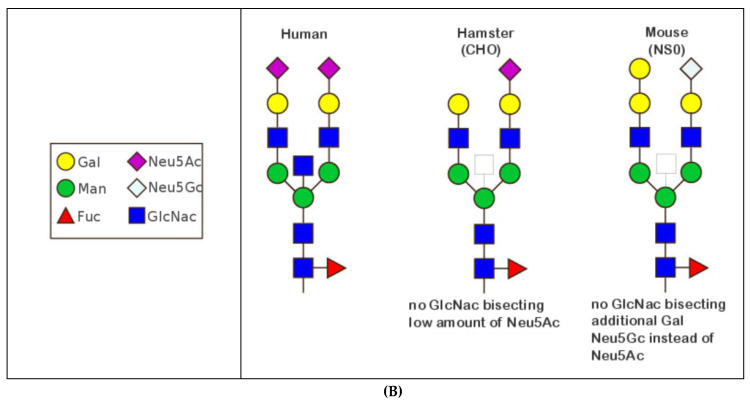
(**A**). Largest N-linked oligosaccharide structure found in human IgG (left panel) and most abundant glycans found in mAb biopharmaceuticals (right panel). (**B**). Glycosylation patterns of recombinant antibodies produced in human, CHO and NS0 cells. Symbol Nomenclature for Glycans (SNFG) is according to the NCBI Glycans page https://www.ncbi.nlm.nih.gov/glycans/snfg.html. Gal: galactose, Man: mannose, Fuc: fucose, GlcNac: N-acetylglucosamine, Neu5Ac: N-acetylneuraminic acid and Neu5Gc: N-glycolylneuraminic acid. The absence of GlcNac bisecting, in glycans of recombinant antibodies produced in hamster (CHO) and mouse (NS0) cells, is marked by a white square (IMGT^®^ http://www.imgt.org, IMGT Lexique > Immunoglobulin (IG) or antibody glycosylation). (With permission from M-P. Lefranc and G. Lefranc, LIGM, Founders and Authors of IMGT^®^, the international ImMunoGeneTics information system^®^, http://www.imgt.org).

The human glycans are mainly classified as ‘biantennary complex’ structure with a core fucose (Fuc) and are often terminated with N-acetylneuraminic acid (Neu5Ac), a sialic acid (Figure 36). The largest N-linked oligosaccharide structure found in human IgG is shown in Figure 36A (left panel). The conserved heptasaccharide core is composed of two N-acetylglucosamine (GlcNAc), three mannose (Man) and two other GlcNAc residues that are β-1,2 linked to α-6 Man and α-3 Man, forming two arms. The bisecting N-acetylglucosamine (GlcNac, NAG) represents around 10% of human IgG glycoforms. The four most abundant glycans in mAb biopharmaceuticals are shown in Figure 36A (right panel). The Fc oligosaccharides are terminated by zero, one or two galactoses and are called G0, G1 or G2, respectively. For G1F, Gal can be on the α1,3-arm or on the α1,6-arm. Additional fucose (Fuc), galactose (Gal), N-acetylneuraminic acid (Neu5Ac) and N-glycolylneuraminic acid (Neu5Gc) residues may be present or not, particularly depending on the expression system. Mammalian cell expression systems are the favorite methods for the commercial production of monoclonal antibodies because their protein glycosylation machinery closely resembles that in human. The current marketed antibodies are mainly expressed in CHO (Chinese Hamster Ovary), SP2/0 (mouse myeloma cells), NS0 (Non-Secreting mouse myeloma cells) and hybridomas (Figure 36B).

The graphical representation of the 36 possible structures of IG N-glycans expressed in human cells are available in the IMGT Lexique, with correspondence between the ‘GA’ nomenclature, based on the number of galactose (G) (0, 1 or 2) and sialic acid (A) (0, 1 or 2) at the terminal ends of the arms of the IG N-glycans, and the ‘HN’ nomenclature based on the number of carbohydrates in the glycan composition (H: number of hexoses, N: number of N-acetyl glucosamine), with, if present, S number of sialic acids (IMGT^®^ http://www.imgt.org, IMGT Lexique > Immunoglobulin (IG) or antibody glycosylation).

#### 6.4.2. Knobs-into-Holes

**Figure 37:**
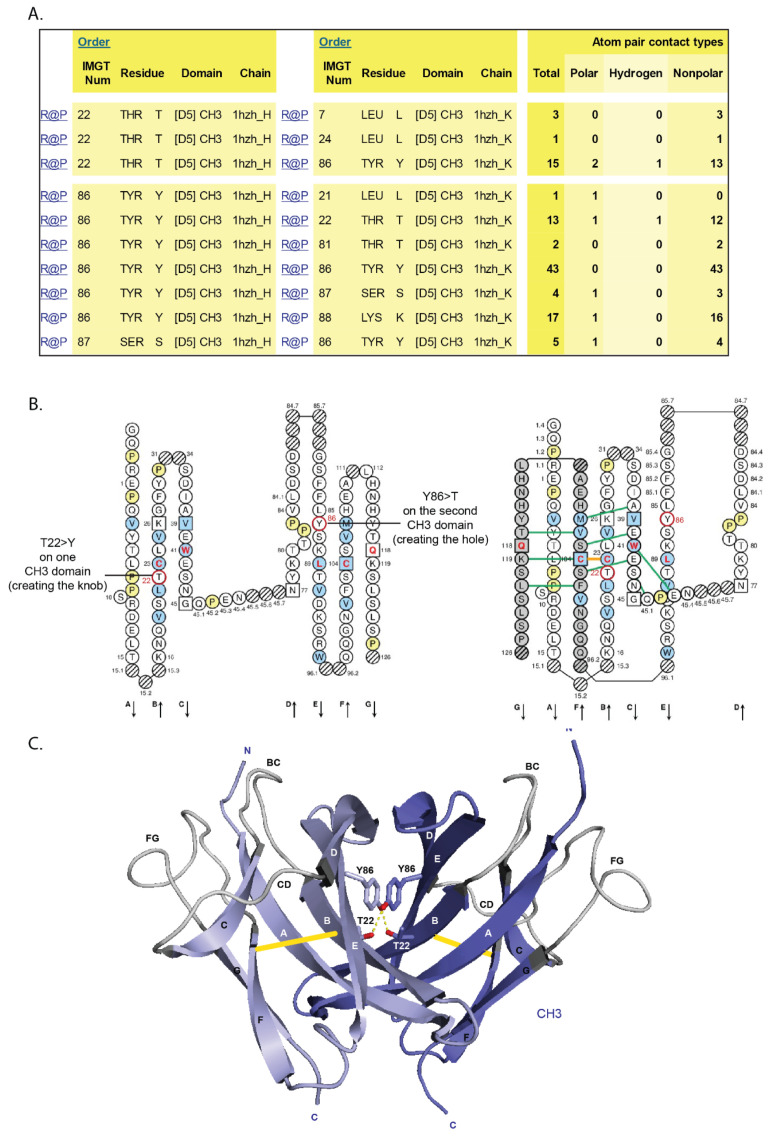
*Homo sapiens* IGHG1 CH3 and knobs-into-holes positions [94]. (**A**) Contact analysis of threonine T22 and tyrosine Y86 at the interface between the two CH3 domains of the gamma1 chains. T22 and Y86 are the two positions involved in the knobs-and-holes. (**B**) IMGT Colliers de Perles of *Homo sapiens* IGHG1 CH3 on one layer (on the left) and on two layers with hydrogen bonds (on the right). T22 (in strand B) and Y86 (in strand E) are highlighted. The IMGT Colliers de Perles on two layers show, in the forefront, the GFC strands and, in the back, the ABED strands, linked by the CD transversal strand. The [ABED] sheets are at the interface between the two CH3 domains. The IMGT Colliers de Perles were generated by the IMGT/Collier-de-Perles tool [51] integrated in IMGT/3Dstructure-DB [57,58,59]. Hydrogen bonds (green lines) and disulfide bond between C23 and C104 (orange line) were automatically added from the experimental structural data. Amino acids are shown in the one-letter abbreviation. Positions at which hydrophobic amino acids (hydropathy index with positive value: I, V, L, F, C, M, A) and tryptophan (W) are found in more than 50% of analysed sequences are shown in blue. All proline (P) are shown in yellow. Hatched circles correspond to missing positions according to the IMGT unique numbering for C domain [42]. Arrows indicate the direction of the beta strands and their designations in 3D structures. Anchors (26, 39, 45, 77, 104, 118) are shown in squares. (**C**) 3D structure of the CH3 dimer showing the two amino acids T22 and Y86 involved in the knobs-and-holes amino acid changes (T22 > Y on one CH3 domain, Y86 > T on the other). The identifiers of the gamma1 chains to which the CH3 domains belong are 1hzh_H and 1hzh_K (IMGT^®^ http://www.imgt.org, IMGT/3Dstructure-DB). (With permission from M-P. Lefranc and G. Lefranc, LIGM, Founders and Authors of IMGT^®^, the international ImMunoGeneTics information system^®^, http://www.imgt.org).

The knobs-into-holes methodology has been proposed for obtaining bispecific antibodies [309]. The aim is to increase interactions between the CH3 domain of two H-gamma1 chains that belong to antibodies with a different specificity to obtain bispecific antibodies. The two amino acids CH3 T22 (B strand) and Y86 (E strand) selected for changes belong to the [ABED] sheet, at the interface of the two *Homo sapiens* IGHG1 CH3 domains (Figure 37). Interactions of these two amino acids are described in ‘Contact analysis’ in IMGT/3Dstructure-DB [57,58,59] (Figure 37A). The knobs-into-holes methodology consists into an amino acid change on one CH3 domain (here, T22 > Y) that creates a knob, and another amino acid change on the other CH3 domain (here, Y86 > T) that creates a hole, thus favoring increased interactions between the CH3 of the two H-gamma1 chains at both positions 22 and 86 [316] (IMGT^®^ http://www.imgt.org, The IMGT Biotechnology page > Bispecific antibodies > Knobs-into-holes amino acid changes). (With permission from M-P. Lefranc and G. Lefranc, LIGM, Founders and Authors of IMGT^®^, the international ImMunoGeneTics information system^®^, http://www.imgt.org).

#### 6.4.3. Interface Ball-and-Socket-Like Joints

**Figure 38:**
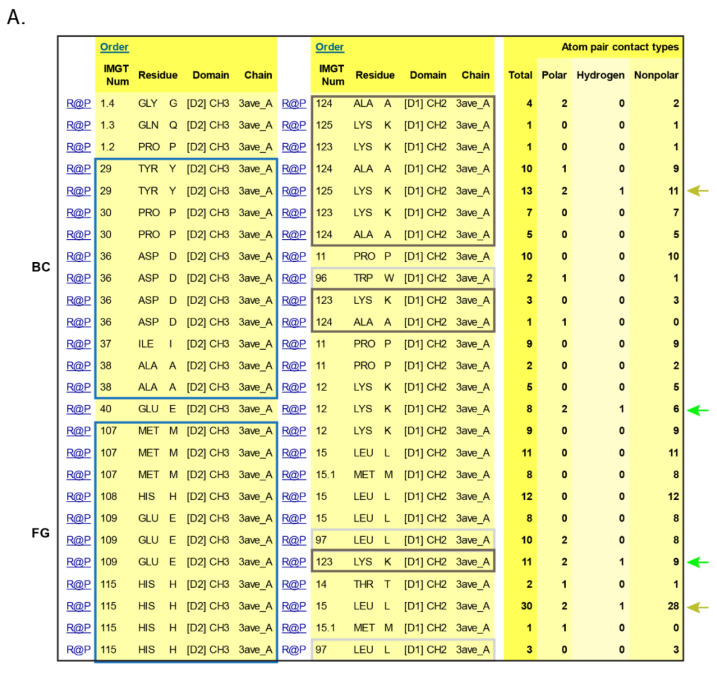

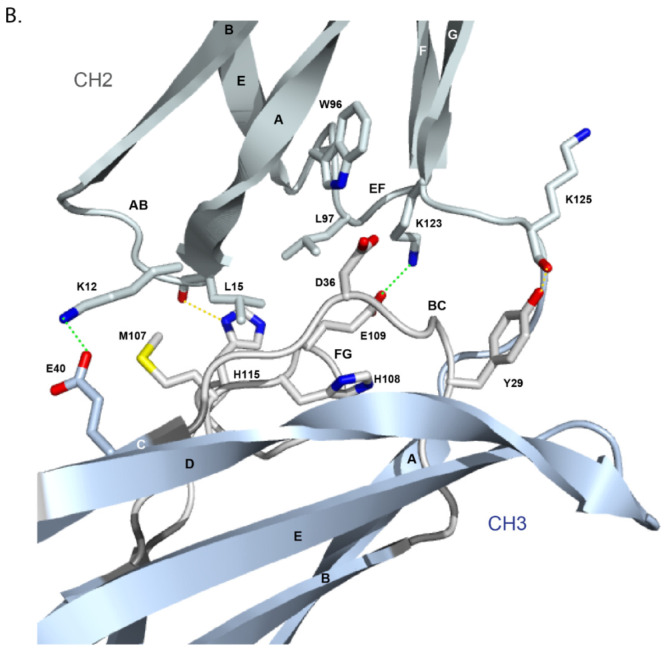
Contact analysis and ball-and-socket interface between *Homo sapiens* IGHG1 CH2 and CH3 [94]. (**A**) Contact analysis between the CH2 and CH3 domains of the Fc gamma1 (from IMGT/3Dstructure-DB, 3ave_A). The amino acids of the CH3 BC and FG loops (left column) and those of the CH2 G strand (right column) are shown in rectangles. CH2 96 and 97 correspond to the EF turn whereas other positions are from the A strand or AB turn. Arrows indicates the two hydrogen bonds (orange on line) and the two salt bridges (green online) mentioned in the text [312]. (**B**) The ball-and-socket-joint of the IGHG1 CH2-CH3 interface [312] is shown using the IMGT numbering, with the ball (L15) and the socket (M107, H108, E109 and H115). The interface is stabilized by two hydrogen bonds involving on CH2, L15 (O) and K125 (O) that bind on CH3, H115 (ND1) and Y29 (OH), respectively, and by two salt bridges involving on CH2, K12 (A strand) and K123 (G strand) that interact on CH3 with E40 (C strand) and E109 (FG loop), respectively. The identifier of the gamma1 chain to which the CH2 and CH3 domains belong is 3ave_A (IMGT^®^ http://www.imgt.org, IMGT/3Dstructure-DB). (With permission from M-P. Lefranc and G. Lefranc, LIGM, Founders and Authors of IMGT^®^, the international ImMunoGeneTics information system^®^, http://www.imgt.org).

The comparison of the interface between the CH2 and CH3 domains from 3D structures of *Homo sapiens* IGHG2 Fc with the interface in 3D structures of IGHG1 Fc revealed that in all Fc of gamma chains the movement of the CH2 results from a pivoting around a highly conserved ball-and-socket-like joint [317]. Using the IMGT unique numbering for C-domain, the CH2 L15 side chain (last position of the A strand, next to the AB turn) (the ‘ball’) interacts with a pocket (the ‘socket’) formed by CH3 M107, H108, E109 and H115 (FG loop) (Figure 38). The interface is stabilized by two hydrogen bonds: CH2 L15 (O) and CH3 H115 (ND1), CH2 K125 (O) and CH3 Y29 (OH), and by two salt bridges: CH2 K12 (A strand) and CH3 E40 (C strand), CH2 K123 (G strand) and CH3 E109 (FG) (Figure 38). These amino acids are well conserved between the *Homo sapiens* gamma isotypes and the IGHG genes and alleles except for IGHG3 H115 that shows a polymorphism associated to different G3m allotypes [83]. This ball-and-socket-like joint is a structural feature similar but reversed to that previously described at the VH and CH1 domain interface [318], in which the VH L12, T125 and S127 form the ‘socket’ whereas the CH1 F29 and P30 form the ‘ball’ (IMGT^®^ http://www.imgt.org, IMGT Repertoire > 2. Proteins and alleles > 1. Protein displays > C-DOMAIN with CHS, M and HINGE regions; ibid., IMGT Repertoire > 2. Proteins and alleles > 2. Alignments of alleles > IGHC; ibid., IMGT/3Dstructure-DB > query on Fab).

#### 6.4.4. IGHG Alleles and Gm Allotypes

Allotypes are polymorphic markers of an IG subclass that correspond to amino acid changes and are detected serologically by antibody reagents [83]. In therapeutic antibodies (human, humanized or chimeric), allotypes may represent potential immunogenic epitopes [82], as demonstrated by the presence of antibodies in individuals immunized against these allotypes [83]. For the H-gamma chains, the allotypes are designated as Gm (for gamma marker), and for the H-gamma1 chains as G1m [83]. The allotypes G1m, G2m and G3m are carried by the constant region of the H-gamma1, H-gamma2 and H-gamma3 chains, encoded by the IGHG1, IGHG2 and IGHG3 genes, respectively.

The H-gamma1 chains may express four G1m alleles (combinations of G1m allotypes): G1m3, G1m3,1, G1m17,1, and G1m17,1,2 (and in Negroid populations three additional G1m alleles, Gm17,1,27, Gm17,1,28 and Gm17,1,27,28) [83]. The correspondence between the G1m alleles and IGHG1 alleles is shown in Table 14.

**Figure 39:**
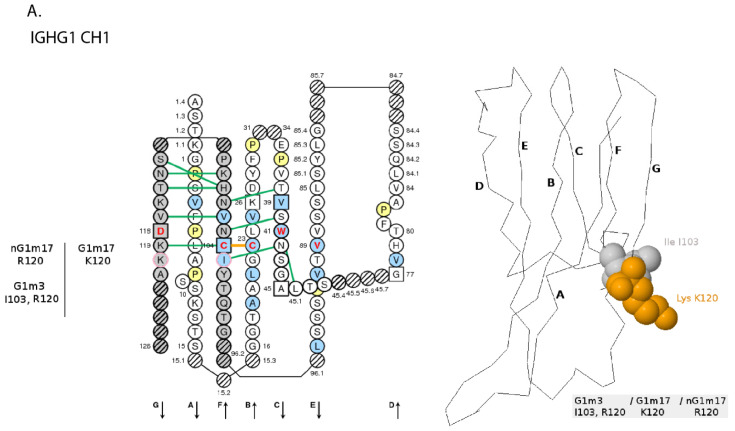

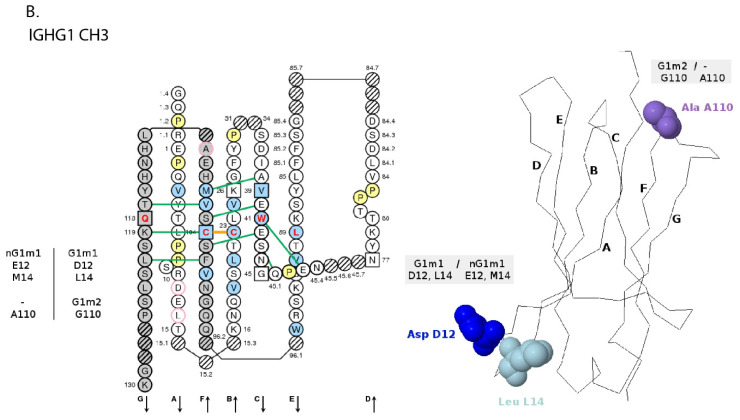
*Homo sapiens* IGHG1 and G1m allotypes [83,94]. (**A**) IGHG1 CH1. The IMGT Collier de Perles of IGHG1 CH1 on two layers is shown with hydrogen bonds, as green lines online (on the left). K120 (strand G) corresponds to the G1m17 allotype [83]. I103 (strand F) is specific of the H-gamma1 chain isotype. The simultaneous presence of R120 and I103 corresponds to the expression of the G1m3 allotype (not shown) [83]. For isotypes other than H-gamma1, R120 corresponds to the expression of the nG1m17 isoallotype (not shown) [83]. The 3D structure of the CH1 (on the right) shows K120 (G1m17) and the H-gamma1 specific I103 [83]. (**B**) IGHG1 CH3. The IMGT Collier de Perles of IGHG1 CH3 on two layers is shown with hydrogen bonds, as green lines online (on the left). D12 and L14 (strand A) correspond to the G1m1 allotype, whereas E12 and M14 (not shown) correspond to the nG1m1 isoallotype [83]. G110 (not shown) corresponds to G1m2, whereas A110 (here) does not correspond to any allotype [83]. G129 and K130 represent the CHS in secreted IG. The 3D structure of the CH3 (on the right) shows the positions 12 and 14 of the G1m1/nG1m1 allotype, and position 110 of the G1m2/- allotype. The CH1 and CH3 domains are from the b12 antibody. The b12 H-gamma1 chain is encoded by the IGHG1*01 allele and expresses the G1m17,1 allotypes, based on sequence analysis (CH1 K120, CH3 D12 and L14) [83]. The presence of an A (Ala) in CH1 121 of 1hzh_H is a PDB file error. It should be a V (Val) as in 1n0x_H, and as mentioned in the IMGT note of 1hzh(IMGT^®^ http://www.imgt.org, IMGT/3Dstructure-DB > Entry code (PDB): 1hzh). (With permission from M-P. Lefranc and G. Lefranc, LIGM, Founders and Authors of IMGT^®^, the international ImMunoGeneTics information system^®^, http://www.imgt.org).

Amino acids involved in the expression of G1m allotypes and localized on the CH1 and CH3 domains of H-gamma1 chains are shown in IMGT Colliers de Perles on two layers and in 3D structures (Figure 39). In the CH1, the lysine at position 120 (K120) in strand G corresponds to the G1m17 allotype [83]. The isoleucine I103 (strand F) is specific of the H-gamma1 chain isotype. If an arginine is expressed at position 120 (R120), the simultaneous presence of R120 and I103 corresponds to the expression of the G1m3 allotype [83]. For isotypes other than H-gamma1, R120 corresponds to the expression of the nG1m17 isoallotype (an isoallotype or nGm is detected by antibody reagents that identify this marker as an allotype in one IgG subclass and as an isotype for other subclasses). In the CH3, the aspartate D12 and leucine L14 (strand A) correspond to G1m1, whereas glutamate E12 and methionine M14 correspond to the nG1m1 isoallotype [83]. A glycine at position 110 corresponds to G1m2, whereas an alanine does not correspond to any allotype (G1m2-negative chain).

Trastuzumab has been engineered in order to obtain the less immunogenic (being most frequent in different populations) allotype G1m17 (CH1 K120), associated to the nG1m1 (CH3 E12, M14) [319], defined using the generic description, as IGHG1*03v, G1m3 > G1m17, nG1m1 (CH1 R120 > K, CH3 E12,M14). The G1m allotypes have been confirmed serologically [82].

The H-gamma2 chains express only one allotype G2m23. G2m23 and the H-gamma2 chains are either G2m23 or G2m. (two dots indicate that a specimen was tested and found to be negative for G2m23 [83]. G2m23 is localized on CH2. Amino acid sequence and 3D structure comparisons show that the G2m23 allotype is correlated with CH2 M45.1, whereas the absence of the allotype (G2m..) is correlated with valine V45.1 [83]. The G2m23-positive H-gamma2 chains are also characterized by the presence of threonine T92 in the CH1, whereas the G2m23-negative chains and the H-gamma chains of other IgG subclasses have proline P92 in the CH1. Being located on the CH1 domain this amino acid change is not involved in the expression of the G2m23 allotype, but owing to the strong linkage on the same chain, the CH1 T92 codon has been used for the molecular characterization of the G2m23 chains [83].

**Figure 40:**
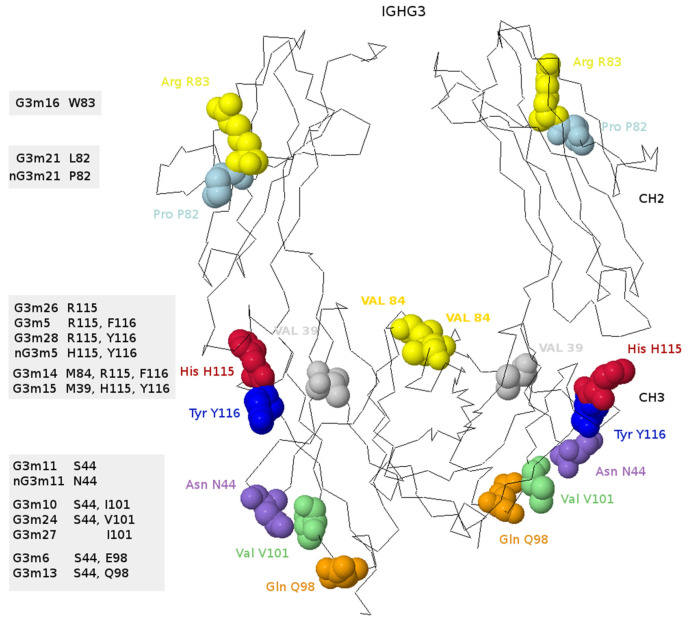
G3m allotypes localizations on the gamma3 chains [83]. (With permission from M-P. Lefranc and G. Lefranc, LIGM, Founders and Authors of IMGT^®^, the international ImMunoGeneTics information system^®^, http://www.imgt.org).

**Figure 41:**
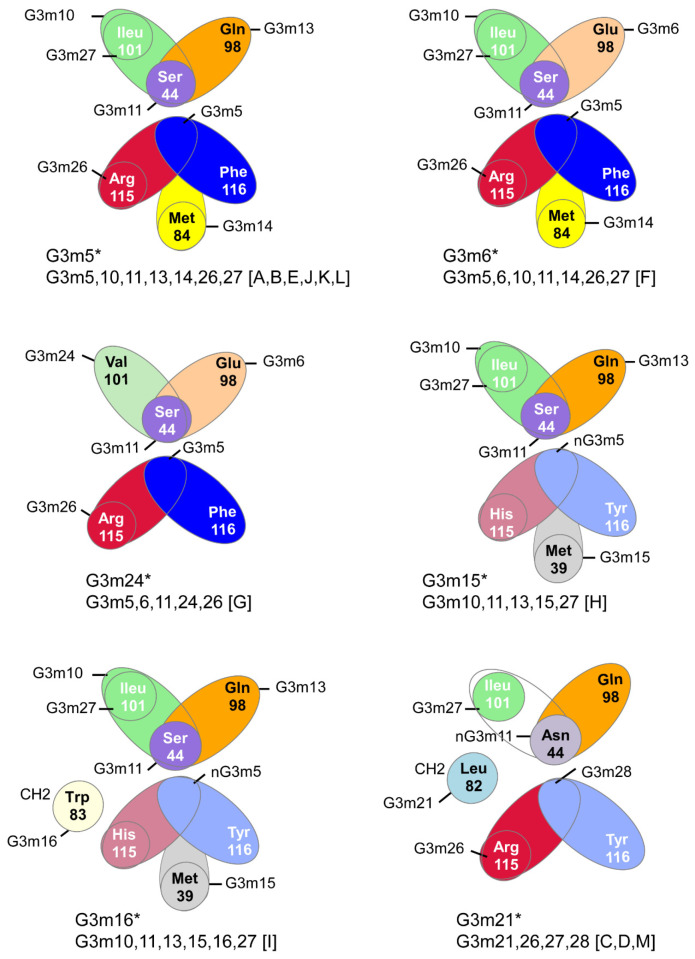
IMGT G3m allele butterfly’ representation [83]. The two mosaics on the CH3 domain are shown for each G3m allele (allotypes are described in [83]). The first mosaic around G3m11 is on the top with G3m27, G3m10/G3m24, G3m13/G3m6. The second mosaic is at the bottom with G3m26, G3m5/nG3m5/G3m28. Amino acids involved in the allotype expression and their position according to the IMGT unique numbering for C domain [42] are indicated. Two allotypes are on the CH2 domain, G3m16 (Trp W83) and G3m21 (Leu L82). Haplotypes to which the G3m alleles belong, are indicated between square brackets. (With permission from M-P. Lefranc and G. Lefranc, LIGM, Founders and Authors of IMGT^®^, the international ImMunoGeneTics information system^®^, http://www.imgt.org).

The H-gamma3 chains are the most polymorphic IG chains in humans. Thirteen G3m allotypes are characterized: G3m5, G3m6, G3m10, G3m11, G3m13, G3m14, G3m15, G3m16, G3m21, G3m24, G3m26, G3m27, G3m28 [83]. Three isoallotypes (nG3m5, nG3m11, and nG3m21) have also been characterized. Two G3m allotypes are located on the CH2 of gamma3 chains, G3m16 (W83) and G3m21 (L82), nG3m21 (P82) [83]. The other G3m allotypes form two mosaics on the CH3 [83] (Figure 40). A first mosaic includes G3m11 (S44), nG3m11 (N44), G3m10 (S44, I101)/G3m24 (S44, V101), G3m27 (I101), G3m6 (S44, E98)/G3m13 (S44, Q98). The second mosaic includes G3m 26 (R115), G3m5 (R115, F116)/nG3m5 (H115, Y116)/G3m28 (R115, Y116), G3m14 (M84, R115, F116) and G3m15 (M39, H115, Y116) [83] (Figure 40). The combination of expressed allotypes correspond to haplotypes represented in the IMGT G3m allele butterfly’ representation [83] (Figure 41).

The therapeutical antibody omodenbamab (INN L123, 2020) is an *Homo sapiens* immunoglobulin G3-kappa, anti-*Staphylococcus aureus* SpA (Staphylococcal protein A). The C region of the H-gamma3 chains is encoded by IGHG3*01, G3m5* (G3m5,10,11,13,14,26,27) with CH3 S44 (439), M84 (452), Q98 (474), I101 (477), R115 (490) and F116 (491).

#### 6.4.5. IGHG Engineered Variants and Effector Properties

Amino acids in the IGHG constant regions of the IG heavy chains are frequently engineered to modify the effector properties of the therapeutic monoclonal antibodies. Amino acids changes are engineered at positions involved in antibody-dependent cellular (ADCC), antibody-dependent cellular phagocytosis (ADCP), complement-dependent cytotoxicity (CDC), half-life increase, half-IG exchange, and B cell inhibition by coengagement of antigen and FcγR on the same cell [320,321] (IMGT^®^ http://www.imgt.org, The IMGT Biotechnology page > Amino acid positions involved in ADCC, ADCP, CDC, half-life and half-IG exchange).

The IMGT engineered variant nomenclature (Table 15) has been set up for an easier comparison between engineered antibodies [311]. The IMGT engineered variant name comprises the species, the gene name, the letter ‘v’ with a number (e.g., *Homo sapiens* IGHG1v1), and then the domain(s) with AA change(s) defined by the letter of the novel AA and position in the domain, e.g., CH2, P1.4. In Table 15, correspondence with the Eu numbering is shown between parentheses, whereas in antibody descriptions (i.e., INN proposed and recommended lists), positons between parentheses are those in the antibody chains. The IMGT engineered variants are classified by comparison with the allele *01 of the gene and, if the effects are independent on the alleles, as a reference for the description of the amino acid (AA) changes for the other alleles. In those cases, the same variant (v) number is used for any allele of the same gene in the same species.

Examples of Fc engineered variants from Table 15 [311], found frequently in therapeutical antibodies, include for ADCC and CDC reduction of IgG1, the variant G1v4: CH2 A114 (329), for ADCC and CDC reduction of IgG2, the variant G2v3: CH2 A1.2 (235), A1 (237), S2 (238), A30 (268), L92 (309), S115 (330), S116 (331), and for ADCC and CDC reduction of IgG4, the variant G4v4: CH2 A1.3 (234), A1.2 (235). Half-life increase of IgG1 is obtained with the variant G1v21: CH2 Y15.1 (252), T16 (254), E18 (256). IGHG4 half-IG exchange reduction is obtained with the variant G4v5: h P10 (228). An absence of H-gamma N-glycosylation is obtained by one of the variants affecting the CH2 N84.4, for example, G4v36: CH2 Q84.4 (297).

## 7. Conclusions

IMGT^®^ bridging genes, structures and functions provides a unique frame for three research axes: deciphering the IG and TR locus, genes and alleles in genomes of vertebrates from fish to humans, identifying clonality and exploring high-throughput repertoires with IMGT/HighV-QUEST and exploiting data from IMGT/mAb-DB, IMGT/2Dstructure-DB and IMGT/3Dstructure-DB towards targeted and customized therapeutic antibodies. Regarding the first axis, IMGT^®^ genomic annotated data are classically displayed in IMGT Repertoire Web Resources (Locus description, Locus representation, Gene tables, Alignments of alleles). So far the number of higher vertebrate species present in the IMGT Web Resources reaches forty. The curated IG and TR genes and alleles are entered in the IMGT/GENE-DB database and the corresponding IMGT^®^ reference directories and used for coherent gene and sequence annotations of IG and TR loci of newly sequenced genomes. Thus the annotation of the IG and TR loci [78,322,323,324,325,326,327] are key to the study and comparison of the expressed adaptive immune repertoires, in normal or pathological situations. The IMGT standardized IG and TR genes and alleles in different species [328,329,330,331,332,333,334,335,336,337,338,339,340,341,342,343,344,345,346,347,348,349,350,351,352,353,354,355,356,357,358,359,360,361,362] offer a unique opportunity for comparison of immune responses and of potential applications in veterinary and human medicine.

IMGT/V-QUEST is the reference tool for the clonality sequence analysis in leukemia and lymphoma [291,292,293,294,363,364,365,366], which has been extended to veterinary species owing to the availability and IMGT^®^ biocuration of the IG and TR loci [346,349,350,351], and the IMGT reference directories. Exploring hight-throughput repertoires with IMGT/HighV-QUEST provides standardized NGS analysis of IG and TR repertoires in experimental engineered (combinatorial libraries) or in physiological conditions (vaccination, immunodeficiency, autoimmune diseases, cancers and infectious diseases). IMGT/HighV-QUEST is particularly well adapted for the analysis of complete V domains of the IG and TR repertoires from B and T subsets, in many experiments and from many individuals (humans or other vertebrate species). It allows the analysis of the content of scFv combinatorial phage display libraries which are classically screened for identification of novel therapeutic antibody specificities [367,368,369,370,371].

Given the importance of the interactions in the antibody specificity and affinity on the one hand and in the antibody pharmacokinetics/pharmacodynamics and half-life on the other hand, the IMGT^®^ integrated and standardized approach provides the genetic knowledge for allowing antibody informatics to answer the needs of targeted and customized therapy in the context of personalized medicine. The INN definition for the -mab integrates the format, target(s), IMGT gene and allele nomenclature, the CDR-IMGT lengths delimitation, the post-translational modifications and the engineered AA changes [60,86,87].

The extension of the IMGT unique numbering to the IgSF [372,373,374,375,376] and to the MhSF [377,378,379] proteins other than IG or TR has opened new perspectives for the standardized description of the polymorphism of the antigens (epitopes belonging to V, C or G domains) and of the Fc receptors (FCGR of the IgSF, FCGRT of the MhSF) and for the characterization of their interactions (antibody/antigen, FcR/antibody). The F domain (for Fibronectin type III domain), the S domain (for Scavenger, of the Scavenger receptor superfamly SrSF) and the A domain (for Apple domain) have been standardized with an IMGT unique numbering. Mass spectrometry has shown promising results in the analysis of the IGHG3 polymorphism and that anti-malarial variable domains [380,381,382]. Using the IMGT unique numbering per domain is the bridge between the biological and computational spheres.

## Figures and Tables

**Table 1:** Immunoglobulin (IG) receptor, chain and domain structure labels and correspondence with sequence labels [34]. IMGT standardized labels are in capital letters [34,35]. They are shown for two examples of IG: **A**. *Homo sapiens* IgG1-kappa, **B**. *Homo sapiens* IgM-lambda.

A.				
**IG Structure Labels** **(IMGT/3Dstructure-DB [57,58,59])**				**Sequence Labels (IMGT/LIGM-DB [54,55]** **)**
**Receptor**	**Chain**	**Domain Type**	**Domain**	**Region ^1^**
IG-GAMMA-1_KAPPA	H-GAMMA-1	V	VH	V-D-J-REGION
		C	CH1	C-REGION ^2^
		C	CH2	
		C	CH3	
	L-KAPPA	V	V-KAPPA	V-J-REGION
		C	C-KAPPA	C-REGION
B.				
**IG Structure Labels** **(IMGT/3Dstructure-DB) [57,58,59]**				**Sequence Labels (IMGT/LIGM-DB) [54,55]**
**Receptor**	**Chain**	**Domain type**	**Domain**	**Region ^1^**
IG-MU_LAMBDA	H-MU	V	VH	V-D-J-REGION
		C	CH1	C-REGION ^2^
		C	CH2	
		C	CH3	
		C	CH4 ^3^	
	L-LAMBDA	V	V-LAMBDA	V-J-REGION
		C	C-LAMBDA-1	C-REGION

^1.^ The VH domain (or V-D-J-REGION) and the VL domain (V-KAPPA or V-LAMBDA) (or V-J-REGION) are encoded by rearranged V-(D)-J genes, whereas the remaining of the chain is the C-REGION (encoded by a C gene). The C-REGION comprises one C domain (C-KAPPA or C-LAMBDA) for the L chain, or several C domains (CH) for the H chain. ^2.^ The heavy chain C-REGION also includes the HINGE-REGION for the H-ALPHA, H-DELTA and H-GAMMA chains and, for membrane IG (mIG), the CONNECTING-REGION (CO), TRANSMEMBRANE-REGION (TM) and CYTOPLASMIC-REGION (CY); for secreted IG (sIG), the C-REGION includes CHS instead of CO, TM and CY. ^3.^ For H-MU and H-EPSILON.

**Table 2:** Structural and biological properties of human immunoglobulins [2].

Properties	Human IG Classes and Subclasses								
	IgM	IgD	IgG				IgA		IgE
			IgG1	IgG2	IgG3	IgG4	IgA1	IgA2	
Molecular weight of secreted form (kDa) ^1^	950 (p)	170–180	150	150	155-165	150	160 (m) 300 (d)	160 (m) 350 (d)	190
Chain composition (with number of H2L2 monomers, if more than one)	(μ_2_κ_2_)5 or (μ_2_λ_2_)5	δ_2_κ_2_ or δ_2_λ_2_	γ1_2_κ_2_ or γ1_2_λ_2_	γ2_2_κ_2_ or γ2_2_λ_2_	γ3_2_κ_2_ or γ3_2_λ_2_	γ4_2_κ_2_ or γ4_2_λ_2_	(α1_2_κ_2_)1–2 or (α1_2_λ_2_)1–2	(α2_2_κ_2_)1–2 or (α2_2_λ_2_)1–2	ε_2_κ_2_ or ε_2_λ_2_
Functional valency (nb of antigen binding sites)	5 or 10	2	2				2 or 4		2
Structure ^2^	monomer (mb) pentamer (s)	monomer (mb, s)	monomer (mb, s)				monomer (mb, s) dimer (sec)		monomer (mb, s)
Inter-heavy (H-H) disulfide bridges per monomer	1	1	2	4	11	2	2	2	1
Other chain	J chain(16 kDa)	−	−				J chain (16 kDa), secretory component (70 kDa)		−
Sedimentation coefficient in Svedberg unit (S)	18–20	7	6.5–7.0				7, 10, 13, 15, 17		7.9
Carbohydrate average (%)	10–12	9–14	2–3				7–11		12–13
Adult level range (age 16-60) in serum (g/L) ^3^	0.25–3.1	0.03-0.4	5–12	2–6	0.5–1	0.2–1	1.4–4.2	0.2–0.5	0.0001–0.0002
			8.0-16.8						
Approximate % total IG in adult serum (% of the subclass in the class)	10	0.2	45–53 (70–80)	11–15 (18−23)	3–6 (6−8)	1–4 (2–6)	11–14 (90)	1–4 (10)	0.004
Synthetic rate (mg/kg weight/day)	3.3	0.2	33	33	33	33	19–29	3.3–5.3	0.002
Biological half-life (day)	5–10	2–8	21–24	21–24	7–8	21–24	5–7	4–6	1–5
Transplacental transfer	0	0	++	+	++	++	0	0	0
Complement activation classical pathway (C1q)	+++	0	++	+	+++	0	0	0	0
Complement activation alternative pathway	0	0	0	0	0	0	+	0	0
Binding macrophages and other phagocytic cells (FcγR or FCGR) ^4^	0	+	+++	+/–	+++	+/–	0	0	0
Binding to mast cells and basophils (FcεR or FCER)	0	0	0	0	0	0	0	0	+++
Binding to epithelial poly-IG receptor	+	0	0	0	0	0	+++	+++	0
Reactivity with *Staphyloccus* protein A	0	0	++	++	(0) *	++	0	0	0

* IgG3 allotype dependent; **^1.^** Approximate molecular weight (m = monomer, d = dimer, p = pentamer). ^2.^ mb = membrane, s = secreted, sec = secretory. ^3.^ Total IG adult level range (age 16–60) in serum: 9.5–21.7 g/L; ^4.^ +/–: binding depends on the FCGR isotype and on the cell type.

**Table 3:** *Homo sapiens* immunoglobulin (IG) chain characteristics [2].

Chain Characteristics			Human IG Chain Types										
			Heavy Chain (H)									Light Chain (L)	
			μ	δ	γ1	γ2	γ3	γ4	α1	α2	ε	κ	λ
Molecular weight (kDa)			70	60	50	50	53-57	50	55	55	70	25	25
Number of variable (V) domains			1	1	1	1	1	1	1	1	1	1	1
Number of constant (C) domains			4	3	3	3	3	3	3	3	4	1	1
Length (in number of amino acids)	Constant region	CH1 (for H) or CL (for L)	104	101	97	97	98	97	104	102	103	107	104 or 106
		CH2	112	108	109	108	110	109	101	101	107	−	−
		CH3	112	108	106	106	105	106	112	111	108	−	−
		CH4	111	−	−	−	−	−	−	−	110	−	−
	Hinge region		0	58	15	12	32, 47 or 62	12	19	6	0	−	−
	Membrane IG	Connecting region (CO)	13	27	18	18	18	18	32	32	15	−	−
		Transmembrane region (TM)	27	27	27	27	27	27	27	27	27	−	−
		Cytoplamic region (CY)	1	1	26	26	26	26	12	12	26	−	−
	Secreted IG	CHS ^1^	20	9	2	2	2	2	20	20	2	−	−
N-glycosylation sites ^2^			5	3	1	1	2	1	2	4	7	0	0
O-glycosylation sites ^3^			−	7	−	−	−	−	8	−	−	−	−
Allotypes [83]			−	−	G1m	G2m	G3m	G4m	−	A2m	Em	Km	−

^1.^ CHS corresponds to the tailpiece of secreted IG, encoded by the CHS region at the C-terminal end of the CH4 exon (for IGHM and IGHE) or of the CH3 exon (for IGHG1, IGHG2, IGHG3, IGHG4, IGHA1, IGHA2) or as a separate exon for IGHD. ^2.^ Only indicated for the C-REGION. N-glycosylation sites may be found in some V domains. ^3.^ O-glycosylation sites are located in the hinge regions of the H-delta and H-alpha1 chains.

**Table 4:** Interchain disulfide bridges. The number of inter Heavy (H)-Light (L) and inter H-H disulfide bridges is given per monomer of human (*Homo sapiens*) IG classes and subclasses, with positions of the cystein in the domain (IMGT unique numbering for C-DOMAIN [42]).

Disulfide Bridges	Chain		Classes and Subclasses								
			IgM	IgD	IgG				IgA		IgE
					IgG1	IgG2	IgG3	IgG4	IgA1	IgA2	
Inter H-L	H	number	1	1	1	1	1	1	1	1	1
		Positions ^1^	CH1 10	CH1 11	h 5	CH1 10	CH1 10	CH1 10	CH1 10	CH1 123	CH1 10, 11
	L		C-KAPPA	126 (last amino acid)							
			C-LAMBDA	126 (penultimate amino acid)							
Inter H-H	H	number	1	1	2	4	11	2	2	2	1
		Positions ^1^	CH2: 125	CH2: 1.6	h 11, h 14	h 4, h 5, h 8, h 11	h1 13, h1 16, h2 5, h2 11, h2 14, h3 5, h3 11, h3 14, h4 5, h4 11, h4 14	h 8, h 11	CH2: 1.1, 1.2	CH2: 1.1, 1.2	CH2: 1.6, 11, 124

^1^. Cysteines of the hinge involved in inter-chain bridges are indicated with a small letter ‘h’, for example, Inter-H-L (h 5-CL 126), and Inter-H-H (h 11, h 14), for an *Homo sapiens* IgG1 – kappa, as used in the description of monoclonal antibodies, published by the WHO INN programme [86,87]). h1 to h4 numbers the IGHG3 hinge exons in the most current haplotypes.

**Table 5:** Number of IG genes and alleles per IMGT group in IG major loci and orphon sets.

Locus		IGH					IGK				IGL				Total
IMGT Group		IGHV	IGHD	IGHJ	IGHC	Total IGH	IGKV	IGKJ	IGKC	Total IGK	IGLV	IGLJ	IGLC	Total IGL	
Nb of genes	Major Locus	159	27	9	11	206	77	5	1	83	78	11 ^1^	11 ^1^	100	389
	Orphon sets	26	10	0	1 ^2^	37	32	0	0	32	4 ^3^	0	3 ^4^	7	76
	Locus total	185	37	9	12	243	109	5	1	115	82	11	14	107	465
Nb of alleles	Major Locus	494	34	19	91	638	114	9	5	128	144	10	21	175	941
	Orphon sets	49	10	0	2	61	34	0		34	5	0	4	9	104
	Locus total	543	44	19	93	699	148	9	5	162	149	14	25	186	1045

^1.^ Includes IGLJ2A, IGLC2A, IGLJ2B, IGLC2B, IGLJ2C, IGLC2C, IGLJ2D, IGLC2D; detected by Southern blot analysis and mapped, no sequence available. ^2.^ Includes IGHEP2, the only known processed gene in an IG major locus in *Homo sapiens*. ^3.^ Not included IGLV8/Ora which is unmapped (detected by Southern blot analysis, no sequence available). ^4.^ Includes IGLJ-C/OR18, the only known processed IG orphon gene in *Homo sapiens*. (IMGT^®^, http://www.imgt.org, IMGT Repertoire (IG and TR) > Gene table: human (*Homo sapiens*)).

**Table 6:** The ten most classical IG and TR entity types and associated prototypes. Entity types are identified with IMGT standardized keywords [30] (IDENTIFICATION axiom [30,31]). Prototypes are described with IMGT standardized labels [34] (DESCRIPTION axiom [34,35]).

IDENTIFICATION (IMGT Standardized Keywords) [30]				DESCRIPTION (IMGT Standardized Labels) [34]
Molecular Entity Type	Configuration Type	Molecule Type	Functionality	Molecular Entity Prototype
V-gene	germline	gDNA	F, ORF, P	V-GENE
D-gene	germline	gDNA	F, ORF, P	D-GENE
J-gene	germline	gDNA	F, ORF, P	J-GENE
C-gene	undefined	gDNA	F, ORF, P	C-GENE
V-D-J-gene	rearranged	gDNA	productive, unproductive	V-D-J-GENE
V-J-gene	rearranged	gDNA	productive, unproductive	V-J-GENE
L-V-D-J-C-sequence	rearranged	cDNA	productive, unproductive	L-V-D-J-C-SEQUENCE
L-V-J-C-sequence	rearranged	cDNA	productive, unproductive	L-V-J-C-SEQUENCE
V-D-J-C-sequence or chain	rearranged	protein	productive, unproductive	H-MU, H-DELTA, H-GAMMA1, etc.
V-J-C-sequence or chain	rearranged	protein	productive, unproductive	L-KAPPA, L-LAMBDA1, etc.

**Table 7:** Potential *Homo sapiens* IGH genomic repertoire.

**A.***Homo sapiens* IGH V-CLUSTER.							
**IMGT IGHV Subgroup**	**IMGT Gene Name ^1^**	**CDR-IMGT**	**Nb of Alleles F**	**Nb of Alleles ORF**	**Nb of Alleles** **P**	***Homo Sapiens*** **IGHV-CNV**	**Gene Order**
IGHV1	IGHV1-2	[8.8.2]	6 F	−	−		159
	IGHV1-3	[8.8.2]	3 F, 1 (F)	−	−		157
	IGHV1-8	[8.8.2]	3 F	−	−	CNV5e1	147
	IGHV1-18	[8.8.2]	4 F	−	−		132
	IGHV1-24	[8.8.2]	1 F	−	−		120
	IGHV1-45	[8.8.2]	3 F	−	−		60
	IGHV1-46	[8.8.2]	4 F	−	−		59
	IGHV1-58	[8.8.2]	2 F, 1 (F)	−	−		41
	IGHV1-69 ^a^	[8.8.2]	17 F, 1 (F)	−	−		21
	IGHV1-69D ^a^	[8.8.2]		−	−	CNV1i	17
	IGHV1-69-2	[8.8.2]	2 F	−	−	CNV1i	18
IGHV2	IGHV2-5	[10.7.3]	8 F (no *07)	−	−		154
	IGHV2-26	[10.7.3]	3 F	−	−		117
	IGHV2-70 ^b^	[10.7.3]	19 F	1 ORF	−		16
	IGHV2-70D ^b^	[10.7.3]		−	−	CNV1i	20
IGHV3	IGHV3-7	[8.8.2]	3 F, 1 (F)	−	−		150
	IGHV3-9	[8.8.3]	3 F	−	−	CNV5e1	146
	IGHV3-11	[8.8.2]	5 F	−	1 P (0.1)		144
	IGHV3-13	[8.7.2]	5 F	−	−		141
	IGHV3-15	[8.10.2]	8 F	−	−		138
	IGHV3-20	[8.8.2]	2 F	2 ORF	−		130
	IGHV3-21	[8.8.2]	4 F, 1 (F)	−	−		128
	IGHV3-23 ^c^	[8.8.2]	*5* F	−	−		124
	IGHV3-23D ^c^	[8.8.2]		−	−	CNV4i	121
	IGHV3-30 ^d^	[8.8.2]	19 F	−	−		109
	IGHV3-30-3	[8.8.2]	3 F	−	−	CNV3i	102
	IGHV3-30-5 ^d^	[8.8.2]		−	−	CNV3i	96
	IGHV3-33	[8.8.2]	7 F	−	−		89
	IGHV3-43 ^e^	[8.8.3]	2 F	−	−		65
	IGHV3-43D ^e^	[8.8.3]	2 F	−	−	CNV2i	76
	IGHV3-48	[8.8.2]	4 F	−	−		55
	IGHV3-49	[8.10.2]	5 F	−	−		54
	IGHV3-53	[8.8.2]	4 F, 1 (F)	−	−		47
	IGHV3-62	[8.8.2]	1 F (* 04) ^2^	−	3 P (2.1)		36
	IGHV3-64 ^f^	[8.8.2]	7	−	−		32
	IGHV3-64D ^f^	[8.8.2]		−	−	CNV5e2	149
	IGHV3-66	[8.7.2]	4 F	−	−		29
	IGHV3-72	[8.10.2]	2 F	−	−		14
	IGHV3-73	[8.10.2]	2 F	−	−		13
	IGHV3-74	[8.8.2]	3 F	−	−		12
	IGHV3-NL1	[8.8.2]	1 F	−	−		0
IGHV4	IGHV4-4	[8.7.2]	7 F, 2 (F)	−	−		156
	IGHV4-28	[8.7.2]	7 F	−	−		113
	IGHV4-30-1 ^g^	[10.7.2]		−	−	CNV3i	106
	IGHV4-30-2	[10.7.2]	6 F	−	−	CNV3i	105
	IGHV4-30-4	[10.7.2]	7 F, 1 (F)	−	−	CNV3i	99
	IGHV4-31 ^g^	[10.7.2]	10 F, 1 (F)	−	−	CNV3d	93
	IGHV4-34	[8.7.2]	13 F	−	−		86
	IGHV4-38-2	[9.7.2]	2 F	−	−	CNV2i	79
	IGHV4-39	[10.7.2]	7 F	−	−		70
	IGHV4-59	[8.7.2]	12 F, 1 (F)	−	−		40
	IGHV4-61	[10.7.2]	8 F, 1 (F)	1 ORF	−		37
IGHV5	IGHV5-10-1	[8.8.2]	4 F	−	−	CNV5e2	148
	IGHV5-51	[8.8.2]	6 F, 1 (F)	−	−		51
IGHV6	IGHV6-1	[10.9.2]	2 F	−	1 P (1.0)		163
IGHV7	IGHV7-4-1	[8.8.2]	5 F	−	-	CNV6i	155
IGHV8	IGHV8-51-1	[8.8.2]	0 F	1 ORF	2 P (2.0)		
**B.***Homo sapiens* IGH D-J-C-CLUSTER.							
**D-J-C-CLUSTER**	**IMGT gene names**	**Nb of alleles F**		**Nb of alleles ORF and P**		**Gene order**	
IGHD	IGHD1-1	1 F		−		165	
	IGHD2-2	3 F		−		166	
	IGHD3-3	1 F		−		167	
	IGHD4-4	1 F		−		168	
	IGHD5-5	1 F		−		169	
	IGHD6-6	1 F		−		170	
	IGHD1-7	1 F		−		171	
	IGHD2-8	2 F		−		172	
	IGHD3-9	1 F		−		173	
	IGHD3-10	2 F		−		174	
	IGHD4-11	-		1 ORF		175	
	IGHD5-12	1 F		−		176	
	IGHD6-13	1 F		−		177	
	IGHD1-14	-		1 ORF		178	
	IGHD2-15	1 F		−		179	
	IGHD3-16	2 F		−		180	
	IGHD4-17	1 F		−		181	
	IGHD5-18	1 F		−		182	
	IGHD6-19	1 F		−		183	
	IGHD1-20	1 F		−		184	
	IGHD2-21	2 F		−		185	
	IGHD3-22	1 F		−		186	
	IGHD4-23	−		1 ORF		187	
	IGHD5-24	−		1 ORF		188	
	IGHD6-25	1 F		−		189	
	IGHD1-26	1 F		−		190	
	IGHD7-27	1 F		−		192	
IGHJ	IGHJ1	1 F		−		193	
	IGHJ2	1 F		−		194	
	IGHJ3	2 F		−		196	
	IGHJ4	3 F		−		197	
	IGHJ5	2 F		−		198	
	IGHJ6	4 F		−		200	
IGHC	IGHM	4 F		−		201	
	IGHD	2 F		−		202	
	IGHG3	29 F		−		203	
	IGHG1	12 F, 2 (F)		−		204	
	IGHEP1	-		4 P		205	
	IGHA1	3 F		−		206	
	IGHGP	-		2 ORF, 1P		207	
	IGHG2	17 F		−		208	
	IGHG4	8 F		−		209	
	IGHE	4 F		−		210	
	IGHA2	3 F		−		211	

^1.^ Sequences of the polymorphic genes which cannot be differentiated from each other are displayed in the same Alignments of alleles (http://www.imgt.org). They correspond to 7 pairs: ^a^ IGHV1-69/IGHV1-69D, ^b^ IGHV2-70/IGHV2-70D, ^c^ IGHV3-23/IGHV3-23D, ^d^ IGHV3-30/IGHV3-30-5, ^e^ IGHV3-43/IGHV3-43D, ^f^ IGHV3-64/IGHV3-64D, ^g^ IGHV4-30-1/IGHV4-31. ^2.^ Since 2001, the five unmapped genes [2] have been named. They correspond to two ORF, IGHV1-38-4 (1-c) and IGHV3-38-3 (3-d) and to three F, IGHV1-69-2 (1-f), IGHV4-38-2 (4-b) and IGHV5-10-1 (5-a). An asterisk (*) indicates the functional allele of IGHV3-62.

**Table 8:** Potential *Homo sapiens* IGK genomic repertoire. A. *Homo sapiens* IGK V-CLUSTER. B. *Homo sapiens* IGK J-C-CLUSTER

A.										
**IMGT IGKV Subgroup**		**IMGT Gene Name**		**CDR-IMGT**	**Nb of Alleles F**	**Nb of Alleles ORF**	**Nb of Alleles P**	**Cluster**		**Gene Order**
IGKV1		IGKV1-5		[6.3.7]	3 F	-	-	proximal		72
		IGKV1-6		[6.3.7]	2F	-	-	proximal		71
		IGKV1-8		[6.3.7]	1 F	-	-	proximal		69
		IGKV1-9		[6.3.7]	1 F	-	-	proximal		68
		IGKV1-12		[6.3.7]	2 F	-	-	proximal		65
		IGKV1-13		[6.3.7]	1 F	-	1 P (1.0)	proximal		64
		IGKV1-16		[6.3.7]	1 F, 1 [F]	-	-	proximal		61
		IGKV1-17		[6.3.7]	3 F	-	-	proximal		60
		IGKV1-27		[6.3.7]	1 F	-	-	proximal		50
		IGKV1-33		[6.3.7]	1 F	-	-	proximal		44
		IGKV1-39		[6.3.7]	1 F	-	1 P (1.0)	proximal		38
		IGKV1D-8		[6.3.7]	3 F	-	-	distal		2
		IGKV1D-12		[6.3.7]	2 F	-	-	distal		7
		IGKV1D-13		[6.3.7]	2 F	-	-	distal		8
		IGKV1D-16		[6.3.7]	2 F	-	-	distal		11
		IGKV1D-17		[6.3.7]	1F	-	-	distal		12
		IGKV1D-33		[6.3.7]	1 F	-	-	distal		29
		IGKV1D-39		[6.3.7]	1 F	-	-	distal		35
		IGKV1D-43		[6.3.7]	1 F	-	-	distal		3
		IGKV1-NL1		[6.3.7]	1 F	-	-			0
IGKV2		IGKV2-24		[11.3.7]	1 F	-	-	proximal		53
		IGKV2-28		[11.3.7]	1 F	-	-	proximal		49
		IGKV2-29		[11.3.7]	2 F	-	1 P (1.0)	proximal		48
		IGKV2-30		[11.3.7]	1 F, 1 [F]	-	-	proximal		47
		IGKV2-40		[12.3.7]	2 F	-	-	proximal		37
		IGKV2D-26		[11.3.7]	3 F	-	-	distal		22
		IGKV2D-28		[11.3.7]	1 F	-	-	distal		24
		IGKV2D-29		[11.3.7]	2 F	-	-	distal		25
		IGKV2D-30		[11.3.7]	1 F	-	-	distal		26
		IGKV2D-40		[12.3.7]	1 F	-	-	distal		36
IGKV3		IGKV3-11		[6.3.7]	2 F	-	-	proximal		66
		IGKV3-15		[6.3.7]	1 F	-	-	proximal		62
		IGKV3-20		[7.3.7]	2 F	-	-	proximal		57
		IGKV3D-7		[7.3.7]	1 F	-	-	distal		1
		IGKV3D-11		[6.3.7]	3 F	-	-	distal		6
		IGKV3D-15		[6.3.7]	2 F	-	1 P (1.0)	distal		10
		IGKV3D-20		[7.3.7]	1 F	1 ORF	-	distal		16
IGKV4		IGKV4-1		[12.3.7]	1 F	-	-	proximal		76
IGKV5		IGKV5-2		[6.3.7]	1 F	-	-	proximal		75
IGKV6		IGKV6-21		[6.3.7]	2 F	-	-	proximal		56
		IGKV6D-21		[6.3.7]	2 F	-	-	distal		17
B.										
**J-C-CLUSTER**	**IMGT Gene Names**		**Nb of Alleles F**			**Nb of Alleles ORF, P**			**Gene Order**	
IGKJ	IGKJ1		1 F			-			77	
	IGKJ2		1 F, 3 (F)			-			78	
	IGKJ3		1 F			-			79	
	IGKJ4		1F, 1 (F)			-			80	
	IGKJ5		1 F			-			81	
IGKC	IGKC		4 F, 1 (F)			-			82	

**Table 9:** Potential *Homo sapiens* IGL genomic repertoire. A. *Homo sapiens* IGL V-CLUSTER. B. *Homo sapiens* IGL J-C-CLUSTER.

A.							
**IMGT IGLV Subgroup**	**IMGT Gene Name**	**CDR-IMGT**	**Nb of Alleles F**	**Nb of Alleles ORF**	**Nb of Alleles P**	**Cluster**	**Gene Order**
IGLV1	IGLV1-36	[8.3.9]	1 F	−	−	B	40
	IGLV1-40	[9.3.9]	3 F	−	−	B	36
	IGLV1-44	[8.3.9]	1 F	−	−	B	31
	IGLV1-47	[8.3.9]	2 F	−	−	B	28
	IGLV1-51	[8.3.9]	2 F	−	−	B	24
IGLV2	IGLV2-8	[9.3.9]	3 F	−	−	A	77
	IGLV2-11	[9.3.9]	3 F	−	−	A	74
	IGLV2-14	[9.3.9]	4 F	−	−	A	71
	IGLV2-18	[9.3.9]	4 F	−	−	A	67
	IGLV2-23	[9.3.9]	3 F	−	−	A	61
IGLV3	IGLV3-1	[6.3.7]	1 F	−	−	A	84
	IGLV3-9	[6.3.7]	2 F	−	1 P (0.1)	A	76
	IGLV3-10	[6.3.9]	2 F	−	−	A	75
	IGLV3-12	[6.3.9]	2 F	−	−	A	73
	IGLV3-16	[6.3.9]	1 F	−	−	A	69
	IGLV3-19	[6.3.9]	1 F	−	−	A	66
	IGLV3-21	[6.3.9]	3 F	−	−	A	64
	IGLV3-22	[6.3.7]	1 F	−	1 P (0.1)	A	63
	IGLV3-25	[6.3.9]	3 F	−	−	A	59
	IGLV3-27	[6.3.7]	1 F	−	−	A	56
IGLV4	IGLV4-3	[7.7.12]	1 F	−	−	A	82
	IGLV4-60	[7.7.7]	3 F	−	−	C	13
	IGLV4-69	[7.7.7]	2 F	−	−	C	3
IGLV5	IGLV5-37	[9.7.8]	1 F	−	−	B	39
	IGLV5-39	[9.7.8]	2 F	−	−	B	37
	IGLV5-45	[9.7.8]	4 F	−	−	B	30
	IGLV5-52	[9.7.9]	1 F	−	−	B	23
IGLV6	IGLV6-57	[8.3.7]	2 F	−	−	C	17
IGLV7	IGLV7-43	[9.3.8]	1 F	−	−	B	32
	IGLV7-46	[9.3.8]	2 F		1 P (0.1)	B	29
IGLV8	IGLV8-61	[9.3.8]	3 F		−	C	12
IGLV9	IGLV9-49	[7.8.12]	3 F		−	B	26
IGLV10	IGLV10-54	[8.3.9]	2 F		1 P (1.0)	C	20
B.							
**J-C-CASSETTE**	**IMGT Gene Name**	**Nb of Alleles F**		**Nb of Alleles ORF, P**		**Gene Order**	
1	IGLJ1	1 F		−		85	
	IGLC1	1 F		1 ORF		86	
2	IGLJ2	1 F		−		87	
	IGLC2	3 F		−		88	
3	IGLJ3	2 F		−		89	
	IGLC3	4 F		−		90	
4	IGLJ4	−		1 ORF		91	
	IGLC4	−		2 P		92	
5	IGLJ5	−		2 ORF		93	
	IGLC5	−		2 P		94	
6	IGLJ6	1 F		−		95	
	IGLC6	1 F		4 P		96	
7	IGLJ7	2F		−		97	
	IGLC7	5 F		−		98	

**Table 10:** V domain strands and loops and IMGT positions and lengths, based on the IMGT unique numbering for V domain (V-DOMAIN and V-LIKE-DOMAIN) [40,41].

V Domain Strands and Loops ^1^	IMGT Positions ^2^	Lengths ^3^	Characteristic IMGT Residue@Position ^4^	V-DOMAIN FR-IMGT and CDR-IMGT
A-STRAND	1–15	15 (14 if gap at 10)		FR1-IMGT
B-STRAND	16–26	11	1st-CYS 23	
BC-LOOP	27–38	12 (or less)		CDR1-IMGT
C-STRAND	39–46	8	CONSERVED-TRP 41	FR2-IMGT
C′-STRAND	47–55	9		
C′C″-LOOP	56–65	10 (or less)		CDR2-IMGT
C″-STRAND	66–74	9 (or 8 if gap at 73)		FR3-IMGT
D-STRAND	75–84	10 (or 8 if gaps at 81, 82)		
E-STRAND	85–96	12	hydrophobic 89	
F-STRAND	97–104	8	2nd-CYS 104	
FG-LOOP	105–117	13 (or less, or more)		CDR3-IMGT
G-STRAND	118–128	11 (or 10)	V-DOMAIN J-PHE 118 or J-TRP 118 ^5^	FR4-IMGT

^1.^ IMGT^®^ labels (concepts of description) are written in capital letters (no plural) [34,35]. Beta turns (AB, CC’, C”D, DE or EF) are individualized only if they have additional amino acids compared to the standard description. If not, they are included in the strands. ^2.^ based on the IMGT unique numbering for V domain (V-DOMAIN and V-LIKE-DOMAIN) [40,41,44,45,46]). ^3.^ in number of amino acids (or codons). ^4.^ IMGT Residue@Position is a given residue (usually an amino acid) or a given conserved property amino acid class, at a given position in a domain, based on the IMGT unique numbering [44,45,46]. ^5.^ In the IG and TR V-DOMAIN, the G-STRAND (or FR4-IMGT) is the C-terminal part of the J-REGION, with J-PHE or J-TRP 118 and the J-MOTIF F/W-G-X-G at positions 118-121 [2,3]. The JUNCTION refers to the CDR3-IMGT plus the two anchors 2nd-CYS 104 and J-PHE or J-TRP 118 [40,41]. The JUNCTION (positions 104-118) is therefore two amino acids longer than the corresponding CDR3-IMGT (positions 105-117) [40,41].

**Table 11:** IMGT additional positions for CDR3-IMGT.

CDR3-IMGT Lengths	IMGT Additional Positions for CDR3-IMGT Length > 13 AA ^1^									
---										
21	111	111.1	111.2	111.3	111.4	112.4	112.3	112.2	112.1	112
20	111	111.1	111.2	111.3	−	112.4	112.3	112.2	112.1	112
19	111	111.1	111.2	111.3	−	−	112.3	112.2	112.1	112
18	111	111.1	111.2	−	−	−	112.3	112.2	112.1	112
17	111	111.1	111.2	−	−	−	−	112.2	112.1	112
16	111	111.1	−	−	−	−	−	112.2	112.1	112
15	111	111.1	−	−	−	−	−	−	112.1	112
14	111	−	−	−	−	−	−	−	112.1	112

^1.^ For CDR3-IMGT length > 13 AA, IMGT additional positions are created between positions 111 and 112 at the top of the CDR3-IMGT loop in the following order 112.1, 111.1, 112.2, 111.2, 112.3, 111.3, etc. For CDR3-IMGT length < 13 AA (not shown), IMGT gaps are created classically from the top of the loop, in the following order 111, 112, 110, 113, 109, 114, etc. The top row ‘…’ above ‘21’ indicates that the same numbering rule is applied to CDR3-IMGT lengths > 21 AA. For very long CDR3-IMGT, tables are available in the IMGT Scientific chart (IMGT^®^ http://www.imgt.org, IMGT Scientific chart > 2. Numbering > IMGT unique numbering for V-DOMAIN and V-LIKE-DOMAIN).

**Table 12:** C domain strands, turns and loops and IMGT positions and lengths, based on the IMGT unique numbering for C domain (C-DOMAIN and C-LIKE-DOMAIN) [42].

C Domain Strands, Turns and Loops ^1^	IMGT Positions ^2^	Lengths ^3^	Characteristic IMGT Residue@Position ^4^
A-STRAND	1–15	15 (14 if gap at 10)	
AB-TURN	15.1–15.3	0–3	
B-STRAND	16–26	11	1st-CYS 23
BC-LOOP	27–31 34–38	10 (or less)	
C-STRAND	39–45	7	CONSERVED-TRP 41
CD-STRAND	45.1–45.9	0–9	
D-STRAND	77–84	8 (or 7 if gap at 82)	
DE-TURN	84.1–84.7 85.1–85.7	0–14	
E-STRAND	85–96	12	hydrophobic 89
EF-TURN	96.1–96.2	0–2	
F-STRAND	97–104	8	2nd-CYS 104
FG-LOOP	105–117	13 (or less, or more)	
G-STRAND	118–128	11 (or less)	

^1.^ IMGT^®^ labels (concepts of description) are written in capital letters, no plural) [34,35]. ^2.^ based. n the IMGT unique numbering for C domain (C-DOMAIN and C-LIKE-DOMAIN) [42,44,45,46]. ^3.^ in number of amino acids (or codons). ^4.^ IMGT Residue@Position is a given residue (usually an amino acid) or a given conserved property amino acid class, at a given position in a domain, based on the IMGT unique numbering [42,44,45,46].

**Table 13:** Content of the eleven IMGT/HighV-QUEST results files.

File Number ^1^	File Name	Number of Columns	Results Content ^2^
#1	Summary	25–29	Identity percentage with the closest V, D and J genes and alleles, FR-IMGT and CDR-IMGT lengths, Amino acid (AA) JUNCTION, Description of insertions and deletions if any.
#2	IMGT-gapped-nt-sequences	18	Nucleotide (nt) sequences gapped according to the IMGT unique numbering for the labels V-D-J-REGION, V-J-REGION, V-REGION, FR1-IMGT, CDR1-IMGT, FR2-IMGT, CDR2-IMGT, FR3-IMGT, nt sequences of CDR3-IMGT, JUNCTION, J-REGION and FR4-IMGT.
#3	Nt-sequences	63–78	nt sequences of all labels that can been automatically annotated by IMGT/Automat.
#4	IMGT-gapped-AA-sequences	18	AA sequences gapped according to the IMGT unique numbering for the labels V-D-J-REGION, V-J-REGION, V-REGION, FR1-IMGT, CDR1-IMGT, FR2-IMGT, CDR2-IMGT, FR3-IMGT, AA sequences of CDR3-IMGT, JUNCTION, J-REGION and FR4-IMGT.
#5	AA-sequences	18	Same columns as ‘IMGT-gapped-AA-sequences’ (#4), but sequences of labels are without IMGT gaps.
#6	Junction	33, 46, 66 or 77	Results of IMGT/JunctionAnalysis (JUNCTION, CDR3, N regions, trimmed V (D)J nucleotides, P regions, D regions and D-REGION reading frame (for IGH, TRB and TRD sequences), molecular mass, pI.
#7	V-REGION-mutation-and-AA-change table	11	List of mutations (nt mutations, AA changes, AA class identity (+) or change (−)) for V-REGION, FR1-IMGT, CDR1-IMGT, FR2-IMGT, CDR2-IMGT, FR3-IMGT and germline CDR3-IMGT.
#8	V-REGION-nt-mutation-statistics	130	Number (nb) of nt positions including IMGT gaps, nb of nt, nb of identical nt, total nb of mutations, nb of silent mutations, nb of nonsilent mutations, nb of transitions (a > g, g > a, c > t, t > c) and nb of transversions (a > c, c > a, a > t, t > a, g > c, c > g, g > t, t > g) for V-REGION, FR1-IMGT, CDR1-IMGT, FR2-IMGT, CDR2-IMGT, FR3-IMGT and germline CDR3-IMGT.
#9	V-REGION-AA-change-statistics	189	nb of AA positions including IMGT gaps, nb of AA, nb of identical AA, total nb of AA changes, nb of AA changes according to AAclassChangeType (+++, ++−, +−+, +−−, −+−, −−+, −−−), and nb of AA class changes according to AAclassSimilarityDegree (nb of Very similar, nb of Similar, nb of Dissimilar, nb of Very dissimilar) for V-REGION, FR1-IMGT, CDR1-IMGT, FR2-IMGT, CDR2-IMGT, FR3-IMGT and germline CDR3-IMGT.
#10	V-REGION-mutation-hotspots	8	Hot spots motifs ((a/t) a, t (a/t), (a/g) g (c/t) (a/t), (a/t) (a/g) c (c/t) detected in the closest germline V-REGION with positions in FR-IMGT and CDR-IMGT.
#11	Parameters		Date of the analysis, IMGT/V-QUEST programme version, IMGT/V-QUEST reference directory release, Parameters used for the analysis: species, receptor type or locus, IMGT reference directory set and Advanced parameters.

^1.^ Files #1 to #10 comprise systematically sequence identification (name, functionality, and names of the closest V, D and J genes and alleles [2,3,4,5]. The files #7 to #10 that report the analysis of mutations are used mostly for immunoglobulins (IG). (With permission from M-P. Lefranc and G. Lefranc, LIGM, Founders and Authors of IMGT^®^, the international ImMunoGeneTics information system^®^, http://www.imgt.org). ^2^. IMGT/HighV-QUEST statistical analysis and characterization of the IMGT clonotypes (AA).

**Table 14:** Correspondence between the IGHG1 alleles and G1m alleles [83,100].

IGHG1 Alleles	G1m Alleles ^1^		IMGT Amino Acid Positions ^2^								Populations [83]
	Allotypes	Isoallotype ^3^	CH1		CH3						
			103	120	12	14	101	110	115	116	
				G1m17/ nG1m17	G1m1/ nG1m1		/G1m27	/G1m2	/G1m28 -		
			G1m3 ^4^								
IGHG1*01 ^5^, IGHG1*02 ^5^, IGHG1*05 ^5^	**G1m17,1**		**I**	**K**	**D**	**L**	**V**	**A**	**H**	Y	Caucasoid Negroid Mongoloid
IGHG1*03	G1m3	*nG1m1*, *nG1m17*	**I**	**R**	E	M	V	A	H	Y	Caucasoid
IGHG1*04	G1m17,1,27		I	**K**	**D**	**L**	**I**	A	H	Y	Negroid
IGHG1*05p ^6^	G1m17,1,28		I	**K**	**D**	**L**	V	A	**R**	**Y**	Negroid
IGHG1*06p ^6^	G1m,17,1,27,28		I	**K**	**D**	**L**	**I**	A	**R**	**Y**	Negroid
IGHG1*07p ^6^	G1m17,1,2		I	**K**	**D**	**L**	V	**G**	H	Y	Caucasoid Mongoloid
IGHG1*08p ^6^	G1m3,1	*nG1m17*	**I**	**R**	**D**	**L**	V	A	H	Y	Mongoloid

^1.^ In Negroid populations, the G1m17,1 allele frequently includes G1m27 and/or G1m28, leading to three additional G1m alleles, G1m17,1,27, G1m17,1,28 and G1m17,1,27,28 [83]. ^2.^ Amino acids corresponding to G1m allotypes are shown in bold. ^3.^ The nG1m1 and nG1m17 isoallotypes present on the Gm1-negative and Gm-17 negative H-gamma-1 chains (and on other H-gamma chains) are shown in italics. ^4.^ The presence of R120 is detected by anti-nG1m17 antibodies whereas the simultaneous presence of I103 and R120 in the H-gamma1 chains is detected by anti-Gm3 antibodies [83]. ^5.^ The IGHG1*01, IGHG1*02 and IGHG1*05 alleles only differ at the nucleotide level (codon 85.1 in CH2 of *02 and *05 differs from *01, codon 19 in CH1 and codon 117 in CH3 of *05 differ from *01 and *02). ^6.^ IGHG1*05p, IGHG1*06p, IGHG1*07p and IGHG1*08p amino acids are expected [83] but not yet sequenced at the nucleotide level and therefore these alleles are not shown in Alignments of alleles (IMGT^®^ http://www.imgt.org, IMGT Repertoire > 2. Proteins and alleles > 2. Alignments of alleles > IGHC Mammalia > *Homo sapiens* IGHG1). Allotypes on CH1 include G1m17 K120 (blue) vs nG1m17, G1m3 R120 (dark blue). Allotypes on CH3 include G1m1 D12, L14 (yellow) vs nG1m1 E12 M14 (pale yellow), G1m27 I101 (green), G1m2 G110 (purple), G1m28 R115, Y116 (orange). (With permission from M-P. Lefranc and G. Lefranc, LIGM, Founders and Authors of IMGT^®^, the international ImMunoGeneTics information system^®^, http://www.imgt.org).

**Table 15:** IMGT engineered variant nomenclature [311]. *Homo sapiens* IGHG variants involved in ADCC, ADCP, CDC, half life increase, half-IG exchange, B cell inhibition, and knobs-into-holes are shown. In mAb description, the Eu numbering between parentheses is replaced by the amino position in the antibody gamma chain. Amino acid changes and bibliographical references are quoted at The IMGT Biotechnology page (IMGT^®^ http://www.imgt.org The IMGT Biotechnology page > Amino acid positions involved in ADCC, ADCP, CDC, half-life and half-IG exchange). Properties modifications include: ADCC or CDC enhancement (pale green), ADCP enhancement (dark green), ADCC or CDC reduction (pale orange), B cell inhibition (orange), Half-life increase (pale blue), Hald-IG exchange reduction, Hole or Knob in knobs-into-holes interaction, Favors hexamerisation, Site-specific drug attachment, No N-glycosylation site, No disulfide bridge inter H-L (yellow).

Species and IMGT Gene Name	IMGT Engineered Variant Nomenclature	IGHG Gene Variant Description				Property Modifications		
		CH2	AA and IMGT Position in CH2 of IGHG Gene Variant	CH3	AA and IMGT Position in CH3 of IGHG Gene Variant	ADCC Enhancement or Reduction, ADCP Enhancement, B Cell Inhibition	CDC Enhancement or Reduction	Half-IG Exchange Reduction, Half-Life Increase, Knobs-into-Holes
*CDC enhnHomo sapiens* IGHG1	IGHG1v1	CH2	P1.4 (233)			ADCC reduction		
	IGHG1v2	CH2	V1.3 (234)			ADCC reduction		
	IGHG1v3	CH2	A1.2 (235)			ADCC reduction		
	IGHG1v4	CH2	A114 (329)			ADCC reduction	CDC reduction	
	IGHG1v5	CH2	W109 (326)			ADCC reduction	CDC enhancement	
	IGHG1v6	CH2	A85.4 (298), A118 (333), A119 (334)			ADCC enhancement		
	IGHG1v7	CH2	D3 (239), E117 (332)			ADCC enhancement		
	IGHG1v8	CH2	D3 (239), L115 (330), E117 (332)			ADCC enhancement	CDC reduction	
	IGHG1v9	CH2	L7 (243), P83 (292), L85.2 (300), I88 (305)	CH3	L83 (396)	ADCC enhancement		
	IGHG1v10	CH2	Y1.3 (234), Q1.2 (235), W1.1 (236), M3 (239), D30 (268), E34 (270), A85.4 (298)			ADCC enhancement		
	IGHG1v11	CH2	E34 (270), D109 (326), M115 (330), E119 (334)			ADCC enhancement		
	IGHG1v12	CH2	A1.1 (236), D3 (239), L115 (330), E117 (332)			ADCC enhancement		
	IGHG1v13	CH2	A1.1 (236), D3 (239), E117 (332)			ADCP enhancement		
	IGHG1v14	CH2	A1.3 (234), A1.2 (235)			ADCC reduction	CDC reduction	
	IGHG1v15	CH2	S118 (333)				CDC enhancement	
	IGHG1v16	CH2	W109 (326), S118 (333)				CDC enhancement	
	IGHG1v17	CH2	E29 (267), F30 (268), T107 (324)				CDC enhancement	
	IGHG1v18			CH3	R1 (345), G109 (430), Y120 (440)		CDC enhancement	Favors hexamerisation
	IGHG1v19	CH2	A34 (270)				CDC reduction	
	IGHG1v20	CH2	A105 (322)				CDC reduction	
	IGHG1v21	CH2	Y15.1 (252), T16 (254), E18 (256)					Half-life increase
	IGHG1v22	CH2	Y15.1 (252), T16 (254), E18 (256)	CH3	K113 (433), F114 (434), H116 (436)			Half-life increase
	IGHG1v23	CH2	E1.2 (235)			ADCC reduction	CDC reduction	
	IGHG1v24			CH3	L107 (428), S114 (434)			Half-life increase
	IGHG1v25	CH2	E29 (267), F113 (328)			B cell inhibition		
	IGHG1v26			CH3	Y22 (366),			Knob in knobs-into-holes interaction
	IGHG1v27	CH2	C3 (239)					Site-specific drug attachment
	IGHG1v28	CH2	insC3A (239^240)					Site-specific drug attachment
	IGHG1v29	CH2	A84.4 (297)					No N-glycosylation site
	IGHG1v30	CH2	G84.4 (297)					No N-glycosylation site
	IGHG1v31			CH3	T86 (407)			Hole in knobs-into-holes interaction
	IGHG1v32			CH3	W22 (366)			Knob in knobs-into-holes interaction
	IGHG1v33			CH3	S22 (366), A24 (368), V86 (407)			Hole in knobs-into-holes interaction
	IGHG1v34			CH3	G109 (430)			Favors hexamerisation
	IGHG1v35	CH2	E29 (267)				CDC enhancement	
	IGHG1v36	CH2	Q84.4 (297)					No N-glycosylation site
	IGHG1v37	h	S5 (220)					No disulfide bridge inter H-L
	IGHG1v38	CH2	S108 (325), F113 (328)			Abrogation of FcyRIII binding	Abrogation of C1q bnding	
*Homo sapiens* IGHG2	IGHG2v1	CH2	L1.3 (234), L1.2 (235), G1.1 (236), G1 (237)			ADCC enhancement		
	IGHG2v2	CH2	Q30 (268), L92 (309), S115 (330), S116 (S331)			ADCC reduction	CDC reduction	
	IGHG2v3	CH2	A1.2 (235), A1 (237), S2 (238), A30 (268), L92 (309), S115 (330), S116 (331)			ADCC reduction	CDC reduction	
	IGHG2v4	CH2	Q14 (250)					Half-life increase
	IGHG2v5			CH3	L107 (428)			Half-life increase
	IGHG2v6	CH2	Q14 (250)	CH3	L107 (428)			Half-life increase
*Homo sapiens* IGHG3	IGHG3v1			CH3	H115 (435)			Half-life increase
*Homo sapiens* IGHG4	IGHG4v1	CH2	L1.3 (234)			ADCC enhancement		
	IGHG4v2	CH2	P116 (331)				CDC enhancement	
	IGHG4v3	CH2	E1.2 (235)			ADCC reduction	CDC reduction	
	IGHG4v4	CH2	A1.3 (234), A1.2 (235)			ADCC reduction	CDC reduction	
	IGHG4v5	h	P10 (228)					Half-IG exchange reduction
	IGHG4v6			CH3	K88 (409)			Half-IG exchange reduction
	IGHG4v21	CH2	Y15.1 (252), T16 (254), E18 (256)					Half-life increase
	IGHG4v36	CH2	Q84.4 (297)					No N-glycosylation site

(With permission from M-P. Lefranc and G. Lefranc, LIGM, Founders and Authors of IMGT^®^, the international ImMunoGeneTics information system^®^, http://www.imgt.org).
